# Combined proteomics and CRISPR‒Cas9 screens in PDX identify ADAM10 as essential for leukemia in vivo

**DOI:** 10.1186/s12943-023-01803-0

**Published:** 2023-07-08

**Authors:** Ehsan Bahrami, Jan Philipp Schmid, Vindi Jurinovic, Martin Becker, Anna-Katharina Wirth, Romina Ludwig, Sophie Kreissig, Tania Vanessa Duque Angel, Diana Amend, Katharina Hunt, Rupert Öllinger, Roland Rad, Joris Maximilian Frenz, Maria Solovey, Frank Ziemann, Matthias Mann, Binje Vick, Christian Wichmann, Tobias Herold, Ashok Kumar Jayavelu, Irmela Jeremias

**Affiliations:** 1 Research Unit Apoptosis in Hematopoietic Stem Cells, Helmholtz Center Munich, Feodor-Lynen-Str. 21, Munich, 81377 Germany; 2 German Cancer Consortium (DKTK), partner site Munich, Munich, Germany; 3 Laboratory for Experimental Leukemia and Lymphoma Research (ELLF), Department of Medicine III, LMU University Hospital, LMU Munich, Munich, Germany; 4 Division of Transfusion Medicine, Cell Therapeutics and Haemostaseology, LMU University Hospital, LMU Munich, Munich, Germany; 5 Center for Translational Cancer Research (TranslaTUM), TUM School of Medicine, and Department of Medicine II, Klinikum rechts der Isar, Technische Universität München, Munich, Germany; 6 Institute of Molecular Oncology and Functional Genomics, Technische Universität München, Munich, Germany; 7 Proteomics and Cancer Cell Signaling Group, German Cancer Research Center (DKFZ), Heidelberg, Germany; 8 Department of Pediatric Oncology, Hematology, and Immunology, University of Heidelberg and Hopp Children’s Cancer Center (KiTZ), Heidelberg, Germany; 9 Institute of Computational Biology, Helmholtz Center Munich, Munich, Germany; 10 Chair of Physiological Chemistry, Biomedical Center (BMC), Faculty of Medicine, LMU Munich, Munich, Germany; 11 Department of Proteomics and Signal Transduction, Max-Planck-Institute of Biochemistry, Munich, Germany; 12 Department of Pediatrics, Dr. Von Hauner Children’s Hospital, LMU University Hospital, LMU Munich, Munich, Germany

**Keywords:** CRISPR-Cas9 in vivo screen, Proteomics, ADAM10, PDX, Acute leukemia, Leukemia stem cells

## Abstract

**Background:**

Acute leukemias represent deadly malignancies that require better treatment. As a challenge, treatment is counteracted by a microenvironment protecting dormant leukemia stem cells.

**Methods:**

To identify responsible surface proteins, we performed deep proteome profiling on minute numbers of dormant patient-derived xenograft (PDX) leukemia stem cells isolated from mice. Candidates were functionally screened by establishing a comprehensive CRISPR‒Cas9 pipeline in PDX models in vivo.

**Results:**

A disintegrin and metalloproteinase domain-containing protein 10 (ADAM10) was identified as an essential vulnerability required for the survival and growth of different types of acute leukemias in vivo, and reconstitution assays in PDX models confirmed the relevance of its sheddase activity. Of translational importance, molecular or pharmacological targeting of ADAM10 reduced PDX leukemia burden, cell homing to the murine bone marrow and stem cell frequency, and increased leukemia response to conventional chemotherapy in vivo.

**Conclusions:**

These findings identify ADAM10 as an attractive therapeutic target for the future treatment of acute leukemias.

**Supplementary Information:**

The online version contains supplementary material available at 10.1186/s12943-023-01803-0.

## Highlights

- Ultra-deep proteomics of minute leukemia stem cells uncovers increased cell adhesion.

- In vivo PDX CRISPR-Cas9 screens identify ADAM10 as essential for leukemia in mice.

- ADAM10 knockout impairs the leukemia-niche interaction and stem cell numbers.

- Therapeutic targeting of ADAM10 reduces PDX leukemia fitness and augments therapy.

**Supplementary Information:**

The online version contains supplementary material available at 10.1186/s12943-023-01803-0.

## Introduction

The survival rates of adult and relapsed pediatric patients with acute leukemias (AL) remain dismal, and better treatment options are required [1, 2]. Leukemia depends on interaction with the normal in vivo microenvironment, and signals from surrounding bone marrow (BM) maintain dormant leukemia stem cells (LSCs) and protect them from therapy [3–5]. We previously showed in both acute leukemia of the lymphoblastic (ALL) and myeloid (AML) lineages that retrieving dormant, resistant leukemia cells from the niche sensitizes them toward treatment [6, 7].

Here, we aimed to identify regulators of leukemia maintenance in the protective BM environment that may represent novel therapeutic targets. We used in vivo approaches and studied patient-derived xenograft (PDX) models that closely mimic human leukemic disease [8–14]. An advantage of PDX leukemia over cell lines is that they consistently engraft at the BM site, recapitulating orthotopic disease [13, 15]. PDX models overcome several translational challenges of cell lines, e.g., alterations acquired by in vitro culture [9, 16–18].

To search for functionally relevant leukemia surface molecules in the small fraction of dormant LSC, we combined ultra-sensitive proteomics with CRISPR‒Cas9 reverse genetics in vivo screens. In mass spectrometry (MS)-based proteomics, the latest technological developments substantially increased sensitivity and completeness of the data by introducing a nearly lossless ultra-highly sensitive sample preparation workflow, parallel accumulation-serial fragmentation, data-independent acquisition, computational strategies and ultra-highly sensitive benchtop MS [19–21]. As a result, proteomics can now elucidate highly complex biological samples in great depth and at single-cell level, which has mostly been applied on cells in culture so far. Here, it allowed us to study the disease biology of a very rare cell type, namely dormant leukemia PDX cells, which can be isolated only at minute numbers of 2000–3000 cells from murine BM [6]. CRISPR‒Cas9 screens have been fundamental in identifying cancer vulnerabilities [22–24]. However, CRISPR screens have thus far been mostly used in cell lines in vitro, while technical challenges have prevented their application to PDX models and in vivo, with rare exceptions [25].

Among interesting candidates to mediate leukemia crosstalk with the microenvironment is the metalloproteinase ADAM10 (A disintegrin and metalloproteinase domain-containing protein 10), which mediates ectodomain shedding of transmembrane proteins to regulate basic biological processes such as cell adhesion, migration, receptor signaling and cell survival [26, 27]. ADAM10 is essential for early embryonic development and important in the hematopoietic system, which is largely attributed to the cleavage and activation of NOTCH1 by ADAM10 [28, 29]. Deregulated ADAM10 activity has been associated with multiple diseases, including Alzheimer’s disease and autoimmunity [27]. In cancer, ADAM10 mediates tumor progression, metastasis and therapy resistance in several tumors [27, 30–34]. Accordingly, ADAM10 inhibitors are under development and clinically tested [27, 35–37]. Although ADAM10 has been shown to be upregulated in a subset of hematologic malignancies and to drive T-ALL through oncogenic NOTCH1 signaling, its functional role in most subtypes of acute leukemia remains elusive [27, 33, 38].

Here, we performed ultra-sensitive deep proteomics on dormant leukemia cells to identify surface molecules relevant for the leukemia-niche interaction, which we then subjected to CRISPR‒Cas9 in vivo screens in two AL PDX models. We identified ADAM10 as an essential regulator of PDX maintenance in vivo and demonstrated that targeting ADAM10 sensitized AL cells to treatment.

## Results

To identify novel treatment options in B-cell precursor (BCP)-ALL, we searched for functionally relevant leukemia surface molecules and studied a rare dormant stem cell population that strongly relies on interaction with the BM niche. We recently showed that slow-cycling, label-retaining cells (LRC) within the PDX leukemia cell population mimic relapse-inducing cells in patients and show treatment resistance; when these cells were released from the niche, they regained the capacities to proliferate and to respond to treatment [6, 7].

### Ultra-sensitive proteomics reveals regulation of cell adhesion in slow-cycling PDX ALL cells

**Fig. 1:**
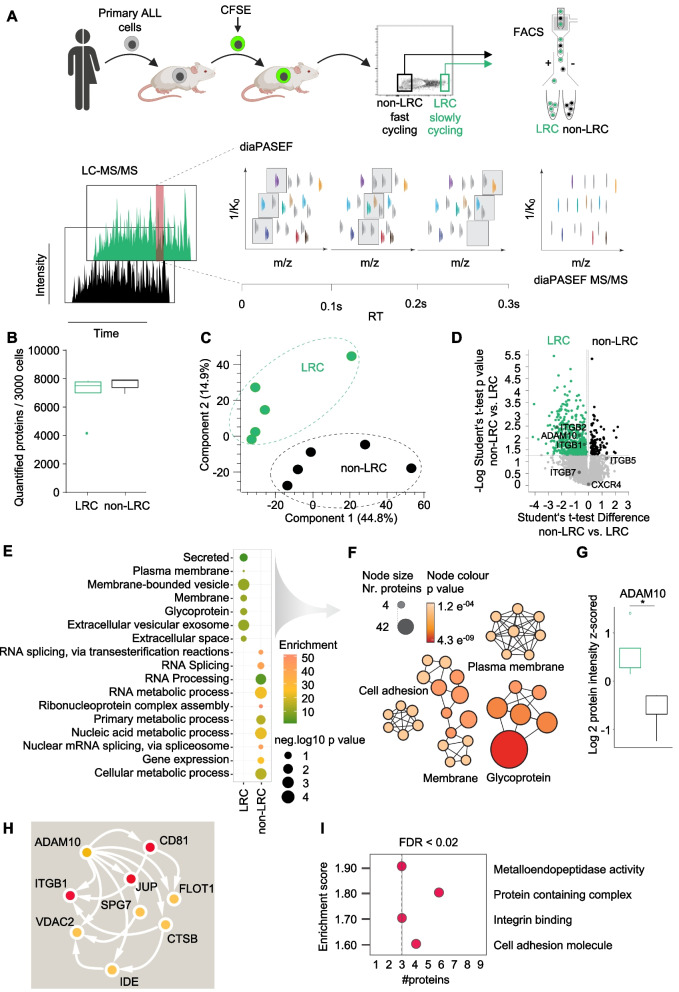
Ultra-sensitive proteomics reveals regulation of cell adhesion in slow-cycling PDX ALL cells. **A** Experimental design of LRC proteome by diaPASEF. PDX cells of ALL-199 and ALL-265 were stained with the division-sensitive dye CFSE and transplanted into mice (*n* = 5 per sample). After 14 days, re-isolated PDX cells were enriched by magnetic cell sorting and slow-cycling, label-retaining cells (LRC) and fast-cycling (non-LRC) cells were separated by flow cytometry according to their CFSE content. Following lysis and protease digestion, purified peptides were injected into a nanoflow liquid chromatography (LC) system coupled online to a high-resolution TIMS quadrupole time-of-flight mass spectrometer (timsTOF Pro) using diaPASEF acquisition mode. The scheme shows quadrupole isolation windows in two-dimensional 1/K_0_—m/z plane for diaPASEF acquisition with a 100 ms TIMS scan time and the diaPASEF MS/MS spectra correspond to the precursor ion selection. **B** Number of proteins quantified from a total of 3000 cells of LRC (*n* = 5) and non-LRC (*n* = 5) samples. Peptide and protein level FDR cut-off at 1%. **C** Appearance of LRCs and non-LRCs in principle component analysis (PCA). **D** Volcano plot displaying significantly regulated quantified proteins in LRC and non-LRC. **E** Box plot representation of ADAM10 which is significantly upregulated in LRC compared to non-LRC. Z-scored log2 protein intensity is displayed for proteins with permutation-based FDR cut-off < 0.05. **F** Gene Ontology (GO) enrichment analysis (Fisher’s exact test) of proteins significantly enriched in LRC and non-LRC. **G** GO network analysis of enriched pathways in LRC was built using the ClueGo app in Cytoscape. **H** String network map of ADAM10-interacting proteins from the LRC-regulated proteome. Nodes in red color indicate proteins with functional dependencies in leukemia cells (according to Depmap (https://depmap.org/portal/)). **I** Fisher’s exact test showing the enrichment of Cell adhesion and Metalloendopeptides terms from the ADAM10 network in LRCs

Because slow-cycling LRC are extremely rare in PDX leukemia models, flow cytometric enrichment allowed the isolation of only a few thousand cells per mouse. To be able to capture the proteomes from two well-characterized BCP-ALL PDX models, ALL-199 and ALL-265 (Table S1), we employed our data-independent acquisition method using a parallel accumulation–serial fragmentation (diaPASEF) MS workflow, which allows proteomic quantification to the single-cell level (Figs. 1A and S1A) [19, 20, 39]. We had confirmed the sensitivity of the diaPASEF MS workflow by measuring HeLa and SJSA1 cell lysates in a dilution series of peptide amounts of 10, 20 and 50 ng, equivalent to hundred to few hundred cells (Fig. S1). In as little as 10 ng of cell lysate equivalent to 100 cells, more than 7000 proteins could be detected, with exceptional data reproducibility between the replicates (Pearson correlation, *r* = 0.98), and samples clustered according to protein amount and cell type, suggesting the suitability of the approach (Fig. S1B-G, details in materials and methods).

When applied to PDX leukemia samples, a total of 8774 proteins were identified in both LRC and non-LRC, at a peptide and protein level FDR cut-off of 1%, and more than 7000 proteins in nine out of ten samples showed very high correlation (Pearson *r* = 0.92) between biological replicates (Figs. 1B and S1H). Of note, for one LRC replicate sample (LRC_1), only 1000 cells were available, from which ~ 4000 proteins could still be quantified (Figs. 1B and S1H). The dynamic range of protein signals spanned more than six orders of magnitude (Fig. S1I, J), and a good separation of LRC and non-LRC was observed by principal component analysis, independent of PDX model identity (Fig. 1C).

Several proteins were significantly upregulated in slow-cycling LRC compared to rapidly cycling non-LRC (Fig. 1D and Table S2), and slow-cycling LRC showed increased expression of plasma membrane, glycoprotein and extracellular cell adhesion proteins (Fig. 1E). Processes including RNA splicing and processing as well as metabolism were underrepresented (Fig. 1E), suggesting that LRC did not actively proliferate, which is in agreement with our previous functional findings and expression data indicating a metabolically dormant state in LRC [6]. Network analysis identified a substantially increased number of membrane proteins and glycoproteins in LRC, including ITGB1, ITGB2, ITGA4, ITGA7 and ADAM10 (Figs. 1F, G and S1K).

Collectively, these data show that membrane proteins are expressed at significantly higher levels in slow-cycling, therapy-resistant LRC than in their fast-cycling counterparts. Of note, the metalloproteinase ADAM10 is integrated in a network with other proteins that are exclusively regulated in LRC, among them CD81, ITGB1, JUP, FLOT1, SPG7, VDAC2, CTSB, and IDE, which are mainly associated with metalloendopeptidase activity and cell adhesion (Fig. 1H, I). Importantly, three out of nine of these proteins - CD81, JUP and ITGB1 – have a strong dependency in leukemia cells according to the DepMap dataset (https://depmap.org/portal/) providing evidence that our approach is suitable to identify candidate surface molecules with potentially important roles in leukemia.

### A pipeline for in vivo CRISPR‒Cas9 dropout screens in ALL PDX models

**Fig. 2:**
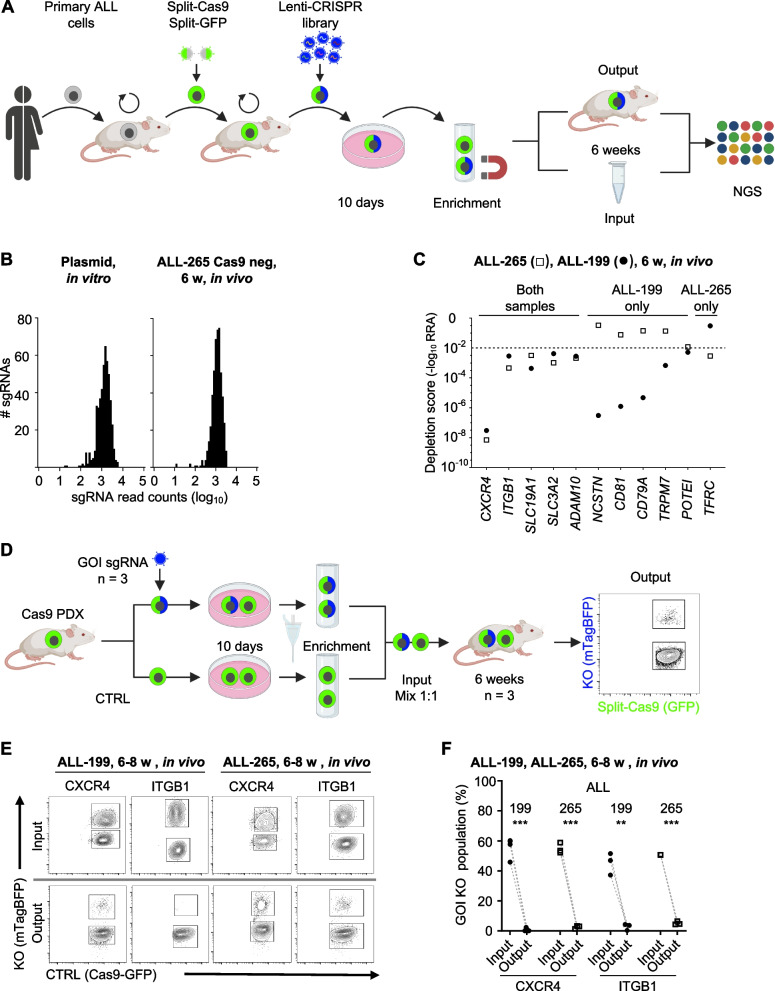
A pipeline for in vivo CRISPR-Cas9 dropout screens in ALL PDX models. **A** Workflow of CRISPR-Cas9 screening experiments. Primary leukemia cells from patients were transplanted into mice, re-isolated, and lentivirally transduced with split-Cas9 and a lenti-CRISPR library. Cells were cultured for 10 days in vitro, and MACS-enriched Cas9/sgRNA double-positive cells were transplanted into mice (*n* = 5 for ALL-199, *n* = 8 for ALL-265). PDX cells were re-isolated from mice with advanced leukemia and sgRNA distribution was analyzed by next generation sequencing (NGS) in comparison to the input control. **B** Distribution of all sgRNAs present in the library was analyzed by NGS in the plasmid pool and in re-isolated cells from split-Cas9-negative PDX samples (*n* = 2) after 6 weeks of in vivo growth. **C** Depletion score calculated using the MAGeCK robust ranking algorithm (RRA) for dropout genes in both samples or exclusively in ALL-199 or ALL-265. Dotted line represents cut-off at < 0.01. **D** Workflow of in vivo competitive validation assay. Split-Cas9-GFP-transgenic PDX cells were either used as control (CTRL, GFP-positive) or lentivirally transduced with sgRNAs targeting the gene of interest (GOI, mTagBFP-positive). Cells were cultured for 10 days, before sgRNA-positive cells were enriched by FACS. KO cells and CTRL cells were mixed in a 1:1 ratio and injected into three mice, one mouse per sgRNA. Animals were sacrificed at advanced leukemia and the distribution of the two re-isolated cell populations was evaluated by flow cytometry. **E** Representative flow cytometry plots of in vivo competitive validation assay for *CXCR4* and *ITGB1* in ALL-199 and ALL-265. Distribution of mTagBFP-positive KO cells and mTagBFP-negative CTRL cells in the injection mixture (1:1 ratio, Input, upper panel) and in re-isolated PDX cells after 6 weeks of in vivo growth (Output, lower panel) is shown. **F** Quantification of in vivo competitive validation assay for *CXCR4* and *ITGB1*. Percentage of the KO populations in the injection mixture and in corresponding re-isolated PDX cells of ALL-199 (*n* = 3 each GOI, each 3 animals with 3 BM measurements) and ALL-265 (*n* = 3 for CXCR4 and *n* = 3 for ITGB1 with 3 animals with the same ITGB1 sgRNA; for each GOI 3 animals with 3 BM measurements) are depicted. *** *p* < 0.001, ** *p* < 0.01 by paired t-test

Among the candidate surface and adhesion molecules identified, we aimed to decipher those with essential functions for leukemia. For this purpose, we generated genetically engineered PDX (GEPDX) models and established a comprehensive CRISPR‒Cas9 screening workflow, allowing for functional reverse genetics in PDX ALL models in vivo (Fig. 2A).

We utilized a split version of Cas9, which recombines via inteins to increase the efficacy of lentiviral transduction of difficult-to-transduce PDX cells [40]. Split-Cas9 was further concatamerised to a split version of GFP that gains functionality upon leucine-zipper-directed protein reassembly, according to bimolecular fluorescence complementation [41] (Fig. S2A). GFP expression was highly correlated with the expression of full-length Cas9 and was used as a marker to enrich successfully transduced and Cas9-recombined cells via flow cytometry (Fig. S2B, C). Lentiviral transduction resulted in PDX models stably expressing Cas9 over multiple passages in mice, which represents an attractive tool for a broad range of future studies (Fig. S2B).

For the sgRNA plasmid, we decided to enrich transduced cells either by fluorescence-based flow cytometry or magnetic beads (MACS); therefore, the sgRNA vector encodes an H-2Kk-mTagBFP fusion protein enabling the efficient preselection of transgenic cells before transplantation into mice (Fig. S3A). sgRNAs were designed using the Broad Institute sgRNA designer tool [42]. As quality control of the system, transduction of leukemia cell lines expressing split-Cas9 with sgRNAs targeting CD19, CD81 or F11R strongly decreased the expression of the respective cell surface proteins (Fig. S3B, C).

We next designed sgRNA libraries such that the entire library could be retrieved from each single mouse with high reproducibility, which required customized libraries instead of large genome-wide approaches. The maximum library size applicable in GEPDX AL models in vivo is sample-specific and determined by factors such as the frequency of leukemia-initiating cells (LIC) as stem cell surrogates and the number of cells engrafting after cell transplantation (Fig. S3D). To establish the workflow, ALL-199 and ALL-265, known for their high LIC frequencies, were selected. Aiming for at least 200 cells per sgRNA and five sgRNAs per target gene and calculating with generous safety margins for each step of the protocol, we decided to use a library size of approximately 100 target genes, including controls (Fig. S3D and Tables S3 and S4).

For in vivo screens (Fig. 2A), split-Cas9-transgenic GEPDX ALL cells were isolated from donor mice and transduced with the sgRNA library at low multiplicity of infection, aiming for a single genomic integration per cell. After ten days of ex vivo culture to allow gene editing and fluorochrome expression, transgenic cells were enriched by MACS for the co-expressed H-2Kk-mTagBFP fusion protein (Fig. S3E, F), transplanted into groups of mice or used as input control. When mice developed overt leukemia after several weeks of in vivo growth, PDX cells were re-isolated, and sgRNA distribution was analyzed using a nested PCR from genomic DNA (Fig. S4 and Table S5). Deep sequencing was performed at a minimum coverage of 500 reads per sgRNA, followed by data analysis using the MAGeCK algorithm [43].

Thus, by optimizing various steps, we established a ready to use workflow that allows customized CRISPR‒Cas9 dropout screens in leukemia PDX models in vivo.

### A CRISPR‒Cas9 dropout screen for leukemia surface proteins

The sgRNA library addressed surface molecules that were selected from our own multi-omics data (Fig. 1 and [6]), complemented by candidates from the literature (Table S3). Five hundred sgRNAs were designed, including non-cutting sgRNAs as negative controls and known essential genes as positive controls (Table S4). The sgRNAs were cloned into the lentiviral sgRNA vector (Fig. S3A) and a uniform abundance of all sgRNAs with a distribution of less than 1.5 orders of magnitude was confirmed by deep sequencing of the plasmid pool (Fig. 2B).

To quality control the suitability of the library size for each PDX model, in vivo experiments were performed in the absence of Cas9 expression so that sgRNAs served as barcodes. The sgRNA distribution remained unchanged between input and output in replicate mice, indicating that the entire sgRNA library could be recovered from each mouse in both ALL-199 and ALL-265 (Figs. 2B and S5A). In the verum screen using split-Cas9-transgenic GEPDX cells, sgRNA distribution revealed a high correlation between replicate mice (Figs. S5B, C) and similar low Gini indices (Tables S6 and S7), indicating that the library size was suitable for both models.

For the verum screens and upon Cas9-mediated KO in ALL-199 and ALL-265, a subset of sgRNAs was significantly depleted in the output samples compared to the input samples (Fig. S6). Dropout analysis using the MAGeCK algorithm [43] identified eleven and six significantly depleted genes in ALL-199 and ALL-265, respectively (Figs. 2C and S6). Of these, five genes were commonly depleted in both models, namely *CXCR4*, *SLC3A2*, *SLC19A1* as well as *ITGB1* and *ADAM10*, which were enriched in the proteome analysis of LRC (Fig. 1). Intriguingly, each PDX model additionally revealed unique dropout genes, such as *CD79A* in ALL-199 or *TFRC* in ALL-265 (Figs. 2C and S6), suggesting a capacity of the screening approach to detect patient sample-specific dependencies.

### Molecular target validation in PDX models in vivo

We next established an assay to individually validate dropout candidates in PDX models in vivo. Competitive in vivo approaches were frequently used in mouse studies on normal hematopoiesis to spare resources and benefit from high sensitivity and reliability; previously, we have adapted them for their use in PDX models [44–47]. PDX cells expressing gene of interest (GOI) sgRNA sequences were mixed at a defined 1:1 ratio with control cells. As control (CTRL) cells either cells without sgRNA transduction were used (Figs. 2D and S7A) or cells transduced with a second sgRNA vector, where mTagBFP was replaced by T-Sapphire to distinguish the different cell populations (Fig. S7B, C). For each target gene, three independent, quality-controlled sgRNAs were cloned and transduced into PDX cells (Fig. S7). After a few days of ex vivo culture, equal numbers of CTRL and GOI KO sgRNA cells were mixed and injected into three replicate mice, each then harboring two different cell populations (CTRL and GOI KO). When mice developed overt leukemia, the relative distribution of both populations was determined by flow cytometry.

We first validated *CXCR4* and *ITGB1* which are well-known to influence the leukemia-niche interaction and dropped out in both ALL samples (Fig. 2C, E) [48, 49]. Evaluation of genomic indel frequencies indicated high editing efficiency for all sgRNAs targeting *CXCR4* or *ITGB1*, resulting in a marked decrease in the expression of the respective surface protein (Fig. S8A-C). Both *CXCR4* and *ITGB1* KO populations were significantly decreased in both PDX models in competitive in vivo experiments, confirming the screening results and reproducing the known essential roles of both proteins in our ALL PDX models in vivo (Figs. 2E, F and S8D). Overall, our pipeline of customized sgRNA library screens in PDX models was able to identify targets with essential in vivo function.

### ADAM10 is essential for PDX acute leukemia in vivo

The screen revealed dropout candidates with yet undefined roles in B-ALL. We focused on *ADAM10* because it was found to be significantly upregulated in LRC (Fig. 1G) and is known to play an important role in the progression of T-ALL and cancers of multiple entities, as well as in normal hematopoiesis [26–37].

**Fig. 3:**
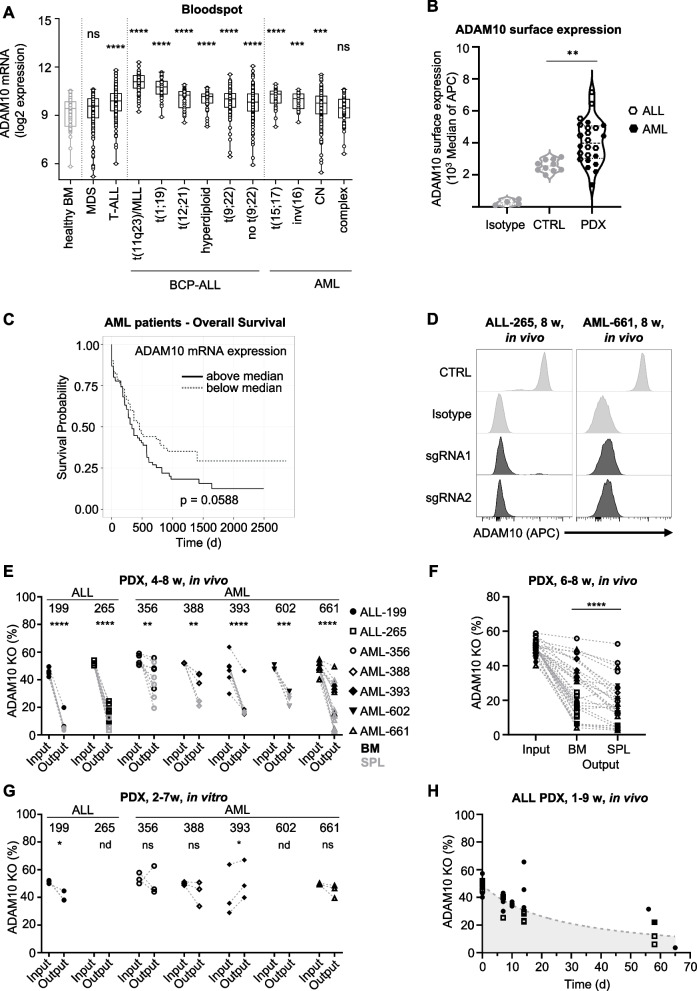
ADAM10 is essential for PDX acute leukemia in vivo. **A** Publicly available data from the BloodSpot databank were analyzed for ADAM10 mRNA expression profile in leukemia samples with the indicated BCP-ALL and AML subtypes compared to healthy donor BM controls (dataset 202603_at; Leukemia MILE study GSE13159). Box indicates median, 25^th^ and 75^th^ percentile; whiskers indicate min/max. **** *p* < 0.0001, *** *p* < 0.001 by multiple t-test compared to healthy BM group. ns: not significant, CN: cytogenetically normal. **B** Flow cytometry analysis of ADAM10 surface expression in ALL (*n* = 14) and AML (*n* = 10) PDX samples compared to healthy donor BM (*n* = 10) samples. Isotype control of representative PDX and BM samples are included for comparison. **C** Correlation of ADAM10 expression in AML blasts with overall survival in 172 AML patients (dataset for 202603_at; Human AML cells GSE13159). **D**-**F** In vivo competitive ADAM10 validation in AML and ALL PDX samples. **D** ADAM10 surface protein expression in control (CTRL and Isotype) and KO ALL and AML PDX samples transduced with the indicated sgRNAs and re-isolated following 8 weeks of in vivo growth. Histograms of cells transduced with two independent ADAM10-sgRNAs are shown. **E** Quantification of in vivo competitive validation assay. Percentage of the ADAM10 KO population in the injection mix (Input) and in the corresponding re-isolated PDX from BM (black) or spleen (grey) at advanced leukemia (Output) for ALL-199 (*n* = 7; 4 animals with 4 BM and 3 spleen measurements), ALL-265 (*n* = 12; 9 animals with 9 BM and 3 spleen measurements), AML-356 (*n* = 9; 5 animals with 4 BM and 5 spleen measurements), AML-388 (*n* = 6, 3 animals with 3 BM and 3 spleen measurements), AML-393 (*n* = 9; 6 animals with 6 BM and 3 spleen measurements), AML-602 (*n* = 6; 3 animals with 3 BM and 3 spleen measurements) and AML-661 (*n* = 14; 7 animals with 7 BM and 7 spleen measurements). **** *p* < 0.0001, *** *p* < 0.001, ** *p* < 0.01 by paired t-test. **F** Dropout of ADAM10 KO cells is more prominent in spleen compared to BM in some samples. Percentages of ADAM10 KO cells in the BM and spleen derived from E. **** *p* < 0.0001 by paired t-test. **G** Quantification of in vitro competitive validation assay for ADAM10. Percentage of the ADAM10 KO population before (Input) and after the in vitro cultivation period (Output) (all PDX samples *n* = 3, 3 individual sgRNAs). *p < 0.05 by paired t-test, nd (not determined), ns (not significant). **H** In vivo competitive validation assays for ADAM10 in ALL-199 and ALL-265 after the indicated in vivo growth times. Percentage of the KO populations in the injection mixture and in PDX cells re-isolated from the BM is depicted. Grey dotted line is the interpolation of the data using Pade (1,1) approximant, robust fit

The ADAM10 mRNA expression level was significantly elevated in various AL subtypes compared to healthy donor BM in publicly available datasets (Fig. 3A), supporting a putative role of ADAM10 in both ALL and AML. Similarly, our cohort of 25 AL PDX models across all molecular subtypes showed increased ADAM10 protein expression compared to BM samples from healthy controls (Fig. 3B and Table S1). The ADAM10 protein was also abundantly expressed in B-ALL and AML cell lines (Fig. S9A), and its expression was significantly increased in our previously published proteome of AML patient samples compared to healthy donor controls (Fig. S9B) [50]. Of clinical relevance, high ADAM10 expression in samples from the TCGA dataset was associated with a trend toward worse overall survival in AML patients (Fig. 3C). In patients with lung or pancreatic cancer, ADAM10 expression was significantly associated with overall survival (Fig. S9C).

Thus, we studied whether ADAM10 is functionally relevant for BCP-ALL. sgRNAs targeting ADAM10 induced full KO at the protein level (Fig. 3D) and substantially impaired PDX leukemia growth in mice in two out of three BCP-ALL PDX models (Figs. 3E, F and S10A-C). Because elevated ADAM10 expression was not restricted to ALL samples, we also analyzed AML PDX models. All five AML PDX models studied showed dependency on ADAM10, albeit to varying degrees and with a stronger phenotype in spleen compared to BM in some samples (Figs. 3E, F and S10A, B). Overall, dependency on ADAM10 was demonstrated in seven out of eight AL PDX models in vivo, suggesting that ADAM10 plays an essential role in both types of acute leukemia, ALL and AML, independent of the molecular subtype or genetic abnormalities (Table S1). In vitro, the anti-proliferative function of ADAM10 inhibition was measurable in PDX ALL-199, but not in several PDX AML models incubated for prolonged periods of time (Fig. 3G). Of note, in ALL-199 and ALL-265, the number of ADAM10 KO cells in the BM was already reduced in the early stage of leukemia and decreased further during steady-state leukemia, indicating that ADAM10 KO affected both, cell homing and proliferation (Fig. 3H).

### Molecular reconstitution confirms essentiality of ADAM10 in PDX ALL in vivo

To unequivocally confirm that ADAM10 plays an essential role in PDX leukemia cells, competitive in vivo reconstitution assays were performed in ADAM10 KO cells. As a technical challenge, these assays require studying PDX models in vivo, where the tumor-niche interaction is most faithfully modeled [13, 15].

**Fig. 4:**
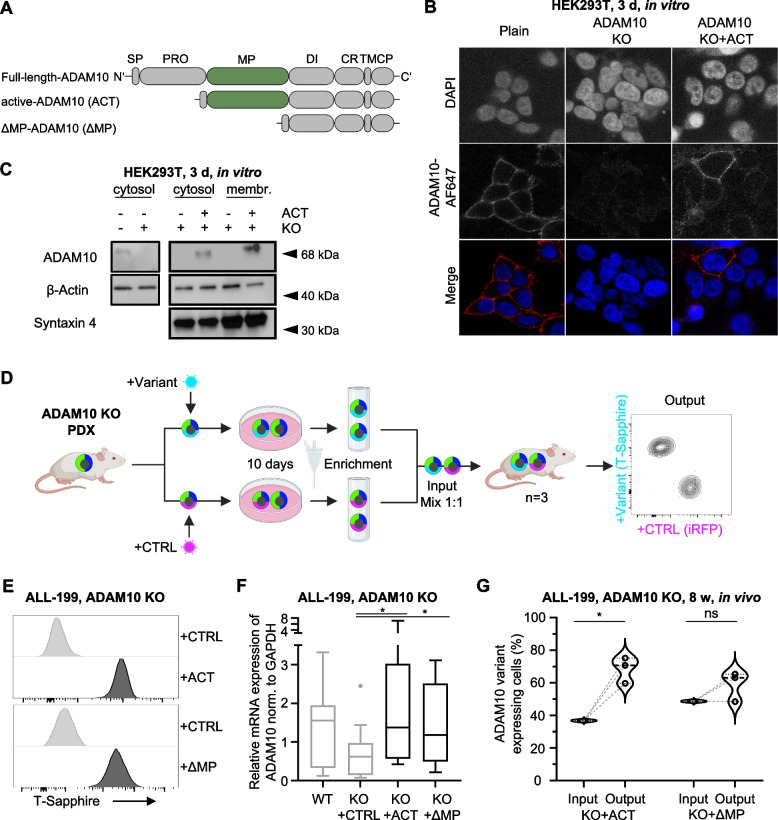
Molecular reconstitution confirms essentiality of ADAM10 in PDX ALL in vivo. **A** Schematic illustration of different ADAM10 expression variants. Upper construct: full-length ADAM10 protein, middle panel: constitutive catalytically active ADAM10 variant (ACT), lower panel: enzymatically inactive ADAM10 variant, lacking the metalloproteinase domain (ΔMP); SP: signal peptide, PRO: prodomain, DI: disintegrin domain, CR: cysteine-rich domain, TM: transmembrane domain, CP: cytoplasmic domain. **B** Confocal microscopy pictures of expression of recombinant ADAM10. ADAM10 was stained (Alexa Fluor (AF) 647) on fixed un-permeabilized HEK293T control (Plain), ADAM10 KO cells or ADAM10 KO cells with re-expression of the active ADAM10 construct (ADAM10 KO + ACT). DAPI was used for nuclear staining. Representative images of three independent experiments are shown. **C** Immunoblot of ADAM10 expression in crude cell lysates (cytosol) and membrane fraction (membr.) in HEK293T wildtype or ADAM10 KO cells with or without expression of constitutively active-ADAM10 (ACT). Syntaxin 4 and β-Actin were used as membrane protein marker and loading control, respectively. **D** Workflow of in vivo competitive ADAM10 reconstitution assays. **E** Representative flow cytometry analysis of T-Sapphire expression. T-Sapphire expression levels were compared between the ADAM10 KO PDX cells transduced with the iRFP mock vector (CTRL) and the active-ADAM10 (ACT)- or ΔMP-ADAM10 (ΔMP)-expressing cells after isolation from the animals. One representative histogram out of three individual animals per group is shown. **F** ADAM10 mRNA expression in parental split-Cas9-positive ALL-199 (CTRL), ADAM10 KO ALL-199 cells transduced with the iRFP mock vector (KO + CTRL) and ADAM10 KO reconstituted with the ACT or ΔMP variants was determined by qRT-PCR and normalized to GAPDH. Box indicates median, 25^th^ and 75^th^ percentile; whiskers indicate 25^th^ percentile - 1.5 IQR (inter-quartile distance) and 75^th^ percentile + 1.5 IQR. * *p* < 0.05 by unpaired t-test. **G** Distribution of subpopulations of the competitive in vivo ADAM10 reconstitution assay. Percentages of cells expressing the indicated ADAM10 variant from the injection mixture (Input) are compared to PDX cells isolated from murine BM (Output) after a similar in vivo growth period of 8–9 weeks. Violin plot with median indicated by dashed line and 25^th^ and 75^th^ percentile by dotted lines. Data of three animals is shown. ns not significant, * *p* < 0.05 by paired t-test

ADAM10 is activated by proteolytic cleavage of its prodomain via convertase proteins. To express a catalytically active ADAM10 protein, a variant lacking the prodomain (ACT) was generated. In addition, a truncated form also devoid of the metalloproteinase domain (ΔMP) was cloned, which allowed studying the functional relevance of the MP domain (Fig. 4A) [26]. Membrane localization of recombinant active ADAM10-ACT was confirmed in HEK293T cells with ADAM10 KO by confocal microscopy (Figs. 4B and S11) and in protein lysates of the membrane fraction (Fig. 4C).

For competitive reconstitution in vivo assays, PDX ALL-199 ADAM10 KO cells were reconstituted with either ADAM10-ACT (+ ACT) or ADAM10-ΔMP (+ ∆MP), both linked to T-Sapphire, mixed with iRFP-expressing ADAM10 KO control (CTRL) cells and injected into mice (Fig. 4D). When mice displayed signs of overt leukemia, cells were re-isolated, and subpopulation distribution was analyzed (Fig. 4D). Re-expression of either ADAM10-ACT or ADAM10-ΔMP was indicated by the expression of the fluorochrome T-Sapphire (Fig. 4E) and confirmed on the mRNA level, where both variants were re-expressed close to ADAM10 levels in wildtype cells (Fig. 4F).

Re-expression of ADAM10-ACT rescued the growth disadvantage conferred by ADAM10 KO in ALL-199 PDX cells (Fig. 4G), excluding putative off-target effects and confirming that ADAM10 activity is essential for in vivo growth. In contrast, reconstitution of ADAM10 KO cells with the ADAM10-ΔMP variant had insignificant effects on the growth of ADAM10 KO cells (Fig. 4G). These data unequivocally confirm the essential function of ADAM10 in PDX ALL cells in vivo, which is, at least in part, mediated by its enzymatic metalloproteinase activity.

### Downstream effects of ADAM10 KO

**Fig. 5:**
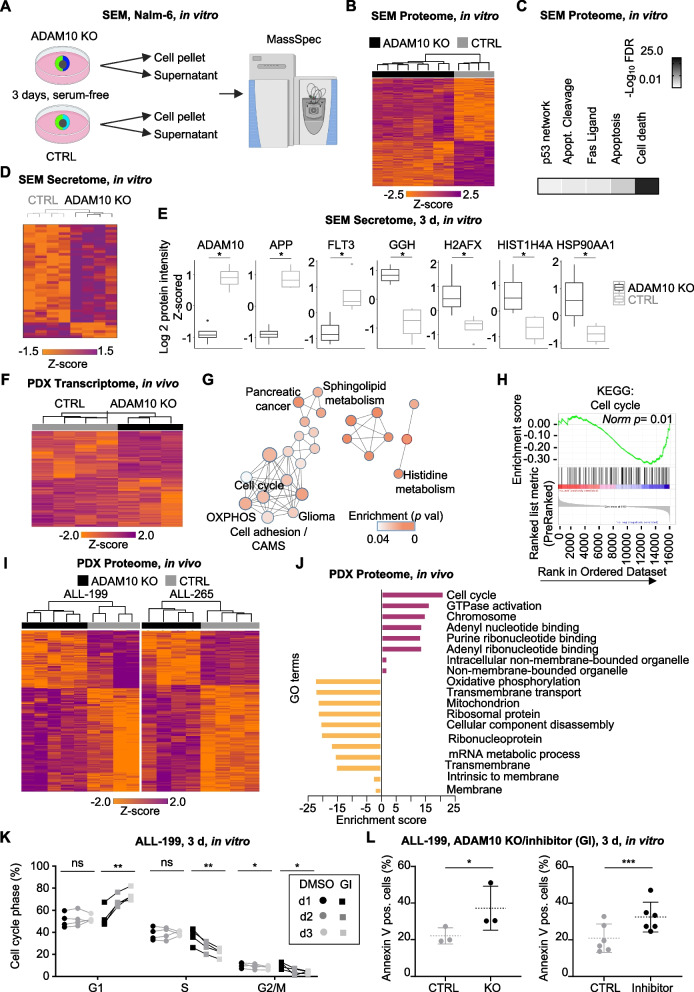
Molecular profiling and functional analysis reveal a role of ADAM10 in cell cycle progression and apoptosis. **A**-**E** Proteome and secretome analysis of ADAM10 KO and control SEM and Nalm-6 cells. **A** Workflow of mass spectrometry-based secretome and proteome analysis of ADAM10 KO and CTRL cells. **B** SEM proteome. Heat map of unsupervised hierarchical clustering of significantly regulated proteins of control (*n* = 4) vs. ADAM10 KO (*n* = 8) in SEM cells (two-sample test, permutation-based FDR < 0.05). **C** Pathway enrichment results of the proteome analysis described in **B**. The five most significantly altered pathways are depicted. FDR: false discovery rate. **D** SEM secretome. Heat map of unsupervised hierarchical clustering of significantly regulated secreted proteome of control (*n* = 4) vs. ADAM10 KO (*n* = 4) in SEM cells (two-sample test, permutation-based FDR < 0.05). **E** Box plots showing significantly regulated secreted proteins in SEM cells with ADAM10 KO. Plot displays z-scored log 2 protein intensity of selected proteins. **F** Transcriptome analysis of ADAM10 KO (*n* = 3) and CTRL PDX (*n* = 4) samples of ALL-199 and ALL-265. Heatmap of genes differentially expressed between ADAM10 KO and CTRL cells with unadjusted *p* value of ≤ 0.05 and fold change < 0.5 or > 2 is shown. **G** Pathway enrichment results of transcriptome analyses described in **F** were mapped into a network of gene sets (nodes) related by gene overlap (lines). Node size is proportional to the number of genes in each set and the enrichment significance (FDR *p* value) is represented as a node color gradient. Proportion of shared genes between gene sets is depicted as the thickness of the blue line surrounding the nodes. The major functional groups are annotated. Data analyzed and visualized by GSEA 4.1.0 and Cytoscape 3.9.0. **H** Gene set enrichment analysis (GSEA) for the KEGG term cell cycle (*p* < 0.005 and FDR *q* value < 0.33, Norm *p* = 0.01). **I** Proteome of PDX cells. Heatmap of significantly regulated proteins by unsupervised hierarchical clustering of ALL PDX sample with control sgRNA (ALL-199 *n* = 4, ALL-265 *n* = 4) vs. ADAM10 KO sgRNA (ALL-199 *n* = 5, ALL-265 *n* = 4) (two-sample test, permutation-based FDR < 0.05). **J** Plot displaying the enriched and de-enriched GO term categories (for panel **I**) upon ADAM10 KO in ALL PDX samples by Fisher’s exact test. **K** Cell cycle analysis of ADAM10 inhibitor (GI254023X, 490 µM)- or DMSO-treated ALL-199 PDX cells. Percentage of cells in the indicated cell cycle phase was quantified on day 1, day 2 and day 3 of treatment with the ADAM10 inhibitor or DMSO. Each dot represents the mean of four replicates. G1 = Gap phase 1, S = Synthesis phase, G2/M = Gap phase 2/mitosis. ** *p* < 0.01, * *p* < 0.05 by paired t-test. **L** Apoptosis assay in ALL-199 PDX cells with ADAM10 KO (*n* = 3) or treated with ADAM10 inhibitor (GI254023X, 490 µM, *n* = 6). *** *p* < 0.001, * *p* < 0.05 by paired t-test

To gain insights into the downstream effects of ADAM10 in leukemia, we performed proteome and transcriptome profiling of AL cells with or without ADAM10 expression. In the SEM leukemia cell line, 854 proteins were down- and 1120 proteins upregulated upon ADAM10 KO, showing an activation of processes such as apoptosis/cell death, cell cycle, metabolism and membrane/adhesion upon KO (Figs. 5A-C, S12 and Table S8).

Because ADAM10 mediates ectodomain cleavage of transmembrane proteins via its sheddase function, we investigated the composition of cleaved proteins in the supernatant by collecting the secretome of ADAM10 KO versus control cell line cells (Figs. 5A, D, E and S13A, B). While more than 220 proteins were quantified in the secretome of SEM and nearly 1000 proteins in the supernatant of Nalm-6 cells, 44 and 62 proteins were differentially secreted, respectively, including ADAM10 itself (Figs. 5D, E, S13C, D and Table S9). Among these, five proteins were commonly affected in the secretome of both cell lines, including the well-known ADAM10 target amyloid-beta precursor protein (APP) (Figs. 5D, E and S13C). Of note, cancer-relevant proteins, such as fms-like tyrosine kinase 3 (FLT3) and heat shock protein 90 α (family class A member 1, HSP90AA1, HSP90α), were differentially secreted upon ADAM10 KO, indicating that they may represent candidates mediating the pro-tumorigenic function of ADAM10 in leukemia (Figs. 5D, E, S13 and Table S9).

We further analyzed the transcriptome and proteome signatures of PDX ALL-199 and ALL-265 cells with or without ADAM10 KO (Figs. 5F-J, S14A). Transcriptome analysis revealed 641 differentially expressed genes – 314 upregulated and 327 downregulated – in ADAM10 KO cells (Fig. 5F). Enrichment map analysis revealed that ADAM10 KO modulated biological processes such as cell cycle, oxidative phosphorylation (OXPHOS), and cell adhesion (Fig. 5G, H and Table S10). Gene Set Enrichment analyses (GSEA) showed alterations in several Kyoto Encyclopedia of Genes and Genomes (KEGG) terms, including cell cycle, OXPHOS and cell adhesion (Figs. 5H and S14A).

The proteome analysis of ALL PDX models with or without ADAM10 KO identified several hundred proteins deregulated compared to controls (Figs. 5I, S14B and Table S11). Enrichment analysis revealed cell cycle, OXPHOS, mitochondrion and cell adhesion as major deregulated cellular components, processes and pathways (Figs. 5J and S14C). Interestingly, cell cycle, metabolism and apoptotic processes were found to be deregulated by ADAM10 KO in both the transcriptome and proteome in both cell line cells and PDX cells and in both ex vivo and in vivo experimental models, suggesting that the leukemia-promoting function of ADAM10 is, at least in part, independent from the tumor-niche interaction between leukemia cells and the BM.

### ADAM10 inhibition reduces leukemia cell fitness in vitro

To functionally validate certain KO-associated alterations, we first set out to analyze effects of ADAM10 depletion on cell proliferation, cell death and colony formation in vitro. For translational purposes, we took advantage of the long-standing and current interest to pharmacologically target ADAM10 and utilized the ADAM10 inhibitor GI254023X [27, 34, 36, 37, 51]. When PDX cells were treated with the ADAM10 inhibitor GI254023X ex vivo at doses of 100 µM or higher, ADAM10 surface expression was strongly decreased, which is in line with published results (Fig. S15A) [52]. GI254023X treatment induced cell cycle arrest, with an increased proportion of cells in G1 and a decreased proportion in S phase (Figs. 5K and S15B). At higher doses (490 µM), pharmacological inhibition of ADAM10 by GI254023X increased the percentage of apoptotic and dead cells in both ALL PDX samples tested to a similar degree as observed in ADAM10 KO cells (Figs. 5L and S15C, D).

Taken together, the results of -omics profiling and functional tests revealed that genetic ablation or pharmacological inhibition of ADAM10 impaired leukemia cell cycle progression and survival.

### Targeting ADAM10 inhibits homing, reduces LSC frequency and sensitizes cells to chemotherapy in vivo

Finally, we aimed to obtain more detailed preclinical insights into whether targeting ADAM10 may be of potential clinical benefit for treating leukemia patients.

**Fig. 6:**
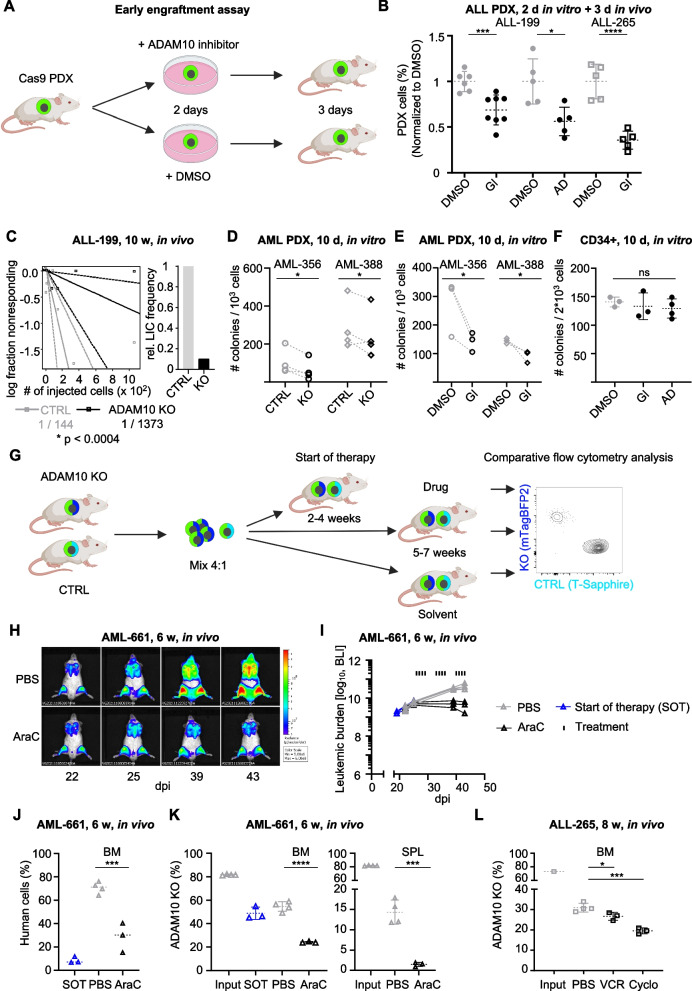
Targeting ADAM10 inhibits homing, reduces LSC frequency and sensitizes to chemotherapy in vivo. **A** Workflow for early engraftment assay. **B** Quantification of PDX cells homing to the BM. Number of DMSO- (GI group: *n* = 6, Aderbasib group: *n* = 5) or inhibitor- (GI: *n* = 8, Aderbasib 10 µM: *n* = 5) treated ALL-199 or DMSO- (*n* = 5) or inhibitor- (*n* = 5) treated ALL-265 were analyzed. Data were normalized to the mean of the respective DMSO group. Each dot represents one mouse. **** *p* < 0.0001, *** *p* < 0.001, *p < 0.05 by paired t-test. **C** Quantification of the limiting dilution transplantation assay. Mean (solid lines) and 95% confidence interval (CI, dashed line) are depicted (ALL-199 *n* = 25). Bar graph depicts relative LIC frequency of ADAM10 KO cells normalized to control. **D** Quantification of colony-forming unit assay with PDX AML-356 or AML-388 cells with or without ADAM10 KO. Each dot represents one replicate. *p < 0.05 by paired t-test. **E** Quantification of colony-forming unit assay with PDX AML-356 or AML-388 cells treated with the ADAM10 inhibitor GI254023X (GI, 100 µM) or DMSO for 72 h. Each dot represents one replicate. * *p* < 0.05 by paired t-test. **F** Quantification of colony-forming unit assay with healthy human CD34 + blood progenitor cells treated with the ADAM10 inhibitor GI254023X (GI, 100 µM), Aderbasib (AD, 10 µM) or DMSO for 72 h. Each dot represents one replicate. ns by Holm-Sidak’s multiple comparisons test vs. DMSO. **G** Workflow of the competitive in vivo chemotherapy trial. ADAM10 KO cells marked with mTagBFP or CTRL cells marked with T-Sapphire were injected into groups of mice in a 4:1 ratio of ADAM10 KO:CTRL cells to compensate for the disadvantage of ADAM10 KO cells. Tumor growth was monitored by repetitive bioluminescence in vivo imaging. Mice were sacrificed at start of therapy (SOT) or following treatment with chemotherapy or PBS. Percentages of the KO and CTRL populations among the isolated human cells (ADAM10 KO + CTRL cells) were determined by flow cytometry. **H** Representative in vivo bioluminescence imaging pictures of mice carrying AML-661 PDX cells treated with cytarabine (AraC, *n*=3, loss of 2 mice due to drug-related toxicities) or PBS (*n*=4, 1 mouse was removed as extreme value) at the indicated time points. **I** Quantification of all images taken from mice carrying AML-661 PDX cells over time. **J** Quantification of human cells in the BM in AML-661. *** *p* < 0.001, by unpaired t-test. **K** Quantification of distribution of ADAM10 KO PDX cells in AML-661 at injection, start of therapy (SOT) and after treatment with PBS or AraC. **** *p* < 0.0001, *** *p* < 0.001 by unpaired t-test. **L** In vivo chemotherapy trial with ALL-265 treated with vincristine (VCR, *n* = 3), Cyclophosphamide (Cyclo, *n* = 3) or PBS (i.p., *n* = 4). Quantification of distribution of ADAM10 KO PDX cells in ALL-265 at injection and after treatment with PBS, VCR or Cyclo. *** *p* < 0.001, * *p* < 0.05 by unpaired t-test

To understand whether ADAM10 is, beyond its cell-intrinsic effects, required for interaction with the in vivo microenvironment, the ability of newly transplanted PDX ALL cells to migrate and home to the murine BM was determined. For this assay, injection of a high number of tumor cells is necessary, because homing in mice is a highly inefficient process and only minute numbers of cells can be retrieved from the murine BM shortly after cell transplantation [6, 7]. ALL-199 and ALL-265 PDX cells were pre-treated with solvent or the ADAM10 inhibitors GI254023X or Aderbasib for two days before they were injected into mice (Fig. 6A). At these concentrations, the inhibitors reduced ADAM10 expression in PDX cells (Figs. S15A and S16A) but had no effect on cell viability in vitro (Fig. S16B, C). When BM was analyzed three days later, pharmacological inhibition of ADAM10 resulted in a clear reduction in leukemic cells homing into the BM. For example, GI254023X reduced homing of ALL-265 PDX cells by 65% (Fig. 6B). These data prove a functional role of ADAM10 for ALL cell homing to the in vivo BM environment and a combinatorial role of ADAM10 for leukemia cell-intrinsic and -extrinsic effects.

Because LSC represent the most important targets for anti-leukemia treatment due to their unique ability to induce disease relapse, we next determined the effect of ADAM10 KO on stem cells. We quantified LSC frequencies using limiting dilution transplantation assays (LDTA) as the gold standard approach, in a competitive setting according to the literature [45, 47]. GEPDX ALL-199 and ALL-265 cells with and without ADAM10 KO were mixed at a 1:1 ratio and injected into groups of mice in serial dilutions, and tumor engraftment was analyzed after eight weeks by flow cytometry. Loss of ADAM10 reduced LSC frequencies in both PDX models (Figs. 6C and S16D, E and Table S12), indicating that LSC depend on ADAM10 to induce leukemia. In line with our previous results and with -omics data indicating changed proliferation upon ADAM10 KO (Fig. 5), colony formation of AML PDX models in vitro was reduced in ADAM10 KO cells and in GI254023X-treated cells (Figs. 6D, E and S17). Importantly, colony formation capability of human CD34 + progenitor cells was not significantly altered by treatment with GI254023X or Aderbasib in anti-leukemic concentrations indicating that healthy cells of the hematopoietic system are less sensitive, allowing a therapeutic window (Figs. 6F and S17G). Taken together, reduced colony formation of AML ADAM10 KO PDX cells and reduced LSC frequencies in ALL PDX models in vivo suggest that targeting ADAM10 may represent a suitable approach to reduce LSC numbers.

Given the important role of both the in vivo microenvironment and LSC in mediating drug responses, we asked whether targeting ADAM10 may sensitize leukemia cells toward routine chemotherapy used to treat ALL or AML patients.

PDX cells with and without ADAM10 KO were mixed in a 4:1 ratio before injection into mice (Fig. 6G). This competitive approach ensured identical treatment conditions for both populations. PDX ALL-265 models were treated with cyclophosphamide, and PDX AML-661 were treated with cytarabine (AraC) for three consecutive weeks, while control animals received solvents. Leukemia growth and treatment response were monitored by repetitive bioluminescence in vivo imaging and demonstrated a moderate antitumor effect of AraC in AML-661 cells (Figs. 6H, I and S18A), in line with a reduced PDX cell count in the murine BM at the end of the experiment (Fig. 6J). When ADAM10 KO and CTRL cells were quantified by flow cytometry, the ADAM10 KO cells were significantly more reduced by AraC than CTRL cells in both organs, especially in the spleen (Figs. 6K and S18B-D). Similar effects were detected in PDX ALL-265 cells treated with cyclophosphamide or vincristine (Fig. 6L). Moreover, in in vitro chemotherapy trials treatment of ADAM10 KO PDX cells from AML-661 and AML-356 with clinically relevant concentrations of Cytarabine, Doxorubicine and Daunorubicine, resulted in significant, dose-dependent reduction of ADAM10 KO cells compared to CTRL cells (Fig. S19). These data indicate that loss of ADAM10 sensitized both ALL and AML PDX models to chemotherapy in vivo as well as AML PDX in vitro.

We conclude that drugs inhibiting ADAM10 may have the potential to impair the leukemia-niche interaction and tumor maintenance in patients with leukemia, thereby reducing tumor burden, diminishing stem cells and increasing the effectiveness of conventional chemotherapy against leukemia.

## Discussion

The rare subpopulation of dormant LSC represents the major reason for leukemia reoccurrence, resistance to therapy and poor prognosis. Combining highly sensitive quantitative proteomics to uncover unique features of dormant LSC with in vivo CRISPR screens to prove gene essentiality in PDX models, we identified the surface molecule ADAM10 as a critical molecule for LSC maintenance. ADAM10 is upregulated on dormant ALL cells and associated with poor clinical outcome; it is essential for AL homing to the BM, tumor growth, LSC maintenance and response to chemotherapy.

Until recently, deep proteome analysis of limited cell numbers or extremely rare cell populations was unfeasible because it demanded large sample amounts, extensive prefractionation and long measurement times. Here, we used a highly sensitive sample preparation workflow and diaPASEF technology, enabling the quantification of over 8500 proteins in just a few thousand dormant PDX LSC isolated from the in vivo environment, although leukemia cells are known for their low protein content [19–21, 50]. Using this improved technique, we identified ADAM10 as upregulated in the rare subpopulation of in vivo dormant PDX LSC.

Despite their major preclinical value, PDX models have largely been spared from molecular studies due to technical challenges [8, 12, 25, 53]. To further expand on the value of PDX models, we established (i) several AL PDX models stably expressing Cas9, (ii) CRISPR‒Cas9 dropout screening in vivo and (iii) a workflow for negative selection of genes to identify in vivo specific cancer dependencies at intermediate throughput. These models and protocols can now be used to gain clinically relevant insights for drug development and the process of prioritizing and selecting therapeutic targets.

Importantly, our screens identified both common and unique essentialities in patient samples. Among common genes were the well-known niche modulators *CXCR4* and *ITGB1* [54–57], demonstrating that the screening pipeline is suitable for identifying genes essential for the interaction with the in vivo environment. Sample-specific dropouts highlight the power of unbiased reverse genetic screening to identify patient-specific therapeutic targets and advance personalized medicine.

We identified *ADAM10* as essential for acute leukemias in vivo. According to the literature, ADAM10 acts through both tumor cell intrinsic effects and bidirectional crosstalk of PDX AL models with the tumor microenvironment (Figs. 3 and 6) [27, 32–34]. Surprisingly, while our in vivo data indicate that high ADAM10 expression is associated with dormancy in slow-cycling LRC (Fig. 1G), our in vitro data suggest that lack of ADAM10 promotes cell cycle arrest (Fig. 5K). This might be explained by different functions of ADAM10 in vivo and ex vivo*. *In vivo, the pro-niche function of ADAM10 might exceed its pro-proliferative function, and cell-intrinsic functions of ADAM10 including regulation of cell cycle and apoptosis might thus be negligible in LSC residing in the BM niche.

Of direct translational relevance, genetic inhibition of ADAM10 significantly reduced leukemic growth and LSC numbers in the BM rendering ADAM10 an interesting treatment target. Several inhibitors targeting ADAM10 are available, with GI254023X and Aderbasib (INCB7839) as the most advanced examples, the latter tested in a phase II study on breast cancer and currently studied in children with glioblastoma [27, 34, 36, 37, 58] (NCT01254136, NCT04295759). Our data support adding ADAM10-targeting drugs to conventional therapeutic regimens in AL patients, at best upon tumor specificity to avoid adverse effects [27, 33, 36]. Most recently, a therapeutic approach using ADAM10-targeting CAR-T cells has been reported to be effective against colon cancer in vivo [51].

The substrate(s) of ADAM10 regulating leukemic growth remain unclear. NOTCH1 represents an interesting candidate because it plays an essential role in mature B-cell malignancies such as chronic lymphoblastic leukemia or B-cell lymphoma and in T-ALL. However, NOTCH1 seems dispensable for BCP-ALL; none of the 31 leukemia cell lines studied previously expressed the NOTCH1 protein, and we were unable to detect NOTCH1 in any leukemia cell line supernatant (Figs. 5D, E and S14A, B) [33, 50, 59–61]. Together, targets other than NOTCH1 appear more likely to mediate the ADAM10 sheddase function in BCP-ALL.

Instead, we identified five proteins commonly affected by ADAM10 across the SEM and Nalm-6 secretomes, including the chaperones HSP90α and APP. Both proteins have been implicated in cancer progression in their dependence on ADAM10, making it tempting to speculate that these proteins may contribute to mediating the ADAM10 phenotype in BCP-ALL [62–64]. Further studies are required to validate ADAM10 substrates, which represent powerful therapeutic targets.

Taken together, in-depth proteomic profiling together with CRISPR‒Cas9 screens in PDX identified ADAM10 as an in vivo vulnerability in leukemia cells and LSC and indicate that ADAM10 may represent a therapeutic target to treat acute leukemias.

## Conclusion

In conclusion, ADAM10 might represent a novel therapeutic target to treat acute leukemias, both ALL and AML. Ultra-sensitive proteomics allowed addressing the minute population of in vivo dormant PDX leukemia stem cells, while in vivo CRISPR/Cas9 dropout screens revealed leukemia dependence on ADAM10. Inhibiting ADAM10 affected the leukemia-niche interaction, eliminated leukemia stem cells and fostered the anti-leukemia effect of conventional chemotherapy. It will be attractive to use our advanced technologies to investigate additional therapeutic targets in leukemia and to further explore ADAM10 inhibitors as components of chemotherapy regimens for acute leukemias.

## Supplementary Information

**Additional file 1: Supplemental Table 1.** PDX Patient data. **Supplemental Table 2.** LRC vs. Non-LRC proteome. **Supplemental Table 3.** Surface molecules library genes. **Supplemental Table 4.** sgRNA oligo sequences. **Supplemental Table 5.** NGS PCR primers. **Supplemental Table 6.** Gini indices – ALL-199 BM & SPL samples. **Supplemental Table 7.** Gini indices – Sublibrary plasmid & ALL-265 BM & SPL samples. **Supplemental Table 8.** SEM ADAM10 KO proteome. **Supplemental Table 9.** NALM-6 & SEM secretome. **Supplemental Table 10.** PDX transcriptome. **Supplemental Table 11.** PDX proteome - significant genes. **Supplemental Table 12.** Competitive LDTA. **Supplemental Figure S1.** Ultra-sensitive diaPASEF proteome workflow and proteomic characterization of slow-cycling PDX ALL cells. **Supplemental Figure 2.** Quality controls for generating Split-Cas9-transgenic PDX models. **Supplemental Figure S3.** Quality controls for generating CRISPR-Cas9 library-transgenic PDX models. **Supplemental Figure S4.** Nested PCR. **Supplemental Figure S5.** Quality controls for the in vivo CRISPR dropout screens. **Supplemental Figure S6.** Dropouts of the in vivo CRISPR screens in PDX ALL samples. **Supplemental Figure S7.** In vivo competitive validation assay. **Supplemental Figure S8.** Quality controls for in vivo validation assays for CXCR4 and ITGB1. **Supplemental Figure S9.** ADAM10 expression in tumor cells and their impact on patient survival. **Supplemental Figure S10.** ADAM10 in vivo validation assay. **Supplemental Figure S11.** ADAM10 reconstitution in HEK293T cells. **Supplemental Figure S12.** Pathway enrichment results of ADAM10 KO proteome analyses in SEM cells. **Supplemental Figure S13.** Secretome analysis of ADAM10 KO cells. **Supplemental Figure S14.** ADAM10 KO transcriptome and proteome analyses in ALL PDX cells. **Supplemental Figure S15.** Quality controls for experiments on cell cycle and apoptosis. **Supplemental Figure S16.** Quality controls and raw data for Figure 6BC. **Supplemental Figure S17.** ADAM10 inhibits colony formation in PDX AML cells in vitro. **Supplemental Figure S18.** ADAM10 KO increases the anti-leukemia efficacy of AraC and cyclo in vivo. **Supplemental Figure S19.** ADAM10 KO increases the anti-leukemia efficacy of AraC, Daunorubicin and Doxorubicin in vitro.

**Additional file 2.** Materials and Methods.

## Data Availability

The datasets analyzed during the current study are available through the referenced publications. The gene expression data were made publicly available through the Gene Expression Omnibus Website (GSE139553). All mass spectrometry raw data and output data of this study have been deposited to the ProteomeXchange Consortium via the PRIDE partner repository. Project accession: PXD036223

## Abbreviations

∆MP: Metalloproteinase lacking ADAM10 variant
ACT: Catalytically active ADAM10 variant
ADAM10: A disintegrin and metalloproteinase domain-containing protein 10
AL: Acute leukemias
ALL: Acute lymphoblastic leukemia
AML: Acute myeloid leukemia
APP: Amyloid-beta precursor protein
AraC: Cytarabine
B-ALL: B-cell Acute lymphoblastic leukemia
BCP: B-cell precursor
BM: Bone marrow
CAR-T: Chimeric antigen receptor T cells
Cas9: CRISPR associated protein 9
CD: Cluster of Differentiation
CD19: B-lymphocyte antigen CD19
CD79A: B-cell antigen receptor complex-associated protein alpha chain
CD81: Tetraspanin-28
CI: Confidence interval
CN: Cytogenetically normal
CP: Cytoplasmic domain
CR: Cysteine-rich domain
CRISPR: Clustered Regularly Interspaced Short Palindromic Repeats
CTRL: Control
CTSB: Cathepsin B
CXCR4: C-X-C chemokine receptor type 4
DI: Disintegrin domain
diaPASEF: Data-independent acquisition method using a parallel accumulation–serial fragmentation
DMSO: Dimethyl sulfoxide
ELDA: Extreme Limiting Dilution Analysis
F11R: Junctional adhesion molecule A
FACS: Fluorescence Activated Cell Sorting
FDR: False discovery rate
FLOT1: Flotillin 1
FLT3: Fms-like tyrosine kinase 3
G1: Gap phase 1
G2/M: Gap phase 2 / mitosis
GAPDH: Glyceraldehyde 3-phosphate dehydrogenase
GEPDX: Genetically engineered patient-derived xenograft
GFP: Green fluorescent protein
GO: Gene ontology
GOI: Gene of interest
GSEA: Gene set enrichment analysis
H-2Kk: H-2Kk antigen is expressed on mouse MHC class I–positive cells
HSP90α: Heat shock protein 90 α (family class A member 1)
IDE: Insulin degrading enzyme
IQR: Inter-quartile distance
iRFP: Near-infrared fluorescent protein
ITGA4: Integrin alpha 4
ITGA7: Integrin alpha 7
ITGB1: Integrin beta 1
ITGB2: Integrin beta 2
JUP1: Junction plakoglobin 1
KEGG: Kyoto Encyclopedia of Genes and Genomes
KO: Knockout
LDTA: Limiting dilution transplantation assay
LIC: Leukemia-initiating cells
LRC: Label-retaining cells
LSC: Leukemia stem cells
MACS: Magnetic-activated cell sorting
MAGeCK: Model-based Analysis of Genome-wide CRISPR/Cas9 Knockout
membr.: Membrane
mRNA: Messenger ribonucleic acid
MS: Mass spectrometry
mTagBFP: Monomeric blue fluorescent protein
NGS: Next generation sequencing
NOTCH1: Neurogenic locus notch homolog protein 1
ns: Not significant
OXPHOS: Oxidative phosphorylation
PBS: Phosphate Buffered Saline
PDX: Patient-derived xenograft
PRO: Prodomain
qRT-PCR: Quantitative real-time polymerase chain reaction
RNA: Ribonucleic acid
RNP: Ribonucleoprotein
RRA: Robust ranking algorithm
S: Synthesis phase
sgRNA: Short guide RNA
SLC19A1: Folate transporter 1
SLC3A2: 4F2 cell-surface antigen heavy chain
SOT: Start of therapy
SP: Signal peptide
SPG7: Paraplegin
Syntaxin 4: Membrane integrated Q-SNARE proteins participating in exocytosis family member 4
T-ALL: T-lymphoblastic leukemia
TCGA: The Cancer Genome Atlas
TFRC: Transferrin receptor protein 1
TIMS: Thermal ionization mass spectrometry
TM: Transmembrane domain
VDAC2: Voltage dependent anion channel 2
β -Actin: Beta-actin
