# Supplementary material for: Combined proteomics and CRISPR‒Cas9 screens in PDX identify ADAM10 as essential for leukemia in vivo
Source: Mol Cancer. 2023 Jul 8;22:107. doi: 10.1186/s12943-023-01803-0 (PMC10329331; doi:10.1186/s12943-023-01803-0)
Supplement: Supplementary file 1 — Additional file 1: Supplemental Table 1. PDX Patient data. Supplemental Table 2. LRC vs. Non-LRC proteome. Supplemental Table 3. Surface molecules library genes. Supplemental Table 4. sgRNA oligo sequences. Supplemental Table 5. NGS PCR primers. Supplemental Table 6. Gini indices – ALL-199 BM & SPL samples. Supplemental Table 7. Gini indices – Sublibrary plasmid & ALL-265 BM & SPL samples. Supplemental Table 8. SEM ADAM10 KO proteome. Supplemental Table 9. NALM-6 & SEM secretome. Supplemental Table 10. PDX transcriptome. Supplemental Table 11. PDX proteome - significant genes. Supplemental Table 12. Competitive LDTA. Supplemental Figure S1. Ultra-sensitive diaPASEF proteome workflow and proteomic characterization of slow-cycling PDX ALL cells. Supplemental Figure 2. Quality controls for generating Split-Cas9-transgenic PDX models. Supplemental Figure S3. Quality controls for generating CRISPR-Cas9 library-transgenic PDX models. Supplemental Figure S4. Nested PCR. Supplemental Figure S5. Quality controls for the in vivo CRISPR dropout screens. Supplemental Figure S6. Dropouts of the in vivo CRISPR screens in PDX ALL samples. Supplemental Figure S7. In vivo competitive validation assay. Supplemental Figure S8. Quality controls for in vivo validation assays for CXCR4 and ITGB1. Supplemental Figure S9. ADAM10 expression in tumor cells and their impact on patient survival. Supplemental Figure S10. ADAM10 in vivo validation assay. Supplemental Figure S11. ADAM10 reconstitution in HEK293T cells. Supplemental Figure S12. Pathway enrichment results of ADAM10 KO proteome analyses in SEM cells. Supplemental Figure S13. Secretome analysis of ADAM10 KO cells. Supplemental Figure S14. ADAM10 KO transcriptome and proteome analyses in ALL PDX cells. Supplemental Figure S15. Quality controls for experiments on cell cycle and apoptosis. Supplemental Figure S16. Quality controls and raw data for Figure 6BC. Supplemental Figure S17. ADAM10 inhibits colony formation in PDX AML cells in [file 12943_2023_1803_MOESM1_ESM.pdf]

Supplemental Data

for

Combined proteomics and CRISPR–Cas9 screens in PDX identify ADAM10 as essential for leukemia *in vivo*

Ehsan Bahrami[1*], Jan Philipp Schmid[1,2*], Vindi Jurinovic[1,3], Martin Becker[1,] Anna-Katharina Wirth[1], Romina Ludwig[1,2], Sophie Kreissig[4], Tania Vanessa Duque Angel[1], Diana Amend[1], Katharina Hunt[1], Rupert Öllinger[5,6], Roland Rad[2,5,6], Joris Maximilian Frenz[7,8], Maria Solovey[9,10], Frank Ziemann[3], Matthias Mann[11], Binje Vick[1,2], Christian Wichmann[4], Tobias Herold[1,2,3], Ashok Kumar Jayavelu[7,8,11#], Irmela Jeremias[1,2,12#^]

This PDF file contains:

Supplemental Tables

Supplemental Figures and Legends

Supplemental Table 1 – PDX Patient data

UPN	leading genetic abnormality	Disease	subtype	mutations (panel)[1,2]	ID/R	m/f	age [y]	Passaging time in NSG mice [d]
**ALL-50**	t(1;19) TCF3::PBX1	ALL	BCP	*NA*	ID	f	7	<50
**ALL-706**	t(4;11) KMT2A::AFF1	ALL	BCP	*NA*	ID	f	5	<50
**ALL-763**	t(4;11) KMT2A::AFF1	ALL	BCP	*NA*	ID	f	17	<50
**ALL-817**	t(4;11) KMT2A::AFF1	ALL	BCP	*NA*	ID	f	74	<50
**ALL-435**	t(11;19) KMT2A::MLLT1	ALL	BCP	*NA*	ID	m	0.5	<50
**ALL-502**	IGH::DUX4	ALL	BCP	*NA*	R1	f	9	<70
**ALL-697**	IGH::BCL2	ALL	BCP	*NA*	ID	unknown	<18	<50
**ALL-199**	germline +21; somatic homozygous 9p deletion (CDKN2A); P2RY8::CRLF2	ALL	BCP	KRAS	R2	f	7	<50
**ALL-265**	High Hyperdiploidy	ALL	BCP	CSMD1, HERC1, KMT2D, PRRT2	R1	f	5	<50
**ALL-787(p)**	low hypodiploidy/near triploidy (LH-NT)	ALL	BCP	*NA*	R3	f	81	<90
**ALL-827(p)**					R4		82	<70
**ALL-1124**	complex karyotype	ALL	BCP	*NA*	ID	m	12	<50
**ALL-230**	t(1;14)(p32;q11) T-ALL	ALL	T-ALL	NOTCH1	ID	m	4	<50
**ALL-843**	T-ALL	ALL	T-ALL	*NA*	R1	m	66	<70
**AML-415**	CN with NPM1 and FLT3-ITD	AML	NA	DNMT3A, IDH1, NPM1, FLT3-ITD	R2	f	68	>90
**AML-579**	CN with NPM1 and FLT3-ITD	AML	FAB M5	DNMT3A, IDH1, NPM1, FLT3-ITD	R1	m	50	<90
**AML-573**	t(5;11)(p1?;q?13)	AML	FAB M1	DNMT3A, IDH2, FLT3-ITD, WT1	R1	f	64	>90
**AML-491(p)**	del7q (7q21.13 q36.3)	AML	FAB M2	DNMT3A, RUNX1, BCOR, NRAS, KRAS, ETV6, PTPN11	R1	f	53	<90
**AML-661(p)**	del7q (7q21.13 q36.3); del6p (6p25.3 p21.1)			DNMT3A, RUNX1, BCOR, NRAS, ETV6, PTPN11, JAK1, EZH2	R2		54	<70
**AML-388**	t(6;11)(q27;q23) KMT2A::AFDN (MLLT4)	AML	FAB M4	KRAS	ID	m	57	<50
**AML-393**	ins(10;11)(p12;q23q23) KMT2A::MLLT10	AML	FAB M4	BCOR, KRAS	R1	f	47	<50
**AML-372**	complex karyotype, including -17, -7, ETV-deletion, ATM-deletion	AML	FAB M1	TP53, KRAS	R2	m	41	<70
**AML-602**	complex karyotype	AML	NA	DNMT3A, TET2, NPM1, FLT3-ITD, CEBPA, JAK3	R1	f	40	<70
**AML-356**	*NA*	AML	FAB M5	KRAS, U2AF1	R	m	5	<50
**AML-663**	*NA*	AML	FAB M2	FLT3-ITD, WT1	R	f	13	<50

UPN: unique patient number; ALL: acute lymphoblastic leukemia; AML: acute myeloid leukemia; (p): sample pair; BCP: B cell precursor; ph like: Philadelphia-like; CN: cytogenetically normal; NA: not analyzed; ID: initial diagnosis; R: relapse; m: male; f: female; y: years; d: days
1 Lorenz et al., Leukemia 2019, PMID: 30842609
2 Metzeler KH et al., Blood 2016, PMID: 27288520

Supplemental Table 2 – LRC vs. Non-LRC proteome – Upper part, see Suppl. Table 2

Column1<br>nLRC_1	Column2<br>nLRC_2	Column3<br>nLRC_3		Column6<br>LRC_1	Column7<br>LRC_2	Column8<br>LRC_3		Column23<br>Genes
#!{Type}E	E	E		E	E	E		T
#!{C:Group1}nLRC	nLRC	nLRC		LRC	LRC	LRC		
11.61878	12.99376	11.22788		12.02746	12.19467	12.38708		HDLBP
12.11858	12.4441	12.73828		12.22397	12.07813	11.87171		NUDT4B
12.29316	12.22589	12.06994		13.65584	11.00215	13.83095		NUDT3;NUDT4;NUDT4B
12.39949	13.33321	13.3471		12.78133	13.29705	11.0231		NUDT4;NUDT4B
10.61007	10.52306	10.33142		10.93882	10.99496	12.95221		IGLV3-12;IGLV3-21;IGLV3-9
12.45259	12.38719	11.06152		11.74641	11.84156	12.83838		IGKV2-28;IGKV2-29;IGKV2-30;…
11.72513	12.63674	12.12534		12.19487	12.38246	12.36279		EIF2B4
10.91124	11.17474	12.23462		12.23649	12.45222	11.01595		ZMYND8
12.56838	12.18999	11.48319		10.93162	10.27974	10.30491		PSMD1
10.79011	12.6468	11.91793		10.21494	11.67428	11.21081		LRMDA
18.19202	18.46446	18.9828		19.26372	19.22848	18.96522		TPM3
11.66088	11.10588	12.35421		11.77921	12.04902	12.26498		VPS11
13.32985	12.0746	11.48956		11.50817	11.56434	12.16545		SREK1
12.91888	12.83554	12.36136		12.93777	12.66477	12.37868		NUMA1
14.50018	14.60738	14.49522		14.43997	14.16576	14.06056		RAVER1
12.01031	12.28892	12.21678		12.47489	12.35639	12.49381		PLEKHA2
12.8688	13.31327	13.91608		12.73116	13.33807	10.96839		CYP2D6;CYP2D7
12.93238	14.31933	13.037		13.1346	13.05554	13.02413		HUWE1
11.01205	11.65119	12.58829		13.25149	12.88106	13.85731		SPAG9
11.21036	11.11242	11.49361		10.71762	10.99949	10.71142		ERG
14.89717	14.35882	13.67368		13.32839	14.04088	13.1052		LOC102724023
16.23399	16.54399	16.51826		16.42968	16.39089	16.30101		LOC102724023
11.32649	12.86638	12.7996	…	12.04358	12.25133	12.70539	…	ATRX
10.9043	12.50433	11.63305		11.52347	11.97768	12.40394		SMIM26
10.3462	10.70521	13.11456		12.5587	12.48027	10.70246		GPS1
11.85931	12.2248	12.43945		11.83891	12.04277	12.12497		AAK1
9.361565	10.84801	10.51739		12.19055	10.94712	10.0709		CLASRP
12.27857	12.47942	12.32376		11.90798	12.25883	11.84106		USP19
11.66259	11.03918	11.91998		10.88369	10.67962	10.75922		GSTZ1
9.535015	10.07828	9.749427		10.61564	10.04436	10.6433		PUM2
12.92984	13.10705	11.29519		10.27168	11.40984	11.33564		TCF3
11.44952	10.99507	11.95019		11.92931	12.11206	12.23007		MFF
11.39144	11.62494	12.95584		12.51208	12.56499	12.61736		UTRN
10.43103	10.92976	11.89403		12.44802	12.47265	12.66371		PRR4
11.86447	11.39359	11.96182		12.02101	12.12358	11.77936		RNF213
12.23479	13.39636	16.0919		14.40032	14.27777	15.4109		IGHV6-1
13.87086	13.45873	12.66465		12.78386	12.66687	13.09481		C11orf98
10.59256	10.25609	13.08425		13.88984	14.22171	14.48298		IGHV3OR16-9
10.77967	11.58792	12.34791		9.979081	10.62012	13.8402		GATD3A;GATD3B
16.09556	15.22921	15.20913		14.44556	14.64085	15.10909		LOC102724023
14.6405	13.21682	13.32397		13.67025	13.83477	13.67135		CAST
13.80341	14.53597	14.0948		13.73352	13.66401	13.70205		PPA2
12.53143	11.61524	11.84567		10.46991	11.1209	11.52418		DGLUCY
14.8909	14.50199	14.64153		13.99139	13.82567	14.22683		PRKCSH
13.01496	12.95477	13.43952		10.55601	11.76286	9.868825		IQSEC1
11.90406	9.435396	9.849294		11.19338	11.55515	11.28074		IGKV3-20;IGKV3D-20

Supplemental Table 3 - Surface molecules library genes – Upper part, see Suppl. Table 3

Gene name	Gene ID	Chromosomal Location	Location	Assembly
ABCA1	19	9q31.1	NC_000009.12 (104781006..104928155, complement)	GRCh38.p14
ADAM10	102	15q21.3	NC_000015.10 (58588809..58749707, complement)	GRCh38.p14
ADAM19	8728	5q33.3	NC_000005.10 (157477304..157575775, complement)	GRCh38.p14
ADAM8	101	10q26.3	NC_000010.11 (133262423..133276868, complement)	GRCh38.p14
ADGRE5	976	19p13.12	NC_000019.10 (14381144..14408725)	GRCh38.p14
ALCAM	214	3q13.11	NC_000003.12 (105366909..105576900)	GRCh38.p14
ANTXR1	84168	2p13.3	NC_000002.12 (69013144..69249327)	GRCh38.p14
BSG	682	19p13.3	NC_000019.10 (571283..583493)	GRCh38.p14
CADM4	199731	19q13.31	NC_000019.10 (43622368..43641984, complement)	GRCh38.p14
CD163	9332	12p13.31	NC_000012.12 (7470811..7503777, complement)	GRCh38.p14
CD37	951	19q13.33	NC_000019.10 (49335406..49340606)	GRCh38.p14
CD44	960	11p13	NC_000011.10 (35139171..35232402)	GRCh38.p14
CD53	963	1p13.3	NC_000001.11 (110871210..110899922)	GRCh38.p14
CD68	968	17p13.1	NC_000017.11 (7579638..7582111)	GRCh38.p14
CD69	969	12p13.31	NC_000012.12 (9752486..9760901, complement)	GRCh38.p14
CD70	970	19p13.3	NC_000019.10 (6581648..6591150, complement)	GRCh38.p14
CD79A	973	19q13.2	NC_000019.10 (41877279..41881372)	GRCh38.p14
CD81	975	11p15.5	NC_000011.10 (2376180..2397397)	GRCh38.p14
CDH13	1012	16q23.3	NC_000016.10 (82626969..83800640)	GRCh38.p14
CDH5	1003	16q21	NC_000016.10 (66366691..66404784)	GRCh38.p14
CDH6	1004	5p13.3	NC_000005.10 (31193686..31329146)	GRCh38.p14
CEACAM6	4680	19q13.2	NC_000019.10 (41755530..41772211)	GRCh38.p14
CNNM4	26504	2q11.2	NC_000002.12 (96760902..96811874)	GRCh38.p14
CSF1R	1436	5q32	NC_000005.10 (150053295..150113365, complement)	GRCh38.p14
CXCR4	7852	2q22.1	NC_000002.12 (136114349..136118149, complement)	GRCh38.p14
EMP1	2012	12p13.1	NC_000012.12 (13196726..13219941)	GRCh38.p14
F11R	50848	1q23.3	NC_000001.11 (160995211..161021152, complement)	GRCh38.p14
F2RL3	9002	19p13.11	NC_000019.10 (16888999..16892606)	GRCh38.p14
FMOD	2331	1q32.1	NC_000001.11 (203340628..203351122, complement)	GRCh38.p14
FN1	2335	2q35	NC_000002.12 (215360865..215436068, complement)	GRCh38.p14
GPR183	1880	13q32.3	NC_000013.11 (99294539..99307399, complement)	GRCh38.p14
HCST	10870	19q13.12	NC_000019.10 (35902529..35904377)	GRCh38.p14
HLA-DQB1	3119	6p21.32	NC_000006.12 (32659467..32666657, complement)	GRCh38.p14
HLA-E	3133	6p22.1	NC_000006.12 (30489509..30494194)	GRCh38.p14
HSPG2	3339	1p36.12	NC_000001.11 (21822244..21937310, complement)	GRCh38.p14
IGF2	3481	11p15.5	NC_000011.10 (2129117..2149566, complement)	GRCh38.p14
IGFALS	3483	16p13.3	NC_000016.10 (1790413..1794908, complement)	GRCh38.p14
IGFBP2	3485	2q35	NC_000002.12 (216632828..216664436)	GRCh38.p14
IGFBP3	3486	7p12.3	NC_000007.14 (45912245..45921272, complement)	GRCh38.p14
IGFBP4	3487	17q21.2	NC_000017.11 (40443450..40457725)	GRCh38.p14
IGSF3	3321	1p13.1	NC_000001.11 (116574398..116667755, complement)	GRCh38.p14
IL10	3586	1q32.1	NC_000001.11 (206767602..206772494, complement)	GRCh38.p14
IL1R2	7850	2q11.2	NC_000002.12 (101991960..102028544)	GRCh38.p14
ITGA5	3678	12q13.13	NC_000012.12 (54395261..54419266, complement)	GRCh38.p14
ITGB1	3688	10p11.22	NC_000010.11 (32900318..32958230, complement)	GRCh38.p14
ITGB2	3689	21q22.3	NC_000021.9 (44885953..44928815, complement)	GRCh38.p14
ITM2C	81618	2q37.1	NC_000002.12 (230864185..230879254)	GRCh38.p14
KCNN4	3783	19q13.31	NC_000019.10 (43766533..43780973, complement)	GRCh38.p14
KIT	3815	4q12	NC_000004.12 (54657957..54740715)	GRCh38.p14
KRT1	3848	12q13.13	NC_000012.12 (52674736..52680407, complement)	GRCh38.p14

Supplemental Table 4 - Upper part of sgRNA oligo sequences see Suppl. Table 4

sgRNA (fwd)	Sequence (5' - 3')	sgRNA (rev)	Sequence (5' - 3')
ABCA1-1F	tcccGGTGGCATGGCAGGACTACGT	ABCA1-1R	aaacACGTAGTCCTGCCATGCCACC
ABCA1-2F	tcccGCGTACCGCATGTCCTCAAAG	ABCA1-2R	aaacCTTTGAGGACATGCGGTACGC
ABCA1-3F	tcccGGGACACGCCCAGCTTCAAGT	ABCA1-3R	aaacACTTGAAGCTGGGCGTGTCCC
ABCA1-4F	tcccGGCGAGTACTTCGTTCCAACA	ABCA1-4R	aaacTGTTGGAACGAAGTACTCGCC
ABCA1-5F	tcccGGCAGCTCCCATATTCCCCTG	ABCA1-5R	aaacCAGGGGAATATGGGAGCTGCC
ADAM10-1F	tcccGCCCATAAATACGGTCCTCAG	ADAM10-1R	aaacCTGAGGACCGTATTTATGGGC
ADAM10-2F	tcccGTTTCAACCTACGAATGAAGA	ADAM10-2R	aaacTCTTCATTCGTAGGTTGAAAC
ADAM10-3F	tcccGTTCCATCAATAACAGACCCA	ADAM10-3R	aaacTGGGTCTGTTATTGATGGAAC
ADAM10-4F	tcccGCCGTTTCCCAAATATTGGTG	ADAM10-4R	aaacCACCAATATTTGGGAAACGGC
ADAM10-5F	tcccGGTAATGTGAGAGACTTTGGG	ADAM10-5R	aaacCCCAAAGTCTCTCACATTACC
ADAM19-1F	tcccGATGCCTCTAATTGTACCCTG	ADAM19-1R	aaacCAGGGTACAATTAGAGGCATC
ADAM19-2F	tcccGACTCACCTCCAATTTCCGTG	ADAM19-2R	aaacCACGGAAATTGGAGGTGAGTC
ADAM19-3F	tcccGTGCCCAGAGATGCGAAGTGT	ADAM19-3R	aaacACACTTCGCATCTCTGGGCAC
ADAM19-4F	tcccGCCTCTAAATAATCAGCCACG	ADAM19-4R	aaacCGTGGCTGATTATTTAGAGGC
ADAM19-5F	tcccGTGTTCAATGGATGCAACAGG	ADAM19-5R	aaacCCTGTTGCATCCATTGAACAC
ADAM8-1F	tcccGAGTCCGGGTACCCCTCTACG	ADAM8-1R	aaacCGTAGAGGGGTACCCGGACTC
ADAM8-2F	tcccGCAAACAGGTTCCCACACACG	ADAM8-2R	aaacCGTGTGTGGGAACCTGTTTGC
ADAM8-3F	tcccGCCACATACAGCTCCACGTAG	ADAM8-3R	aaacCTACGTGGAGCTGTATGTGGC
ADAM8-4F	tcccGTCAGAAACTCAACTTCCGTG	ADAM8-4R	aaacCACGGAAGTTGAGTTTCTGAC
ADAM8-5F	tcccGCCAGCTTAGACTCACCAAGT	ADAM8-5R	aaacACTTGGTGAGTCTAAGCTGGC
ADGRE5-1F	tcccGACCGTCACAAGTCTCCGTCG	ADGRE5-1R	aaacCGACGGAGACTTGTGACGGTC
ADGRE5-2F	tcccGTATGGCTCATTATGACGTGG	ADGRE5-2R	aaacCCACGTCATAATGAGCCATAC
ADGRE5-3F	tcccGGGCCTCACCTGTGTTCGAAG	ADGRE5-3R	aaacCTTCGAACACAGGTGAGGCCC
ADGRE5-4F	tcccGCCACCTTGAGTCCTCCGATG	ADGRE5-4R	aaacCATCGGAGGACTCAAGGTGGC
ADGRE5-5F	tcccGCTTGGAATGCAGGTTCAAGG	ADGRE5-5R	aaacCCTTGAACCTGCATTCCAAGC
ALCAM-1F	tcccGTGTGTGCATGCTAGTAACTG	ALCAM-1R	aaacCAGTTACTAGCATGCACACAC
ALCAM-2F	tcccGCTTACACACTGACGGATGTG	ALCAM-2R	aaacCACATCCGTCAGTGTGTAAGC
ALCAM-3F	tcccGTGAGGTACGTCAAGTCGGCA	ALCAM-3R	aaacTGCCGACTTGACGTACCTCAC
ALCAM-4F	tcccGATCCAGATGGCAATATCACA	ALCAM-4R	aaacTGTGATATTGCCATCTGGATC
ALCAM-5F	tcccGGCAGATATTGTGCATGACAC	ALCAM-5R	aaacGTGTCATGCACAATATCTGCC
ANTXR1-1F	tcccGCTTCCGACATGCCCGCAACG	ANTXR1-1R	aaacCGTTGCGGGCATGTCGGAAGC
ANTXR1-2F	tcccGATAAAGGACATTCTCAACTG	ANTXR1-2R	aaacCAGTTGAGAATGTCCTTTATC
ANTXR1-3F	tcccGGTTATGACTTACACAGTGTG	ANTXR1-3R	aaacCACACTGTGTAAGTCATAACC
ANTXR1-4F	tcccGGGAGACACTTACATGCATGA	ANTXR1-4R	aaacTCATGCATGTAAGTGTCTCCC
ANTXR1-5F	tcccGCTGGCCCCTCTGCTGCACTG	ANTXR1-5R	aaacCAGTGCAGCAGAGGGGCCAGC
BSG-1F	tcccGGTCGTCAGAACACATCAACG	BSG-1R	aaacCGTTGATGTGTTCTGACGACC
BSG-2F	tcccGGGATCGCAACCACCTGACCC	BSG-2R	aaacGGGTCAGGTGGTTGCGATCCC
BSG-3F	tcccGCTTGAATGACAGCGCCACAG	BSG-3R	aaacCTGTGGCGCTGTCATTCAAGC
BSG-4F	tcccGGTGGACTCCGACGACCAGTG	BSG-4R	aaacCACTGGTCGTCGGAGTCCACC
BSG-5F	tcccGCATCTCCATCGACACGCTCG	BSG-5R	aaacCGAGCGTGTCGATGGAGATGC
CADM4-1F	tcccGGTGGACCGTAAGGACGACGG	CADM4-1R	aaacCCGTCGTCCTTACGGTCCACC
CADM4-2F	tcccGTGTTGACGTGTGCTGTCACG	CADM4-2R	aaacCGTGACAGCACACGTCAACAC
CADM4-3F	tcccGTTGCCTAACAGACTCCCCCA	CADM4-3R	aaacTGGGGGAGTCTGTTAGGCAAC
CADM4-4F	tcccGGTTACCTAGTACCGTGAGCG	CADM4-4R	aaacCGCTCACGGTACTAGGTAACC
CADM4-5F	tcccGACAGACAGAGAACGTGACAG	CADM4-5R	aaacCTGTCACGTTCTCTGTCTGTC
CD163-1F	tcccGCAAAGACGATGAATTGCACG	CD163-1R	aaacCGTGCAATTCATCGTCTTTGC
CD163-2F	tcccGGTGAAGCATGGTGACACGTG	CD163-2R	aaacCACGTGTCACCATGCTTCACC
CD163-3F	tcccGACTGGCGTTAACTCGACCAA	CD163-3R	aaacTTGGTCGAGTTAACGCCAGTC
CD163-4F	tcccGGATTCAGAGACTGTTAGGGA	CD163-4R	aaacTCCCTAACAGTCTCTGAATCC

Supplemental Table 5 – NGS PCR primers

	Name	Seq
1st PCR	cppt-NGS-F	GGGTACAGTGCAGGGGAAAGAATA
	EF1a-NGS-R	gagccagtacacgacatcactttcc
2nd PCR	P5-H1-S0	AATGATACGGCGACCACCGAGATCTACACTCTTTCCCTACACGACGCTCTTCCGATCTTGTATGAGACCACTCTTTCCCG
	P5-H1-S1	AATGATACGGCGACCACCGAGATCTACACTCTTTCCCTACACGACG CTCTTCCGATCTCTGTATGAGACCACTCTTTCCCG
	P5-H1-S2	AATGATACGGCGACCACCGAGATCTACACTCTTTCCCTACACGACG CTCTTCCGATCTGCTGTATGAGACCACTCTTTCCCG
	P5-H1-S3	AATGATACGGCGACCACCGAGATCTACACTCTTTCCCTACACGACG CTCTTCCGATCTAGCTGTATGAGACCACTCTTTCCCG
	P5-H1-S4	AATGATACGGCGACCACCGAGATCTACACTCTTTCCCTACACGACG CTCTTCCGATCTCAACTGTATGAGACCACTCTTTCCCG
	P5-H1-S5	AATGATACGGCGACCACCGAGATCTACACTCTTTCCCTACACGACG CTCTTCCGATCTTGCACCTGTATGAGACCACTCTTTCCCG
	P5-H1-S6	AATGATACGGCGACCACCGAGATCTACACTCTTTCCCTACACGACG CTCTTCCGATCTACGCAACTGTATGAGACCACTCTTTCCCG
	P5-H1-S7	AATGATACGGCGACCACCGAGATCTACACTCTTTCCCTACACGACG CTCTTCCGATCTGAAGACCCTGTATGAGACCACTCTTTCCCG
	P7-Ef1-A01	CAAGCAGAAGACGGCATACGAGATcggttcaaGTGACTGGAGTTCAGACGTGTGCTCTTCCGATCTgagccagtacacgacatcactttc
	P7-Ef1-A11	CAAGCAGAAGACGGCATACGAGATattgtcaaGTGACTGGAGTTCAGACGTGTGCTCTTCCGATCTgagccagtacacgacatcactttc
	P7-Ef1-A12	CAAGCAGAAGACGGCATACGAGATtatgtcttGTGACTGGAGTTCAGACGTGTGCTCTTCCGATCTgagccagtacacgacatcactttc
	P7-Ef1-B01	CAAGCAGAAGACGGCATACGAGATATTGGATTGTGACTGGAGTTCAGACGTGTGCTCTT CCGATCTgagccagtacacgacatcactttc
	P7-Ef1-B02	CAAGCAGAAGACGGCATACGAGATatactcggGTGACTGGAGTTCAGACGTGTGCTCTTCCGATCTgagccagtacacgacatcactttc
	P7-Ef1-B03	CAAGCAGAAGACGGCATACGAGATtatgagaaGTGACTGGAGTTCAGACGTGTGCTCTTCCGATCTgagccagtacacgacatcactttc
	P7-Ef1-B04	CAAGCAGAAGACGGCATACGAGATgcacagttGTGACTGGAGTTCAGACGTGTGCTCTTCCGATCTgagccagtacacgacatcactttc
	P7-Ef1-B05	CAAGCAGAAGACGGCATACGAGATcgtggattGTGACTGGAGTTCAGACGTGTGCTCTTCCGATCTgagccagtacacgacatcactttc
	P7-Ef1-B11	CAAGCAGAAGACGGCATACGAGATATCACTGGGTGACTGGAGTTCAGACGTGTGCTCTT CCGATCTgagccagtacacgacatcactttc
	P7-Ef1-B12	CAAGCAGAAGACGGCATACGAGATCGCATCAAGTGACTGGAGTTCAGACGTGTGCTCTT CCGATCTgagccagtacacgacatcactttc
	P7-Ef1-C01	CAAGCAGAAGACGGCATACGAGATGCACGACCGTGACTGGAGTTCAGACGTGTGCTCTTCCGATCTgagccagtacacgacatcactttc
	P7-Ef1-C02	CAAGCAGAAGACGGCATACGAGATTACACTCCGTGACTGGAGTTCAGACGTGTGCTCTTCCGATCTgagccagtacacgacatcactttc
	P7-Ef1-C03	CAAGCAGAAGACGGCATACGAGATCGGTCTAAGTGACTGGAGTTCAGACGTGTGCTCTTCCGATCTgagccagtacacgacatcactttc
	P7-Ef1-C04	CAAGCAGAAGACGGCATACGAGATATGTTCGGGTGACTGGAGTTCAGACGTGTGCTCTT CCGATCTgagccagtacacgacatcactttc
	P7-Ef1-C05	CAAGCAGAAGACGGCATACGAGATCGTGGACCGTGACTGGAGTTCAGACGTGTGCTCTTCCGATCTgagccagtacacgacatcactttc
	P7-Ef1-C06	CAAGCAGAAGACGGCATACGAGATATTGAGCCGTGACTGGAGTTCAGACGTGTGCTCTTCCGATCTgagccagtacacgacatcactttc
	P7-Ef1-C07	CAAGCAGAAGACGGCATACGAGATTAGTTCGGGTGACTGGAGTTCAGACGTGTGCTCTTCCGATCTgagccagtacacgacatcactttc
	P7-Ef1-C08	CAAGCAGAAGACGGCATACGAGATCGGTGAGGGTGACTGGAGTTCAGACGTGTGCTCTTCCGATCTgagccagtacacgacatcactttc
	P7-Ef1-C09	CAAGCAGAAGACGGCATACGAGATCGTGAGTTGTGACTGGAGTTCAGACGTGTGCTCTTCCGATCTgagccagtacacgacatcactttc
	P7-Ef1-C10	CAAGCAGAAGACGGCATACGAGATATCAGATTGTGACTGGAGTTCAGACGTGTGCTCTTCCGATCTgagccagtacacgacatcactttc
	P7-Ef1-C11	CAAGCAGAAGACGGCATACGAGATTAGTGATTGTGACTGGAGTTCAGACGTGTGCTCTTCCGATCTgagccagtacacgacatcactttc
	P7-Ef1-C12	CAAGCAGAAGACGGCATACGAGATCGGTTCGGGTGACTGGAGTTCAGACGTGTGCTCTTCCGATCTgagccagtacacgacatcactttc
	P7-Ef1-E05	CAAGCAGAAGACGGCATACGAGATgcgttcaaGTGACTGGAGTTCAGACGTGTGCTCTTCCGATCTgagccagtacacgacatcactttc
	P7-Ef1-E06	CAAGCAGAAGACGGCATACGAGATcgcaagaaGTGACTGGAGTTCAGACGTGTGCTCTTCCGATCTgagccagtacacgacatcactttc
	P7-Ef1-E07	CAAGCAGAAGACGGCATACGAGATcgacagccGTGACTGGAGTTCAGACGTGTGCTCTTCCGATCTgagccagtacacgacatcactttc
	P7-Ef1-E08	CAAGCAGAAGACGGCATACGAGATcgactcggGTGACTGGAGTTCAGACGTGTGCTCTTCCGATCTgagccagtacacgacatcactttc
	P7-Ef1-E09	CAAGCAGAAGACGGCATACGAGATtacaagaaGTGACTGGAGTTCAGACGTGTGCTCTTCCGATCTgagccagtacacgacatcactttc
	P7-Ef1-E10	CAAGCAGAAGACGGCATACGAGATCGCAGATTGTGACTGGAGTTCAGACGTGTGCTCTT CCGATCTgagccagtacacgacatcactttc
	P7-Ef1-E11	CAAGCAGAAGACGGCATACGAGATATTGCTCCGTGACTGGAGTTCAGACGTGTGCTCTT CCGATCTgagccagtacacgacatcactttc
	P7-Ef1-E12	CAAGCAGAAGACGGCATACGAGATGCACTCGGGTGACTGGAGTTCAGACGTGTGCTCTT CCGATCTgagccagtacacgacatcactttc
	P7-Ef1-F1	CAAGCAGAAGACGGCATACGAGATATGTTCTTGTGACTGGAGTTCAGACGTGTGCTCTT CCGATCTgagccagtacacgacatcactttc
	P7-Ef1-F2	CAAGCAGAAGACGGCATACGAGATATGTCTCCGTGACTGGAGTTCAGACGTGTGCTCTT CCGATCTgagccagtacacgacatcactttc
	P7-Ef1-F3	CAAGCAGAAGACGGCATACGAGATGCACTCAAGTGACTGGAGTTCAGACGTGTGCTCTT CCGATCTgagccagtacacgacatcactttc
	P7-Ef1-F4	CAAGCAGAAGACGGCATACGAGATTAGTAGCCGTGACTGGAGTTCAGACGTGTGCTCTT CCGATCTgagccagtacacgacatcactttc
	P7-Ef1-F5	CAAGCAGAAGACGGCATACGAGATTAGTAGCCGTGACTGGAGTTCAGACGTGTGCTCTT CCGATCTgagccagtacacgacatcactttc
	P7-Ef1-F6	CAAGCAGAAGACGGCATACGAGATGCGTTCTTGTGACTGGAGTTCAGACGTGTGCTCTT CCGATCTgagccagtacacgacatcactttc
	P7-Ef1-F7	CAAGCAGAAGACGGCATACGAGATGCCAAGCCGTGACTGGAGTTCAGACGTGTGCTCTT CCGATCTgagccagtacacgacatcactttc

Supplemental Table 6 - Gini indices – ALL-199 BM & SPL samples

Label	Total sgRNA	Zero Counts	Gini Index
48179BM_S22_L001_R1_001	505	15	0.12
48180SPL_S17_L002_R1_001	505	14	0.09
48179BM_S22_L004_R1_001	505	15	0.11
48180BM_S23_L001_R1_001	505	13	0.10
48180BM_S23_L002_R1_001	505	13	0.10
48180BM_S23_L003_R1_001	505	13	0.10
48179SPL_S16_L001_R1_001	505	15	0.10
48179BM_S22_L003_R1_001	505	15	0.12
48179SPL_S16_L002_R1_001	505	14	0.09
48180SPL_S17_L001_R1_001	505	14	0.09
48180SPL_S17_L003_R1_001	505	14	0.09
48179SPL_S16_L003_R1_001	505	15	0.10
48179BM_S22_L002_R1_001	505	15	0.11
48180BM_S23_L004_R1_001	505	13	0.10
48180SPL_S17_L004_R1_001	505	14	0.09
48179SPL_S16_L004_R1_001	505	15	0.10

Supplemental Table 7 - Gini indices – Sublibrary plasmid & ALL-265 BM & SPL samples

Label	Total sgRNA	Zero Counts	Gini Index
Demultiplex_Fastq1_AACTGTGC_on_data_41_and_data_40	490	6	0.05
Demultiplex_Fastq1_AACTGTGC_on_data_43_and_data_42	490	63	0.30
104_S28_L001_R1_001	490	5	0.06
104_S28_L002_R1_001	490	7	0.06
104_S28_L003_R1_001	490	6	0.06
104_S28_L004_R1_001	490	7	0.06
48686BM_S26_L003_R1_001	490	6	0.07
48652BM_S24_L004_R1_001	490	6	0.07
48687SPL_S21_L002_R1_001	490	7	0.07
48686BM_S26_L001_R1_001	490	6	0.07
48686BM_S26_L002_R1_001	490	5	0.07
48652SPL_S18_L003_R1_001	490	6	0.07
48687SPL_S21_L004_R1_001	490	7	0.07
48652BM_S24_L002_R1_001	490	6	0.07
48653SPL_S19_L001_R1_001	490	4	0.07
48653BM_S25_L002_R1_001	490	5	0.07
48653BM_S25_L001_R1_001	490	5	0.07
48686SPL_S20_L001_R1_001	490	7	0.07
48687BM_S27_L003_R1_001	490	7	0.07
48653BM_S25_L003_R1_001	490	6	0.07
48652SPL_S18_L001_R1_001	490	5	0.07
48653BM_S25_L004_R1_001	490	5	0.07
48652SPL_S18_L002_R1_001	490	6	0.07
48653SPL_S19_L004_R1_001	490	4	0.06
48652SPL_S18_L004_R1_001	490	6	0.07
48687SPL_S21_L001_R1_001	490	7	0.07
48687BM_S27_L004_R1_001	490	6	0.07
48686SPL_S20_L004_R1_001	490	7	0.07
48652BM_S24_L001_R1_001	490	6	0.07
48687BM_S27_L001_R1_001	490	6	0.07
48653SPL_S19_L003_R1_001	490	4	0.07
48653SPL_S19_L002_R1_001	490	4	0.07
48686BM_S26_L004_R1_001	490	5	0.07
48687BM_S27_L002_R1_001	490	6	0.07
48687SPL_S21_L003_R1_001	490	7	0.07
48652BM_S24_L003_R1_001	490	6	0.07
48686SPL_S20_L002_R1_001	490	7	0.07
48686SPL_S20_L003_R1_001	490	7	0.07

Supplemental Table 8 – Upper part of SEM ADAM10 KO proteome, see Suppl. Table 8

SEM_sgNTcontrol_4	SEM_sgNTcontrol_3		SEM_Adam10sg4_2	SEM_Adam10sg5_1		Gene names
E	E		E	E		T
SEM_sgNTcontrol	SEM_sgNTcontrol		SEM_Adam10sg	SEM_Adam10sg		
NaN	26,20587		NaN	NaN		ELOF1
NaN	NaN		NaN	NaN		E2F8
31,722	31,68366		31,48106	31,56434		UBA6
31,5775	31,59054		31,51107	31,41084		ESYT2
26,92751	26,94514		27,02126	26,91376		MED19
28,34735	28,27932		28,12065	28,36394		KIAA1598
26,37048	26,26232		NaN	NaN		TMEM223
28,76809	28,54874		28,72423	28,77866		ILVBL
NaN	NaN		NaN	25,65401		PLEKHG3
26,37006	26,59007		NaN	26,52347		SH3PXD2B
NaN	NaN		24,68239	NaN		ZC3H12D
NaN	NaN		NaN	NaN		RALGAPB
NaN	NaN		26,69697	26,7124		ALG6
NaN	26,8992		26,84876	27,63256		AAR2
30,91413	30,9243		30,86172	30,75414		ADD1
NaN	NaN		NaN	27,66207		SLC39A7
26,94503	27,21002		26,78644	26,7748		HLA-DQB1
NaN	NaN		NaN	NaN		PHF1
28,74948	28,69826		29,64791	29,50949		TAPBP
28,83575	29,00788		28,80261	28,94218		EHMT2
NaN	24,25695		NaN	NaN		PSMB9
28,07398	28,69049	…	30,28736	29,68268	…	HLA-C
27,76192	27,67142		27,93629	27,84308		NBAS
23,738	NaN		NaN	NaN		TARSL2
NaN	NaN		NaN	26,16423		KIAA1467
29,13526	28,8573		28,61635	28,66363		VWA8
29,04698	29,01962		29,29266	29,24742		SBNO1
NaN	NaN		NaN	NaN		FAM221A
28,98025	28,77903		29,42168	29,58654		GTPBP10
28,74512	28,53025		28,48155	28,47402		WDR91
26,38571	25,94724		26,14324	25,91584		UCC1;EPDR1
29,12593	28,903		28,9914	28,83391		INTS1;DKFZP586J0619
NaN	NaN		NaN	NaN		GRID2IP
27,32783	27,17303		27,6811	27,01287		SUN1;UNC84A
NaN	NaN		NaN	NaN		KAT6A;MYST3
27,06271	27,32526		NaN	27,19657		TRAPPC13
31,61903	31,53519		31,66848	31,59784		CNOT1
NaN	NaN		NaN	NaN		CNOT1
27,52133	27,27808		NaN	NaN		FCRLA
26,53522	26,40701		26,41926	26,45304		CCDC88B
27,00571	NaN		NaN	NaN		MAP1LC3B;MAP1LC3B2
28,88198	28,62219		29,13756	29,01988		MRPL42
29,08068	29,19061		29,48947	29,55721		PGP
28,787	28,65997		28,71299	28,6634		C5orf51
26,73014	26,76522		26,52031	26,8903		RCCD1
32,84892	32,73615		32,79589	32,80697		HCFC1
27,19676	27,6147		26,43261	26,41511		TSPAN14

Supplemental Table 9 – Upper part of Nalm-6 & SEM secretome, see Suppl. Table 9

SEM_sgADAM10	SEM_sgADAM10	SEM_sgADAM10	…	SEM_sgNT	SEM_sgNT	SEM_sgNT	…	Gene names
Log 2 protein intensity	Log 2 protein intensity2	Log 2 protein intensity3		Log 2 protein intensity5	Log 2 protein intensity6	Log 2 protein intensity7		
21.97369	22.35778	23.7684		23.70698	23.70056	23.8253		UBE2I
24.63384	24.7747	24.34922		24.69519	25.11188	25.00804		APOC3
23.87905	23.75113	24.81416		23.58486	23.55975	22.96964		XRCC6
23.84842	24.37451	24.56831		23.56959	22.82842	23.68509		PSAP
24.37471	23.96473	24.50736		23.26714	23.86467	23.64031		PGD
28.75735	29.29391	29.54845		28.54049	28.36545	28.44186		HIST1H2BH;HIST1H2BN;HIST1H2BC;…
23.37265	25.43428	23.77324		23.09851	22.60008	22.72952		EEF1G
23.70577	23.88735	24.4789		22.63322	24.12728	24.25019		HNRNPC;HNRNPCL1
22.73357	25.11056	24.11004		24.81953	25.62363	24.90311		CLSTN1
24.95505	25.56049	26.3397		25.55289	26.10612	25.32985		RAN
24.20113	25.37587	24.61549		24.63489	24.9354	24.52981		MYL6
24.28071	25.23371	25.3654		24.12335	24.71261	25.42406		RCSD1
26.36678	27.04906	27.55735		26.3987	25.75239	26.79228		PTMA
23.23305	23.45676	23.35528		23.33101	23.09312	23.08145		RPS3A
23.19983	23.81118	23.84441		23.66112	23.77475	23.87652		RPL14
24.86604	23.88482	24.82813		24.46874	24.91766	23.22045		CD44
27.21391	27.27258	26.85436		27.15605	27.409	27.33492		LTF
22.52553	22.75678	23.03508		23.07276	23.15726	23.79313		FLT3
23.08034	24.02732	23.98135		23.76111	23.49192	24.46656		THBS4
26.12082	26.42203	26.91216		25.82677	26.07085	26.23983		GDI2
22.8791	22.92047	23.30382		24.80907	25.71243	24.7604		APP
24.61244	24.82886	25.22964		25.02741	24.58101	24.57704		C1QTNF3;C1QTNF3-AMACR
24.17722	24.14313	24.27555		24.16454	23.06033	23.1878		SPARC
22.09138	23.74216	23.83218		23.11429	24.10005	23.84575		SLC3A2
29.30066	29.65226	29.5941		29.61189	29.93579	29.76305		B2M
25.04444	25.38192	25.74699		23.4665	22.98336	24.05449		DDX39B;hCG_2005638
24.63262	25.41174	25.89296		25.28503	24.50519	24.15576		AK2
25.0032	24.96058	25.71495		25.25929	24.56192	24.17333		HNRNPA1;HNRNPA1L2
25.96431	25.74265	25.40734		25.08537	25.59255	25.50573		FN1
23.29348	24.02158	24.34523		23.08229	22.39473	22.52504		GALNT2
23.95615	24.38831	23.85405		24.55139	24.41577	24.94668		PSMA6
23.24371	23.31672	23.24942		23.44859	23.2369	23.22457		MST1
24.79566	27.33091	25.89294		24.77696	22.94238	25.3809		PDIA3
22.66505	22.40603	22.46441		22.87275	22.99312	23.07035		TGFBI
23.636	22.76985	23.71764		23.24376	23.06622	24.57625		HNRNPH1
24.37981	24.36526	24.66199		23.95828	24.45473	24.12629		CALM1;CALM2;CALM3
23.65141	24.42854	24.78606		24.07447	24.41493	24.60213		SUMO2;SUMO4
26.0793	25.81284	26.51947		25.78387	25.8779	25.90995		NUCB2;Nucb2
22.54861	24.27313	23.5099		23.74376	24.25674	23.61425		RPL17
22.0047	24.31717	22.22022		22.43284	21.96617	22.09832		AP2B1
24.32828	23.23425	22.93292		23.26757	22.41751	22.54772		RCN3
23.95607	23.09373	24.59161		22.96721	25.25703	21.76236		CLIC1
21.74958	23.26939	25.97053		24.36893	24.21215	23.8467		QSOX1
23.87019	23.23616	22.81552		26.36708	25.92536	25.59639		ADAM10
23.58532	23.43911	23.29011		24.44285	23.38298	22.71988		XPO1
24.88218	23.87951	24.38561		23.78547	24.27576	24.16277		ARPC2
23.29735	23.95403	24.42956		23.47395	23.67196	23.5667		COCH
26.63506	27.20937	27.12288		26.97705	26.95416	26.57715		ACTN4

Supplemental Table 10 – Upper part of PDX transcriptome, see Suppl. Table 10

	ALL199_Ctrl1	ALL199_Ctrl2	ALL265_Ctrl1	ALL265_Ctrl2	ALL199_KO2	ALL265_KO1	ALL265_KO2
ENSG00000179988	2.754707676	3.013954072	3.726722361	3.453735576	-0.212335252	0.522741296	0.987611541
ENSG00000146966	0.639230458	0.692025977	1.404794266	-0.005696042	3.488104467	3.692666297	3.794966463
ENSG00000139354	3.80915546	4.151457596	5.311684862	5.123586975	2.595019671	0.522741296	3.794966463
ENSG00000160360	4.668977802	3.861950978	4.864225885	5.123586975	5.977489307	6.50002122	5.511173497
ENSG00000083097	3.912248953	4.151457596	4.574719268	4.638160148	1.372627249	3.692666297	3.309539635
ENSG00000261645	4.008464268	3.499380899	4.574719268	2.80165888	-0.212335252	2.844669391	2.572574041
ENSG00000215845	2.754707676	2.276988478	3.726722361	2.80165888	-0.212335252	0.522741296	0.987611541
ENSG00000148400	4.480532712	5.335882167	4.574719268	4.517865914	6.054451289	5.380722291	6.03200566
ENSG00000108666	3.80915546	3.861950978	2.989756767	3.694743676	5.145216753	5.166597486	5.075074382
ENSG00000165490	3.577829914	3.013954072	4.212149188	2.316232053	-0.212335252	0.522741296	2.572574041
ENSG00000256947	-0.945732042	0.692025977	1.404794266	-0.005696042	2.595019671	2.844669391	3.794966463
ENSG00000100083	5.497211454	5.821308994	3.726722361	5.609013802	6.387577591	6.35563131	6.479464637
ENSG00000232072	3.446585381	3.861950978	2.989756767	3.164228959	-0.212335252	2.844669391	0.987611541
ENSG00000000460	4.183550975	3.861950978	3.726722361	4.74919146	1.372627249	3.330096218	4.157536542
ENSG00000169242	0.639230458	2.276988478	4.212149188	4.386621381	4.916947765	5.277628798	3.309539635
ENSG00000188375	4.608856809	4.779488818	5.492257108	5.876947007	3.488104467	4.429631892	4.688051259
ENSG00000280239	2.754707676	3.013954072	2.989756767	3.453735576	-0.212335252	2.107703797	0.987611541
ENSG00000277117	3.698124148	4.598916573	5.311684862	5.203757323	2.109592843	4.915058719	3.309539635
ENSG00000283674	5.899758009	5.901479343	1.404794266	2.316232053	6.295459389	4.223181014	4.894502136
ENSG00000226330	2.224192959	2.276988478	2.989756767	3.164228959	4.311226705	4.610204137	4.157536542
ENSG00000096092	3.302195471	3.013954072	4.864225885	5.279706177	1.372627249	3.330096218	4.447043159
ENSG00000147889	3.698124148	3.013954072	6.762346271	6.788719824	1.372627249	6.405384345	6.715531995
ENSG00000103381	4.339670177	4.598916573	5.311684862	4.948500268	3.247096367	3.330096218	3.794966463
ENSG00000107331	3.912248953	2.276988478	3.726722361	3.453735576	5.145216753	5.046303252	4.447043159
ENSG00000143315	0.639230458	0.692025977	2.989756767	3.164228959	4.035592262	4.429631892	3.309539635
ENSG00000133678	0.639230458	3.013954072	3.726722361	3.453735576	3.87512759	4.915058719	4.157536542
ENSG00000197265	5.076635771	3.861950978	4.574719268	5.038698077	2.595019671	4.610204137	3.794966463
ENSG00000105726	3.698124148	3.861950978	3.726722361	2.80165888	4.997118114	4.223181014	5.235539054
ENSG00000073584	2.754707676	0.692025977	2.989756767	3.453735576	3.87512759	5.166597486	4.894502136
ENSG00000267968	2.224192959	2.276988478	2.989756767	2.80165888	-0.212335252	0.522741296	0.987611541
ENSG00000237413	1.376196053	3.013954072	2.989756767	3.164228959	-0.212335252	0.522741296	0.987611541
ENSG00000183484	4.263721323	4.598916573	4.212149188	3.164228959	5.279517845	5.166597486	4.894502136
ENSG00000122778	3.302195471	3.013954072	4.864225885	3.694743676	-0.212335252	3.692666297	4.157536542
ENSG00000114638	3.141730799	2.276988478	1.404794266	-0.005696042	4.179982171	3.330096218	3.309539635
ENSG00000107742	0.639230458	0.692025977	1.404794266	-0.005696042	2.109592843	3.330096218	3.794966463
ENSG00000127989	3.141730799	3.861950978	4.574719268	3.901194553	1.372627249	2.844669391	2.572574041
ENSG00000285533	1.376196053	0.692025977	1.404794266	-0.005696042	2.595019671	2.844669391	4.157536542
ENSG00000166897	-0.945732042	0.692025977	1.404794266	2.316232053	3.488104467	2.844669391	3.309539635
ENSG00000172247	3.446585381	3.499380899	3.726722361	3.694743676	5.3422536	4.223181014	4.688051259
ENSG00000165730	2.754707676	0.692025977	1.404794266	2.80165888	3.87512759	4.770668809	3.794966463
ENSG00000156253	3.141730799	3.013954072	2.989756767	3.164228959	-0.212335252	2.844669391	0.987611541
ENSG00000276850	2.513699576	2.276988478	2.989756767	2.316232053	-0.212335252	0.522741296	0.987611541
ENSG00000244486	1.86162288	2.276988478	2.989756767	2.80165888	-0.212335252	0.522741296	0.987611541
ENSG00000274536	2.961158553	3.013954072	2.989756767	1.579266458	-0.212335252	0.522741296	0.987611541
ENSG00000138780	3.577829914	3.499380899	3.726722361	4.852284953	1.372627249	3.692666297	3.309539635
ENSG00000154721	0.639230458	2.276988478	1.404794266	1.579266458	2.595019671	4.915058719	3.309539635
ENSG00000246662	4.411819962	2.276988478	4.212149188	3.164228959	-0.212335252	3.330096218	3.794966463
ENSG00000136925	-0.945732042	0.692025977	1.404794266	-0.005696042	2.595019671	2.107703797	3.309539635
ENSG00000168476	4.263721323	3.499380899	5.492257108	5.123586975	2.95758975	3.692666297	3.794966463

Supplemental Table 11 – Upper part of PDX proteome, see Suppl. Table 11

ALL-199

[9]	[10]	[11]		[13]	[14]	[15]		
PDXALL199_sgNT4_D4_1	PDXALL199_sgNT3_D3_1	PDXALL199_sgNT2_D2_1		PDXALL199_ADAM10sg5_1_E1_1	PDXALL199_ADAM10sg3_2_D8_1	PDXALL199_ADAM10sg3_1_D7_1		Gene name
#!{Type}E	E	E		E	E	E		T
#!{C:Group1}ALL199_sgNT	ALL199_sgNT	ALL199_sgNT		ALL199_ADAM10sg	ALL199_ADAM10sg	ALL199_ADAM10sg		
#!{C:Group2}199_sgNT4_D4_1	199_sgNT3_D3_1	199_sgNT2_D2_1		199_ADAM10sg5_1_E1_1	199_ADAM10sg3_2_D8_1	199_ADAM10sg3_1_D7_1		
#!{C:WTvsKO} **199_sgNT**	**199_sgNT**	**199_sgNT**		**199_ADAM10**	**199_ADAM10**	**199_ADAM10**		
1.001.806	1.011.163	9.671.243		9.508.953	9.582.053	9.456.795		TMED7;TMED7-TICAM2
6.149.461	6.639.676	6.741.212		7.042.643	8.009.857	7.554.948		ATP1A3
7.563.487	7.362.911	8.241.592		8.129.598	8.384.026	8.504.235		NBAS
8.350.802	8.163.842	8.593.428		8.709.126	9.065.684	9.440.663		SBNO1
7.367.469	715.262	7.824.297		8.061.127	8.016.887	8.125.627		GTPBP10
9.372.147	9.122.854	9.088.761		9.579.765	9.793.821	9.882.901		CNOT1
8.119.548	8.028.555	8.515.277		8.723.104	8.721.404	8.913.559		PGP
1.008.909	1.168.353	9.362.585		7.896.412	8.507.013	8.694.868		
6.888.989	7.120.026	7.710.824		7.736.879	8.047.368	7.636.267		STX16;STX16-NPEPL1
1.006.096	9.857.245	1.006.756		1.054.965	1.036.975	1.036.307		KIF2A
1.479.286	1.469.859	1.380.887		1.329.535	1.296.719	1.328.807		PDLIM1
9.255.605	8.891.788	9.279.179		9.411.809	9.666.834	9.489.795		GTPBP1
9.751.473	989.103	1.011.924		9.943.788	1.025.751	1.021.533		AP3B1
1.005.201	9.754.685	9.898.282		1.054.717	1.061.563	1.062.075		DNM1L
7.208.771	7.219.938	8.103.641	...	8.044.245	8.734.176	9.191.479	...	RTCA
9.061.785	8.940.099	8.148.528		2.615.312	311.668	2.919.789		BTN3A1;BTN3A3
1.193.121	1.210.129	1.176.632		1.201.599	1.221.639	1.207.325		NDUFA4
9.464.589	9.307.855	8.869.783		1.024.253	9.868.064	9.844.872		PES1
9.393.362	9.683.137	9.941.689		1.068.953	106.096	1.004.309		NOP56
1.385.611	1.390.465	1.318.418		1.274.308	1.312.025	1.294.926		DDX3X
1.035.305	1.036.442	9.408.637		9.563.851	9.042.272	9.209.166		RNASET2
852.304	8.332.319	9.635.457		9.502.911	9.745.842	1.030.918		KPNA4
9.994.181	9.942.956	9.483.191		9.508.183	9.175.575	9.587.019		MAN2B1
9.319.633	9.406.611	8.778.732		8.640.972	8.541.338	7.780.745		UBE2C
920.233	9.320.528	9.313.633		9.702.359	9.882.587	1.025.801		TXNDC9
6.998.336	6.790.384	7.164.915		7.723.635	7.691.453	8.127.549		CYB561D2
8.191.623	7.859.099	8.396.555		8.938.165	8.994.308	8.487.145		AP3D1
6.536.903	653.207	7.531.904		8.103.566	8.041.673	8.150.712		CHD1
8.563.388	8.560.874	8.339.785		8.017.783	7.926.603	7.996.283		TOR1A
7.343.154	7.533.006	7.846.326		6.769.946	6.901.292	6.353.003		ADAM10
5.137.124	2.902.491	6.239.569		5.873.344	6.925.935	6.871.155		IMPA2
8.944.592	8.706.138	8.888.735		9.239.067	9.191.463	9.467.463		CHEK1
1.039.406	103.966	1.020.578		1.038.452	9.858.027	9.374.342		TCERG1
1.143.819	1.136.817	1.135.067		1.064.457	1.058.846	1.071.931		PSMA7
9.824.212	9.948.473	8.909.498		8.430.671	8.380.561	8.216.546		UQCRQ
7.758.135	7.892.669	8.344.372		8.165.895	850.776	8.461.235		PLXNB2
1.023.441	1.055.487	1.040.365		9.646.143	9.637.118	1.014.189		U2SURP
1.304.334	1.295.679	1.331.477		1.244.812	1.286.618	130.628		ARPC2
8.399.593	8.345.587	8.915.689		734.843	7.763.858	7.752.886		POLR1C
7.874.833	1.612.227	7.972.251		291.519	2.429.004	3.242.785		PLSCR1
9.552.673	9.705.145	9.288.818		9.249.681	9.075.508	9.061.498		SURF4
1.050.956	1.052.053	1.067.506		1.077.584	1.073.278	1.093.284		OGT
738.851	7.442.133	6.476.588		6.158.623	5.099.563	5.140.492		NUPL2

Supplemental Table 12 – Upper part of competitive LDTA, see Suppl. Table 12

Mouse sample	ALL-199 cell number	human	BFP+ = KO	T-Sapphire+ = Ctrl
20210408 - ALL199 take down_74072-81 10 50K P6_015.fcs	10	3,03%	0%	99,90%
20210408 - ALL199 take down_74073-82 10 100K P1_016.fcs		0%	0%	0%
20210408 - ALL199 take down_74074-83 10 100K P1_017.fcs		0%	0%	0%
20210408 - ALL199 take down_74075-84 20 100K P1_018.fcs	20	0%	0%	0%
20210408 - ALL199 take down_74076-85 20 100K P1_019.fcs		9,42E-06	0%	0%
20210408 - ALL199 take down_74077-86 20 100K P1_020.fcs		0%	0%	0%
20210408 - ALL199 take down_74078-87 20 50K P6_021.fcs		11,10%	0%	99,80%
20210408 - ALL199 take down_74032-41 40 100K P1_010.fcs	40	0%	0%	0%
20210408 - ALL199 take down_74079-88 40 50K P6_022.fcs		7,62%	99,80%	0,06%
20210408 - ALL199 take down_74080-89 40 50K P6_023.fcs		0,55%	98,70%	0,12%
20210408 - ALL199 take down_74081-90 40 50K P6_024.fcs		80,80%	0%	100,00%
20210408 - ALL199 take down_74033-42 60 200K P1_011.fcs	60	0,36%	0%	100%
20210408 - ALL199 take down_74034-43 60 200K P1_012.fcs		0,75%	0,33%	99,60%
20210408 - ALL199 take down_74035-44 60 100K P1_013.fcs		0%	0%	0%
20210408 - ALL199 take down_74036-45 60 50K P6_014.fcs		34,70%	99,90%	3,07E-05
20210408 - ALL199 take down_74027-36 120 200K P1_005.fcs	120	0,04%	0%	95,70%
20210408 - ALL199 take down_74028-37 120 50K P6_006.fcs		15,90%	0%	100,00%
20210408 - ALL199 take down_74029-38 120 100K P1_007.fcs		0%	0%	0%
20210408 - ALL199 take down_74030-39 120 50K P6_008.fcs		25,30%	5,39%	94,50%
20210408 - ALL199 take down_74022-31 360 50K P6_002.fcs	360	44,20%	0%	100,00%
20210408 - ALL199 take down_74023-32 360 50K P6_003.fcs		66,60%	1,21%	98,80%
20210408 - ALL199 take down_74031-40 360 50K P6_009.fcs		25,20%	0,63%	99,30%
20210323 - ALL199 high 1080_74024-33 1080 50K P6_002.fcs	1080	5,55%	0,38%	99,60%
20210408 - ALL199 take down_74025-34 1080 50K P6_004.fcs		43,50%	0%	99,90%
20210312 - ALL199 pos ctrl_74026-35 20K_001.fcs	20000	52,10%	14,00%	85,60%

Legend: green = engraftment; red = no engraftment

Supplemental Figure S1

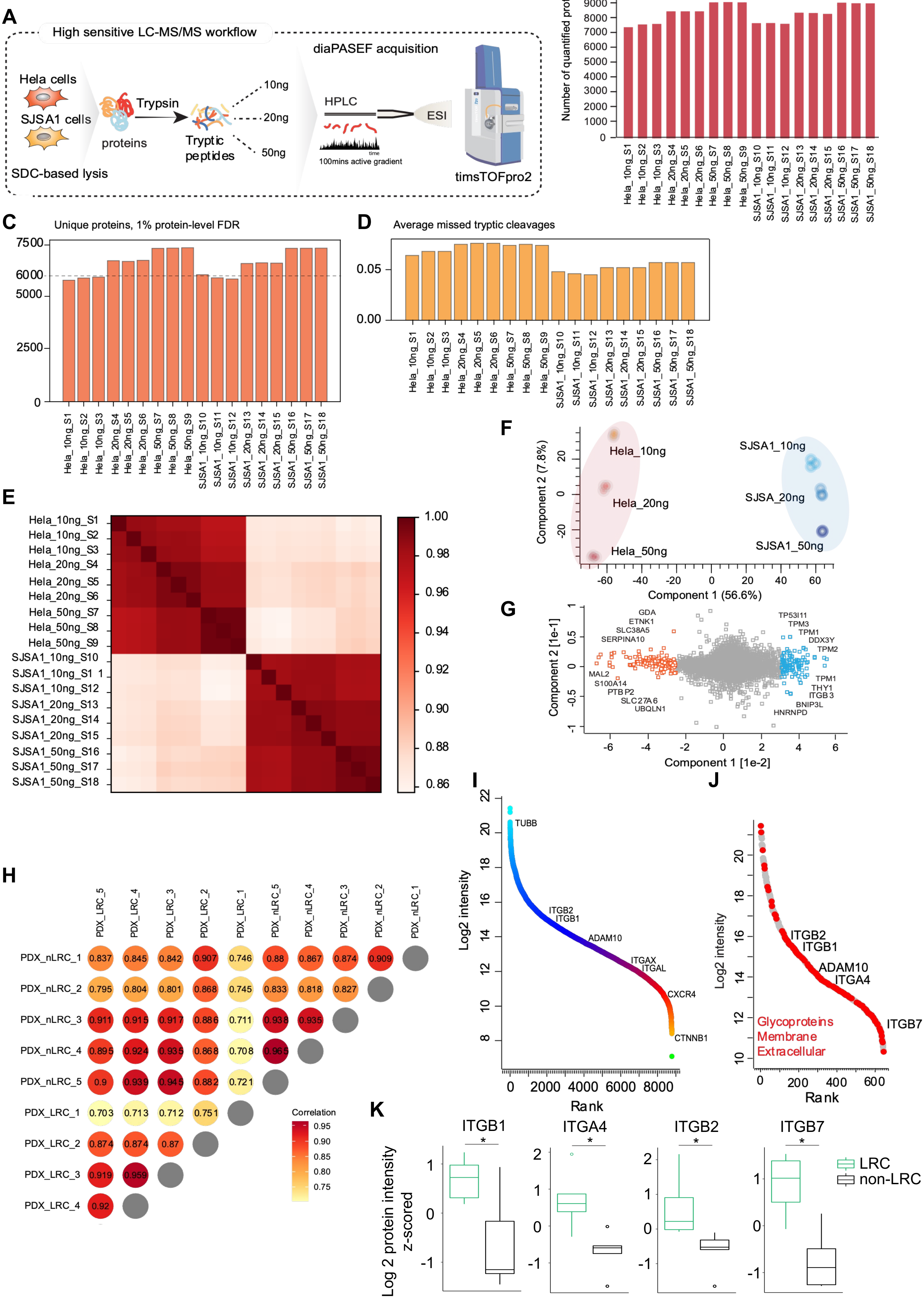

Supplemental Figure S1: Ultra-sensitive diaPASEF proteome workflow and proteomic characterization of slow-cycling PDX ALL cells. Data related to **Fig. 1**

A Scheme of ultra-sensitive proteome workflow. HeLa and SJSA1 cells were lysed in SDC-based lysis buffer, proteins were trypsin protease digested and peptides purified. The peptides were separated by liquid chromatography and measured utilizing diaPASEF acquisition mode in the Bruker TIMS pro-2 instrument.

B Bar plot showing number of quantified proteins in HeLa and SJSA1 cells at various peptide concentrations equivalent to 100-500 cells as technical triplicates.

C Bar plot showing number of unique proteins quantified in HeLa and SJSA1 cells at various peptide concentrations equivalent to 100-500 cells as technical triplicates.

D Bar plot showing average missed tryptic cleavages in all the samples measured.

E Pearson correlation of HeLa and SJSA1 proteome.

F Principal component analysis of the proteome samples.

G Scatter plot highlighting the protein components separating HeLa and SJSA1 cells in the PCA plot.

H Correlation matrix of all measured LRC and non-LRC samples based on Pearson correlation values.

I Protein rank plot showing the dynamic range and protein abundance of all quantified proteins in the proteome.

J Rank plot displaying all significantly regulated proteins in LRC and non-LRC comparison. Red highlighting indicates LRC regulated extracellular membrane proteins.

K Box plot representation of selected extracellular matrix and membrane proteins that are significantly upregulated in LRC compared to non-LRC. Z-scored log2 protein intensity is displayed for proteins with permutation-based FDR cut-off <0.05.

Supplemental Figure S2

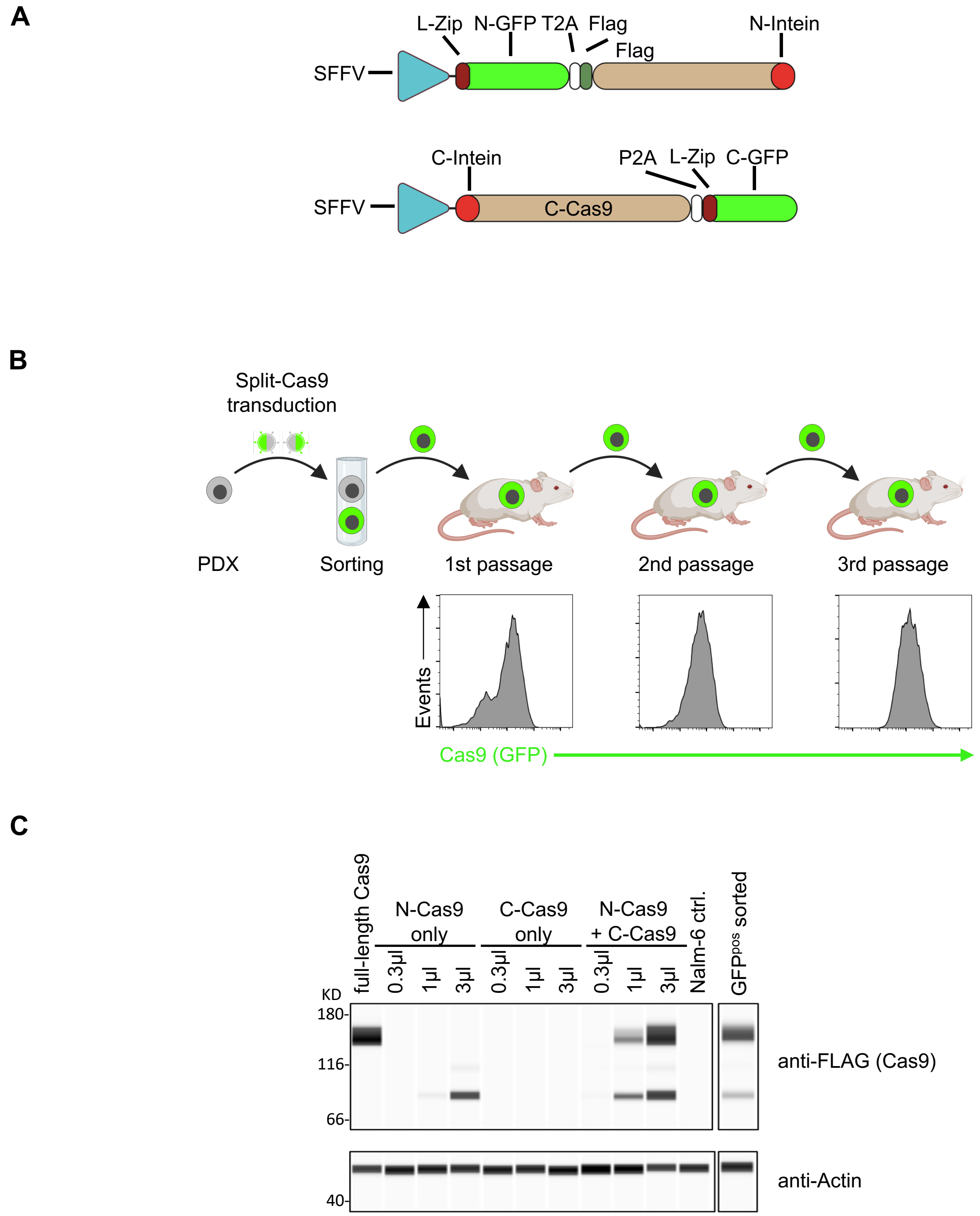

Supplemental Figure 2: Quality controls for generating Split-Cas9-transgenic PDX models. Data related to **Fig. 2A**

A Split-Cas9 expression vectors**:** Lentiviral vectors expressing the N-terminal part (upper vector) and C-terminal part (lower vector) of Cas9 and GFP, separated by a 2A self-cleaving peptide (T2A or P2A) under control of the spleen focus-forming virus (SFFV) promoter. N-terminal part of Cas9 is fused to a FLAG-tag; both parts of Cas9 are assembled by inteins, both parts of GFP by a leucine zipper. L-Zip, Leucine Zipper; C-Intein, C-terminal part of Intein; N-Intein, N-terminal part of Intein.

B Split-Cas9-GFP-transgenic PDX cells: PDX cells were lentivirally transduced with both split-Cas9-GFP expression vectors, transgenic cells enriched by flow cytometry for GFP-positive cells and injected into mice for amplification (upper panel). To quality control stable Cas9 expression over passaging, cells were analyzed for GFP expression after each passage and enrichment was repeated, if needed. Representative histograms indicating stable GFP expression over 3 passages in ALL-199 PDX cells are shown (lower panel).

C Protein detection by Simple Western (WES) immuno-assay of split-Cas9 in Nalm-6 cells transduced with split-Cas9 lentiviral constructs. Anti-Flag antibody was used to detect Cas9 (N-terminal) and anti-Actin antibody served as loading control. One proof- of-concept immuno-assay is shown.

Supplemental Figure S3

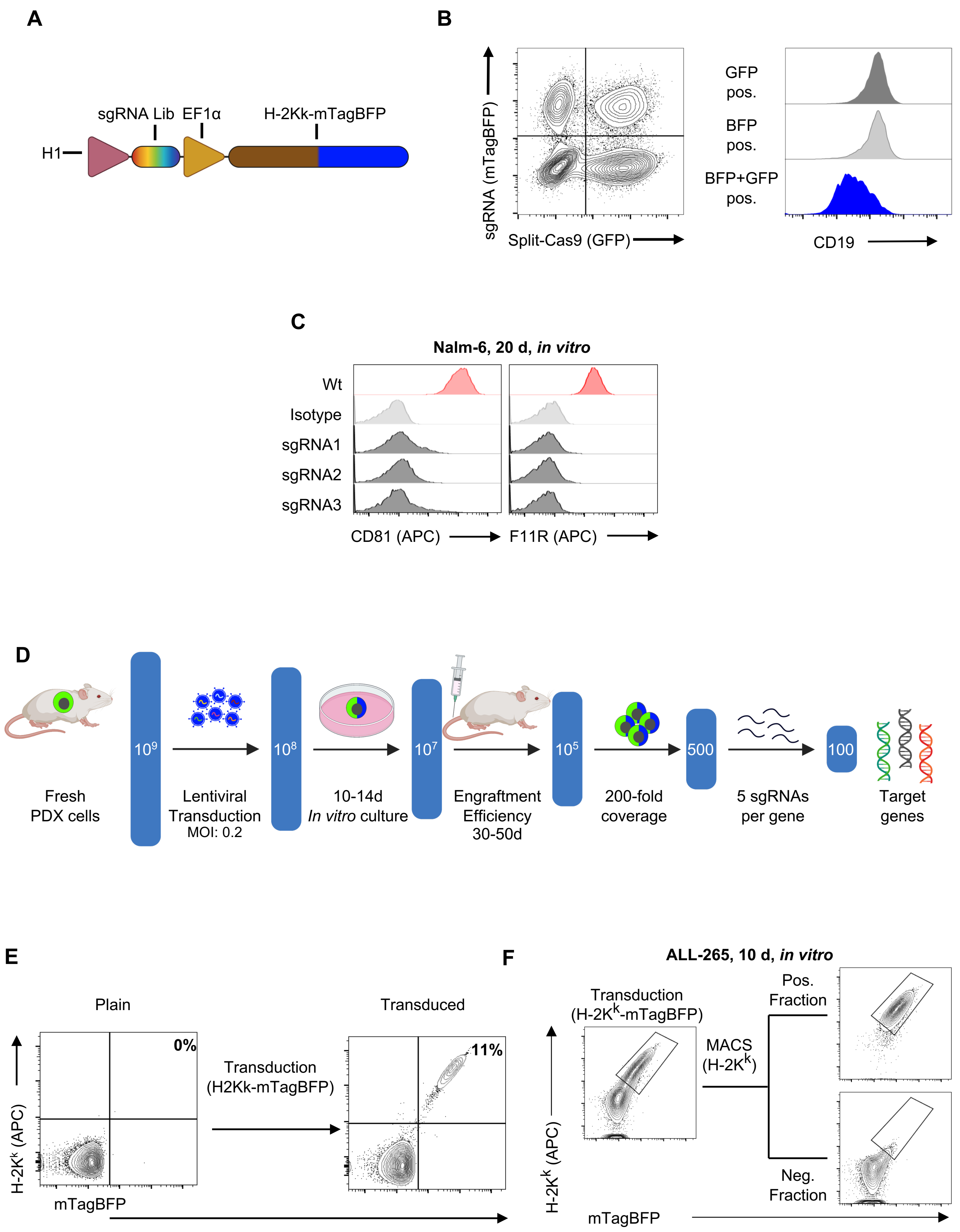

Supplemental Figure S3: Quality controls for generating CRISPR-Cas9 library-transgenic PDX models. Data related to **Fig. 2A**

A sgRNA expression vector: Lentiviral vector expressing library (Lib) sgRNAs from the H1 promoter and a marker protein consisting of the fluorochrome mTagBFP fused to H-2Kk (MHC-encoded class I molecule) under control of the elongation factor-1α (EF1α) short promoter for enrichment of transduced cells by flow cytometry and MACS.

BC Functionality of split-Cas9: **B** Nalm-6 cells transduced with split-Cas9 and sgRNA targeting CD19 were stained with a CD19-specific antibody and analyzed by flow cytometry for transgene expression (left) and CD19 expression in the different subfractions (right). One representative dot plot and histogram of two independent experiments is shown. **C** Split-Cas9-positive Nalm-6 cells were lentivirally transduced with 3 different sgRNAs targeting CD81 or F11R. Efficient knockout (KO) by each sgRNA was confirmed by flow cytometry compared to wildtype (Wt) and isotype controls.

D Workflow for performing a PDX *in vivo* dropout screen and cell numbers required for each step. The PDX individual sgRNA library size is determined by the PDX models' specific homing and engraftment efficiency. Blue bars are in logarithmic scale. MOI = Multiplicity of infection.

E Nalm-6 cells (Plain) were transduced with the sgRNA library co-expressing the H-2Kk-mTagBFP fusion marker. Cells were stained with APC-conjugated H-2Kk antibody and analyzed by flow cytometry. One representative contour plot of three independent experiments is shown.

F Representative MACS enrichment of sgRNA library-transduced ALL-265 PDX cells using anti-H-2Kk microbeads. Positive and negative fractions were stained with anti-H-2Kk-APC antibody and purity was analyzed by flow cytometry.

Supplemental Figure S4

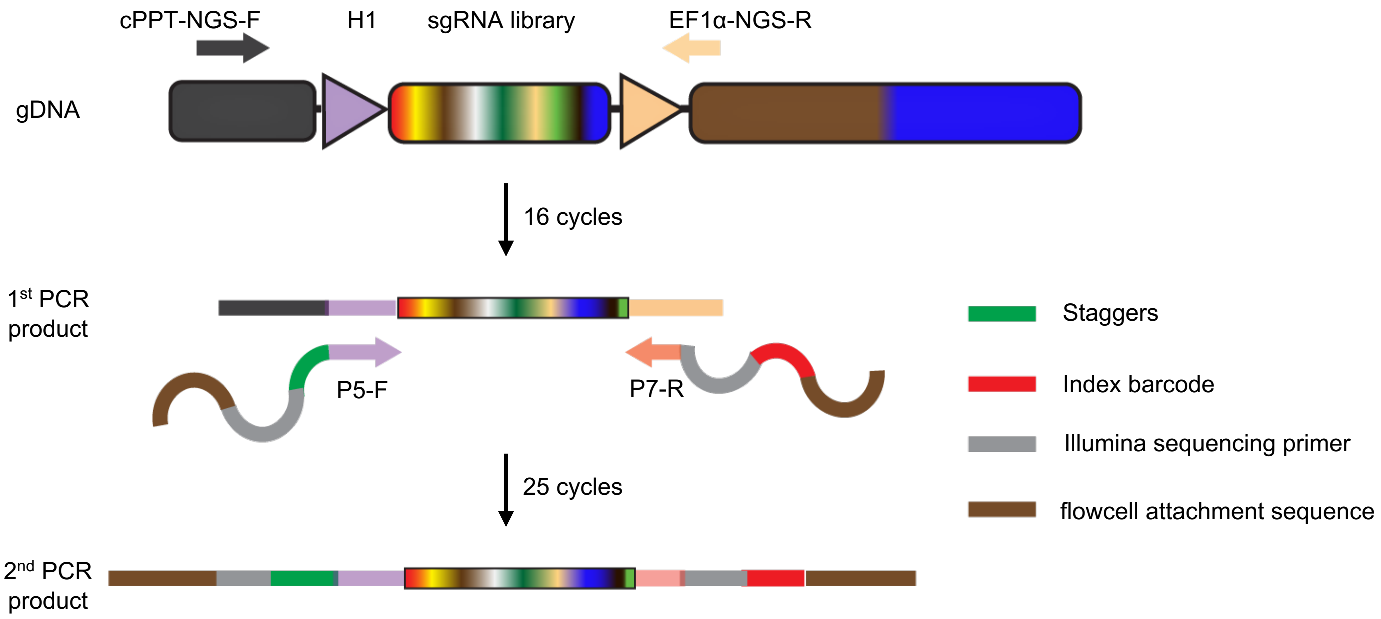

Supplemental Figure S4: Nested PCR. Data related to **Fig. 2A**

For amplification of sgRNA sequences integrated into the genome of human target cells, a nested PCR was established wherein the first PCR used primers outside the sgRNA sequence, recognizing sequences in cPPT and the EF1α promoter (primers cPPT-NGS-F and EF1α-NGS-R); the second PCR amplified an internal region of the first PCR, covering the sgRNA sequence and added staggers (primers P5-F) and index barcodes required for NGS sequencing (primers P7-R). List of primer sequences is provided in Supplemental Table 5.

Supplemental Figure S5

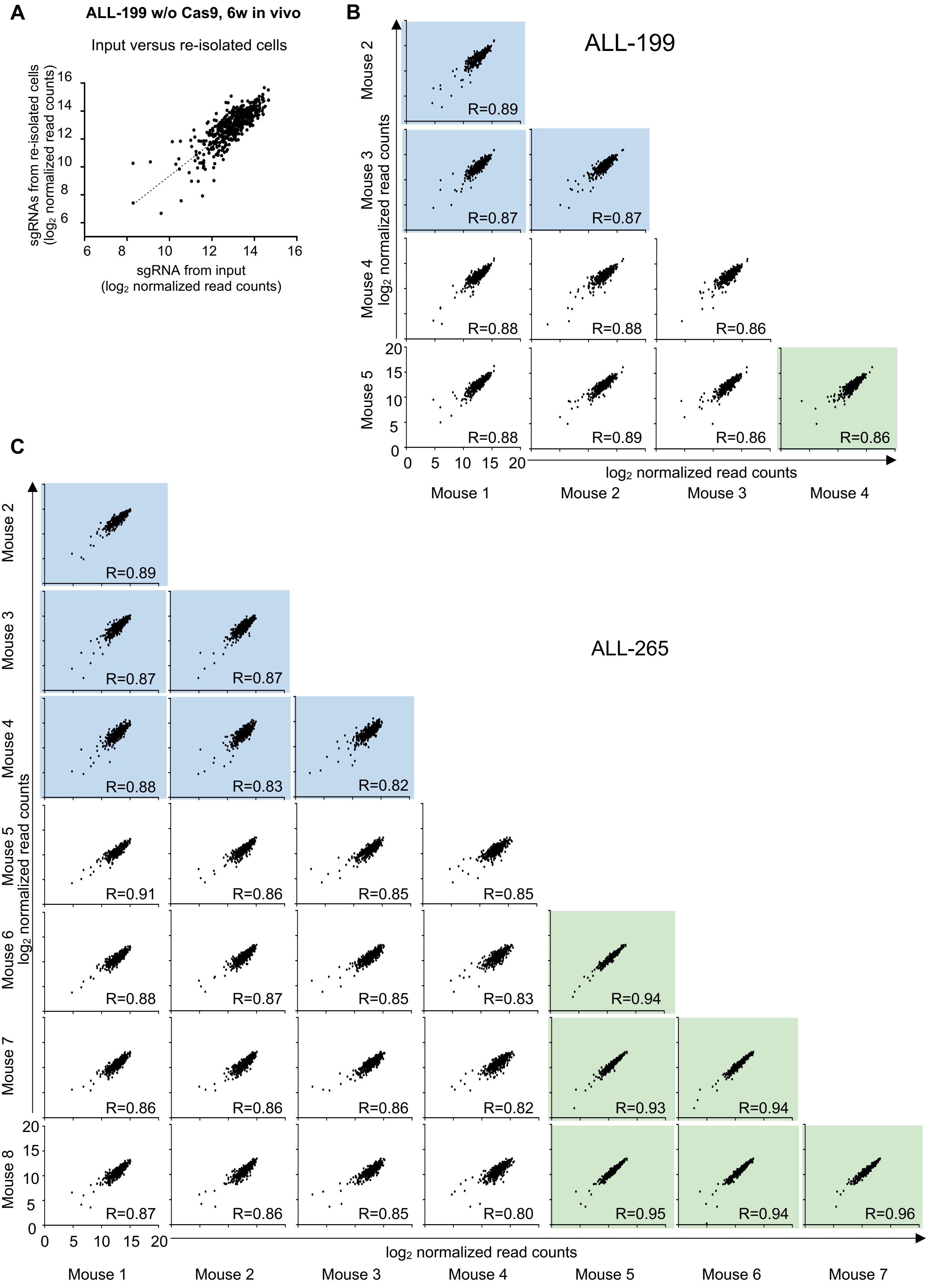

Supplemental Figure S5: Quality controls for the *in vivo* CRISPR dropout screens. Data related to **Figs. 2B, C**

A Split-Cas9-negative PDX ALL-199 cells were lentivirally transduced with the sgRNA library and injected into mice. Dot plot correlates sgRNA abundance between input at injection and output upon re-isolation from mice after six weeks of *in vivo* growth from a representative mouse.

BC The *in vivo* dropout screen was performed as depicted in **Figs. 2A** and **S3D** for ALL-199 (**B**, n=5 mice) and ALL-265 (**C**, n=8 mice). To quality control reliability, sgRNA abundances in output samples were correlated between different replicate mice. Blue and green boxes indicate comparison of replicate mice within two independent experiments. White boxes compare replicate mice between the two independent experiments.

Supplemental Figure S6

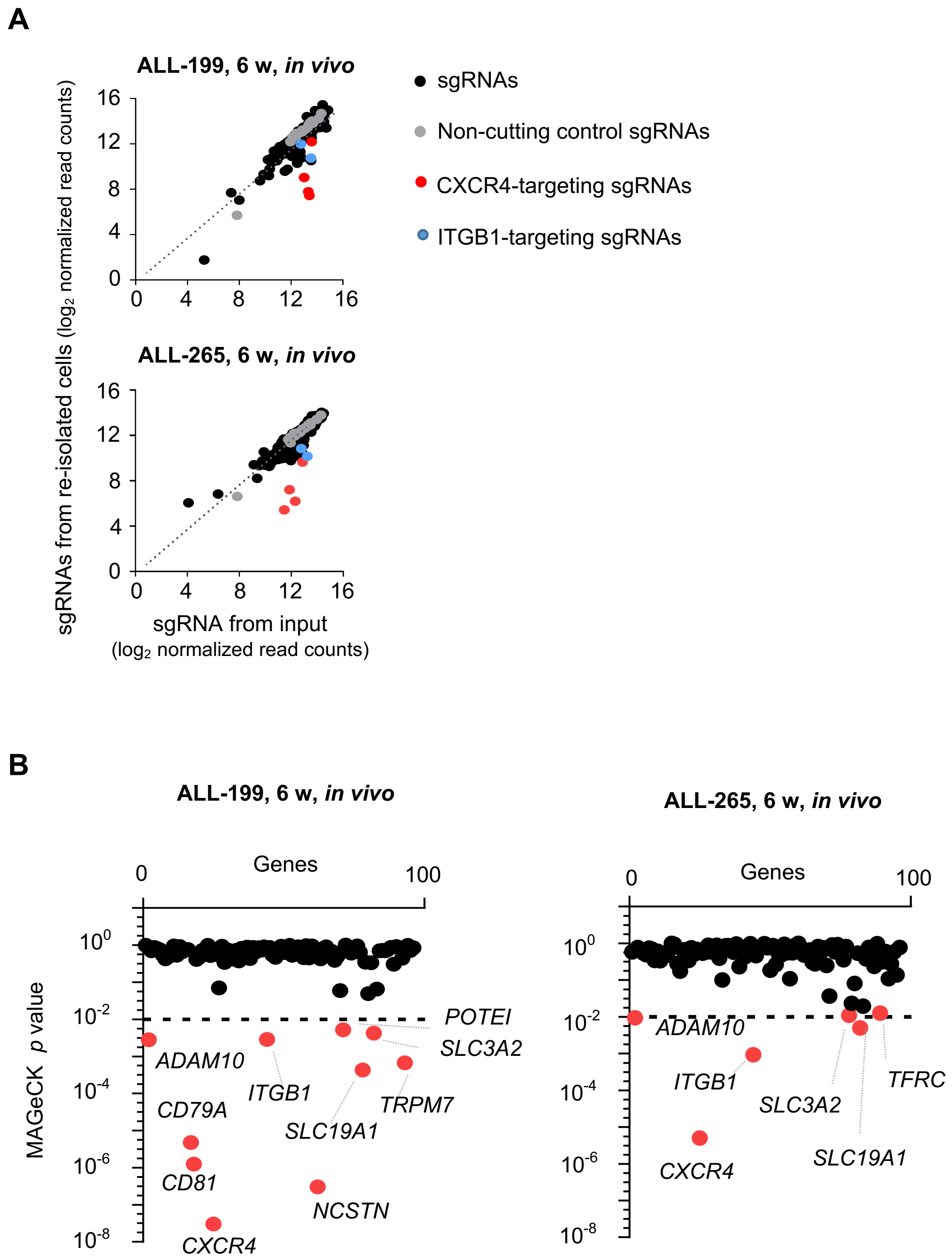

Supplemental Figure S6: Dropouts of the *in vivo* CRISPR screens in PDX ALL samples. Data related to **Fig. 2C**

A Correlation of sgRNA abundance between input and output in ALL-199 and ALL-265 cells after 6 weeks of *in vivo* growth; red dots represent the sgRNAs targeting CXCR4, blue dots represent the sgRNAs targeting ITGB1, grey dots represent the non-cutting control sgRNAs

B Significantly depleted genes (red dots) in ALL-199 and ALL-265 according to *p* values calculated by MAGeCK. Dotted lines indicate $p<0.01$ set as cutoff.

Supplemental Figure S7

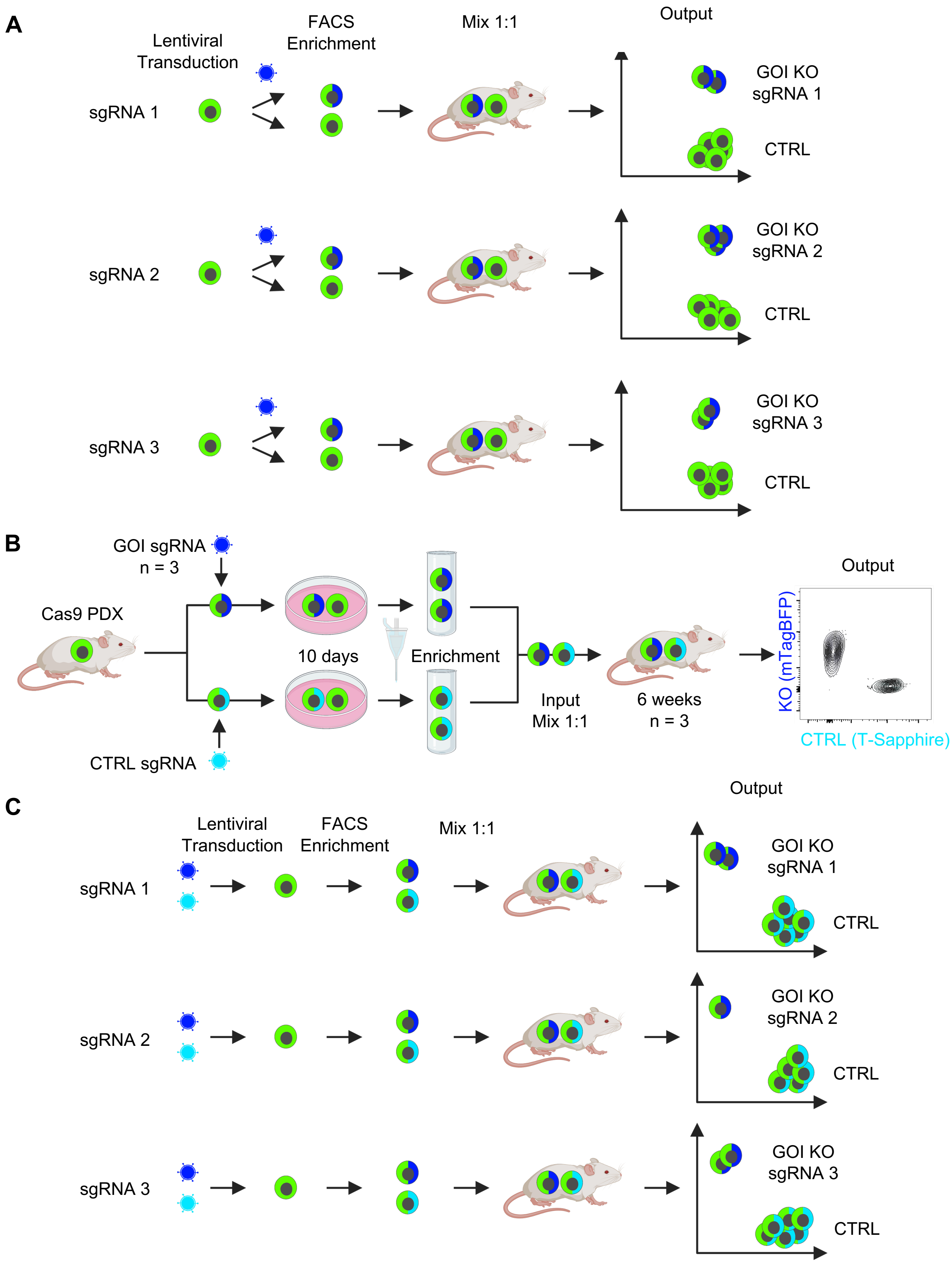

Supplemental Figure S7: *In vivo* competitive validation assay. Data related to **Figs. 2D-F, 3E, F**

Experiments were performed using three different sgRNAs per gene, each tested in a single mouse, and using as controls either untransduced cells (**A**) or cells transduced with a single construct expressing a control sgRNA together with T-Sapphire (**BC**).

Supplemental Figure S8

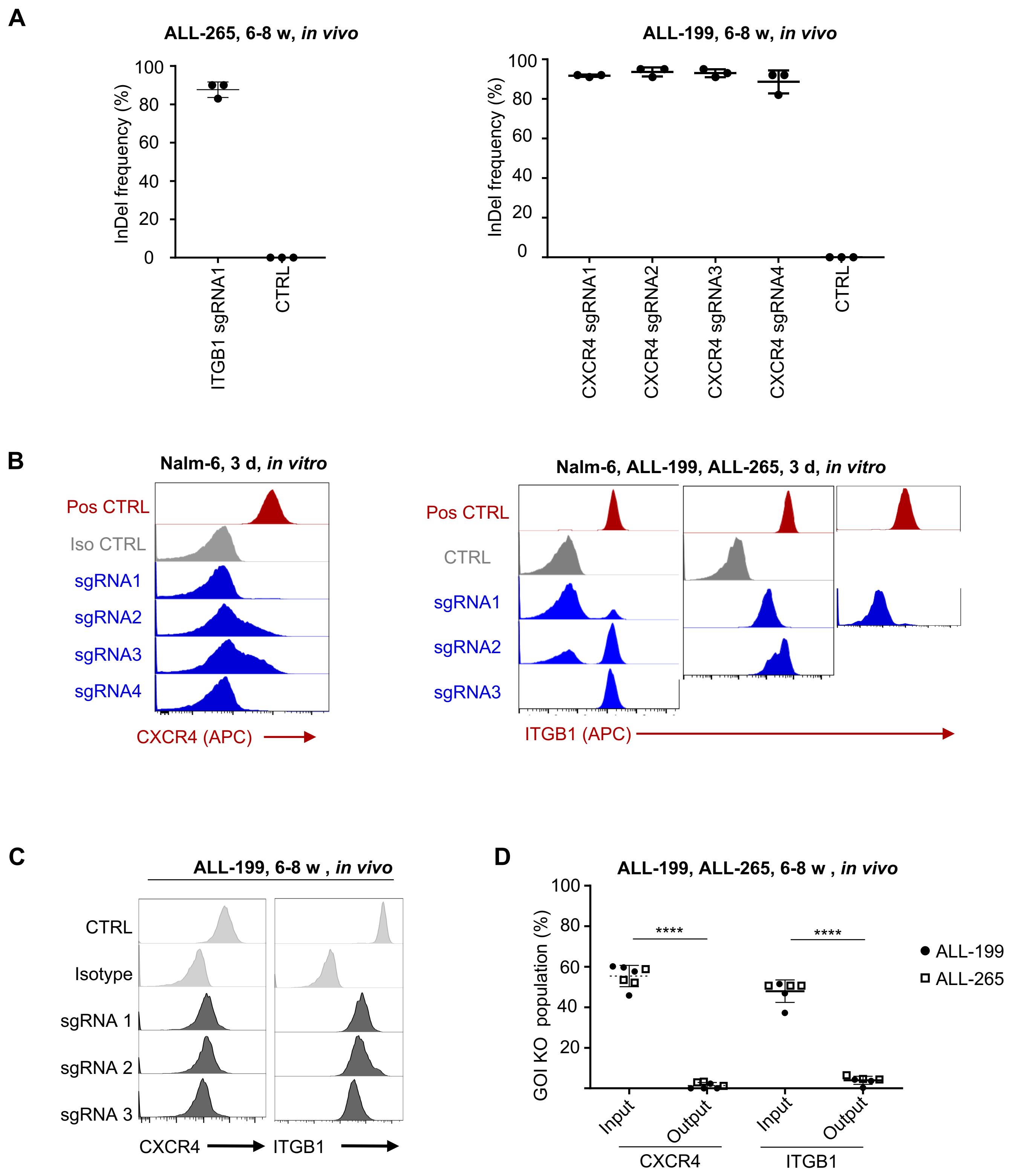

Supplemental Figure S8: Quality controls for *in vivo* validation assays for *CXCR4* and *ITGB1*. Data related to **Figs. 2D-F**

A Frequency of insertions and deletions (InDels) in TIDE analysis of output cells isolated from mice in ALL-265 cells with sgRNAs targeting *ITGB1* or ALL-199 cells with sgRNAs targeting *CXCR4.* Mean+/-SEM of three replicates is shown. Each dot represents one mouse and sgRNA (ALL-265) or one technical replicate of each of the mice and sgRNAs (ALL-199).

B Histograms depicting CXCR4 or ITGB1 protein expression three days after transduction of split-Cas9-positive Nalm-6, ALL-199 or ALL-265 cells with the indicated sgRNAs, targeting either *CXCR4* or *ITGB1* compared to controls. One histogram per sgRNA as well as the verum and isotype staining of the CTRL is shown.

C Output cells re-isolated from mice were stained with the respective antibodies and *CXCR4* and *ITGB1* expression was analyzed by flow cytometry. One histogram per sgRNA as well as the verum and isotype staining of the CTRL is shown.

D Identical data as in **Fig. 2F** but analyzed per gene by fusing data from both PDX samples. **** p<0.0001 by paired t-test.

Supplemental Figure S9

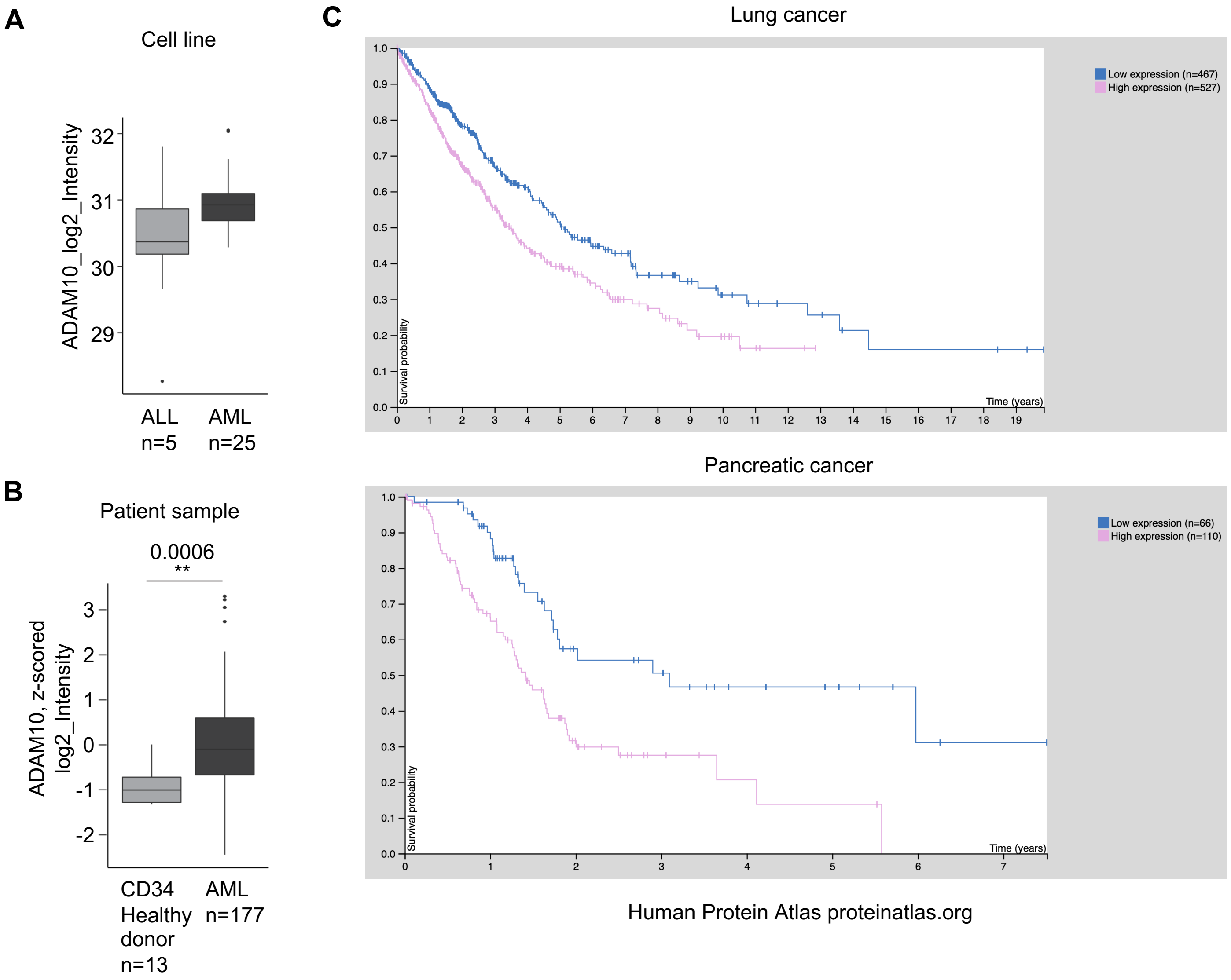

Supplemental Figure S9: ADAM10 expression in tumor cells and their impact on patient survival. Data related to **Figs. 3A-C**

A We had previously measured proteomes from ALL cell lines (n=5, triplicates, total of 15 samples) and AML cell lines (n=25, quadruplicates, total of 100 samples) (Jayavelu AK, 2022). Here, we analyzed these data for ADAM10 expression. Box plot displaying the log2 protein intensity of ADAM10.

B We had previously measured proteomes from 177 primary samples from AML patients and healthy donor CD34+ cells (n=13) (Jayavelu AK, 2022). Here, we analyzed these data for ADAM10 expression. Box plot displaying the z-scored log2 protein intensity of ADAM10.

C Kaplan-Meier curves correlating high or low ADAM10 expression with overall survival in lung and pancreatic cancer. Data retrieved from the Human Protein Atlas. Image available from v21.1.proteinatlas.org.

https://www.proteinatlas.org/ENSG00000137845-ADAM10/pathology

Supplemental Figure S10

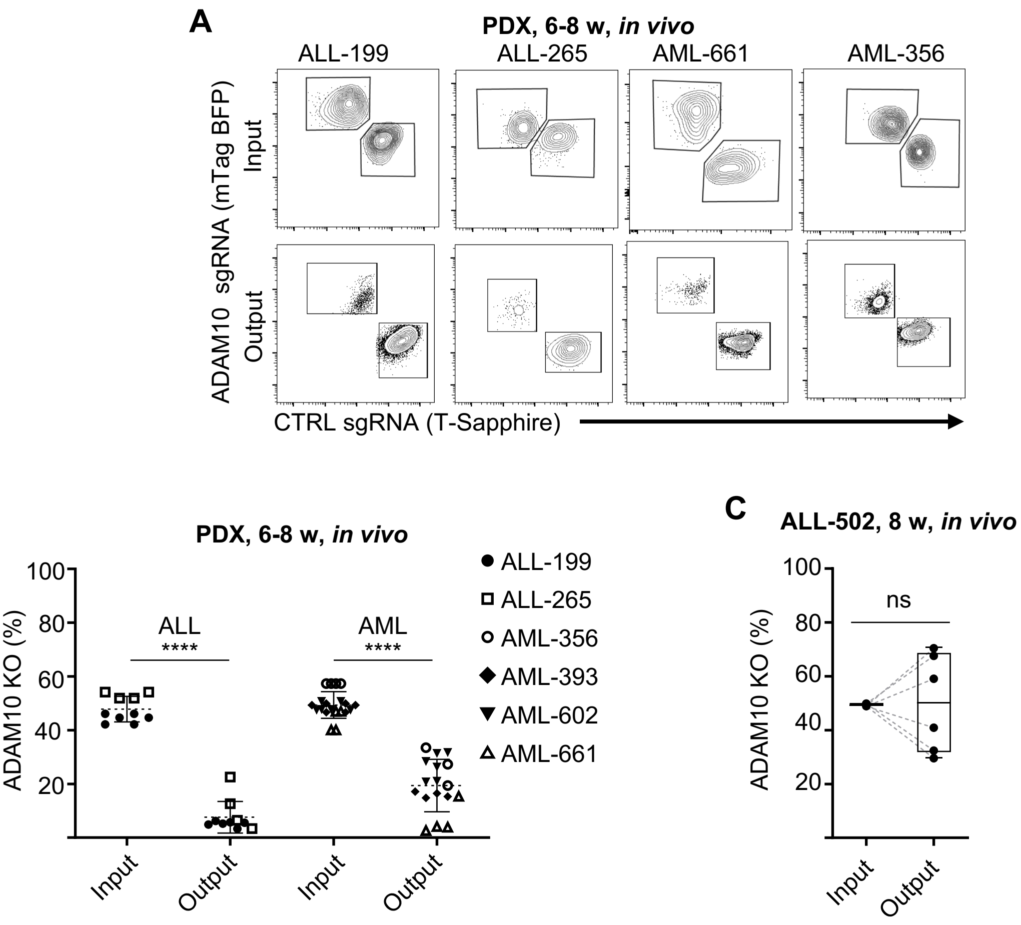

Supplemental Figure S10: ADAM10 *in vivo* validation assay. Data related to **Figs. 3E, F**

A Representative flow cytometry plots of *in vivo* competitive validation assay for ADAM10 in ALL-199, ALL-265, AML-661 and AML-356 from experiments described in **Fig. 3E**. Distribution of mTagBFP-positive KO cells and T-Sapphire-positive CTRL cells in the injection mixture (Input) and in re-isolated PDX cells after 6-8 weeks of *in vivo* growth (Output) is shown. Additional flow cytometry plots are available in the supplemental raw data file.

B Identical data as in **Fig. 3E** but analyzed per gene and by fusing data from all PDX samples. **** $p<0.0001$ by paired t-test.

C *In vivo* competitive validation assay for ADAM10 in ALL-502. Percentage of the KO populations in the injection mixture and in re-isolated ALL-502 (n=3) PDX cells following eight weeks of *in vivo* growth is depicted. Box indicates median, 25th and 75th percentile; whiskers indicate min/max. Each dot represents cells isolated from one organ (BM or spleen) of each mouse. Ns (not significant) by paired t-test.

Supplemental Figure S11

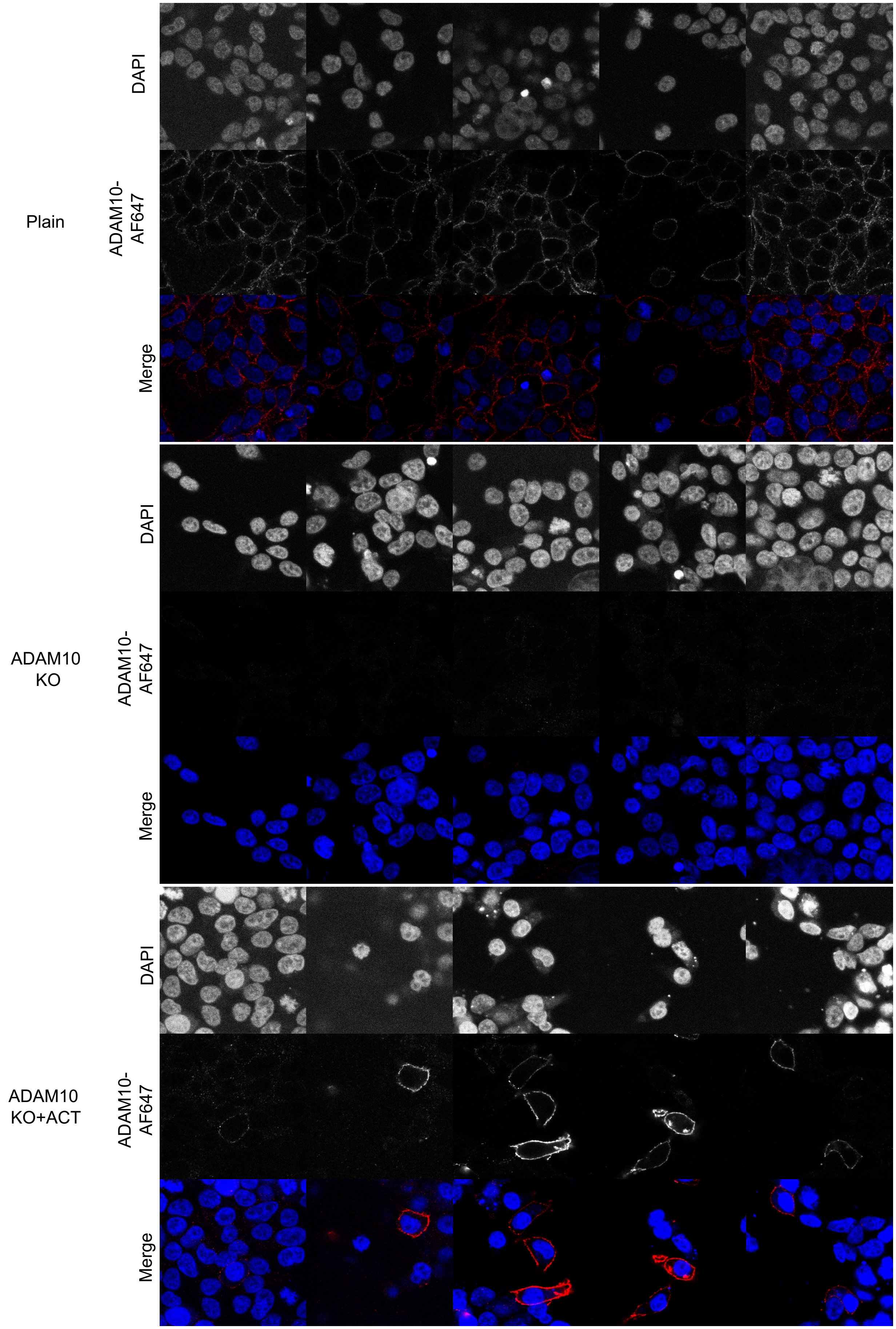

Supplemental Figure S11: ADAM10 reconstitution in HEK293T cells. Data related to **Fig. 4B**

Additional replicates of confocal microscopic analysis of ADAM10 surface protein expression as described in **Fig. 4B** are shown.

Supplemental Figure S12

A

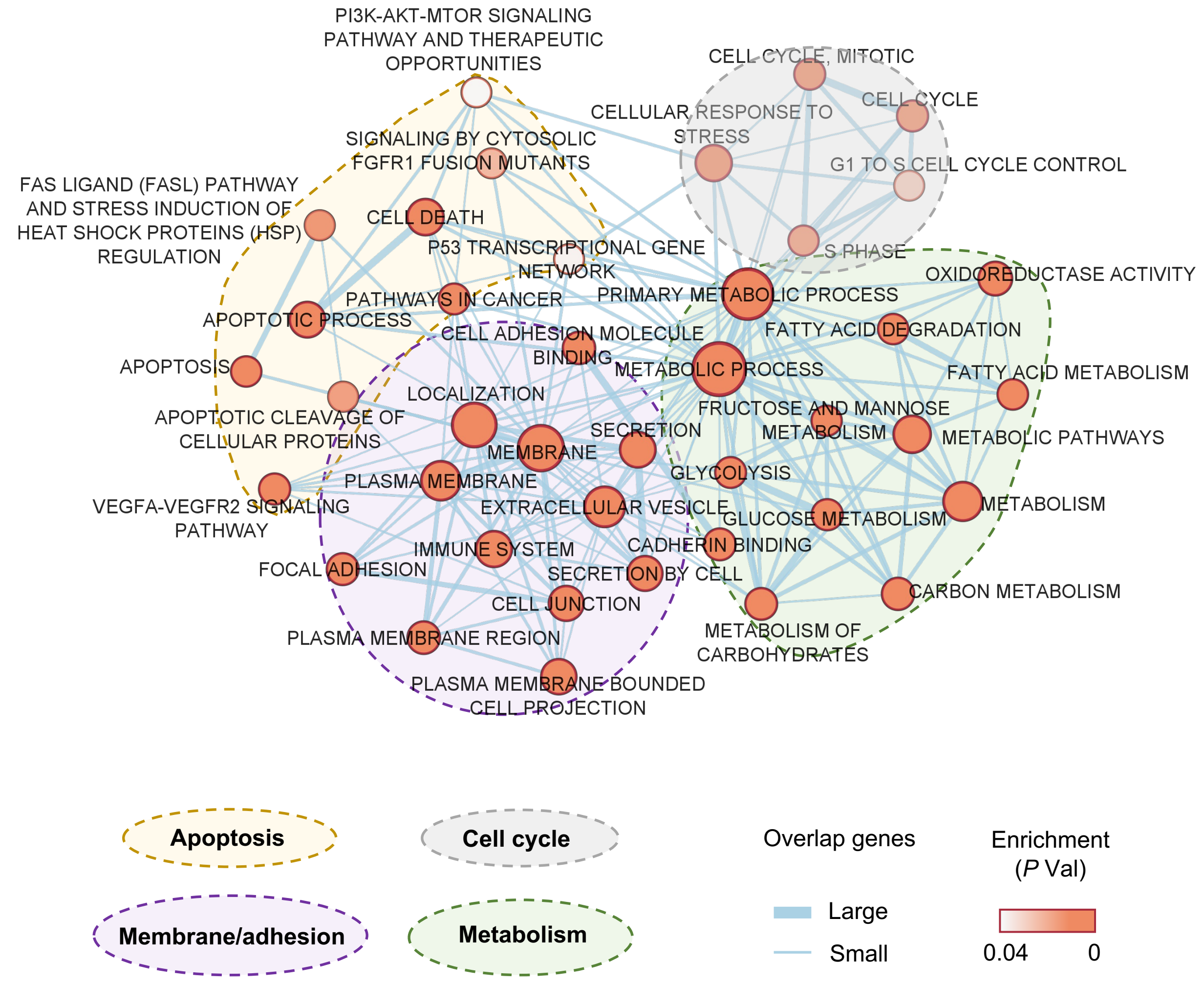

B

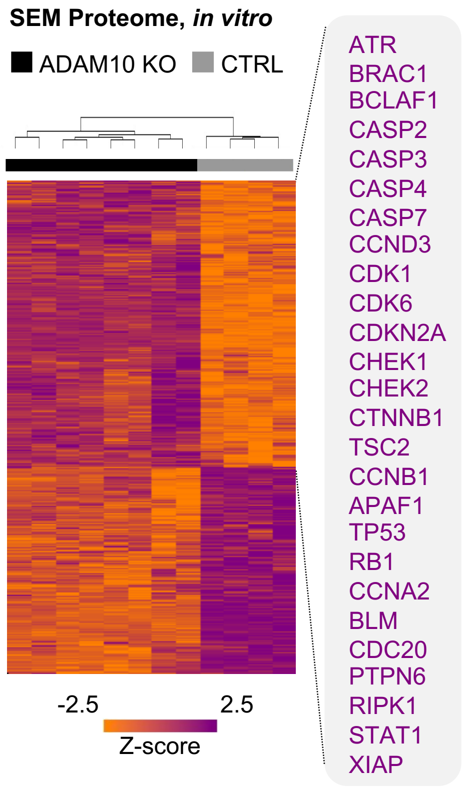

Supplemental Figure S12: Pathway enrichment results of ADAM10 KO proteome analyses in SEM cells. Data related to **Figs. 5A-C**

A Pathway enrichment results of proteome analyses (SEM cell pellet) described in **Fig. 5A** were mapped into a network of gene-sets (nodes) related by gene overlap (lines). Node size is proportional to the number of genes in each set and the enrichment significance (FDR *q*-value) is represented as a node color gradient. Proportion of shared genes between gene sets is depicted as the thickness of the blue line between nodes. The major functional groups are annotated and encircled by dotted lines.

B Extended Version of **Fig. 5B**. Enriched proteins in the proteome of SEM cells upon ADAM10 KO are listed in purple.

Supplemental Figure S13

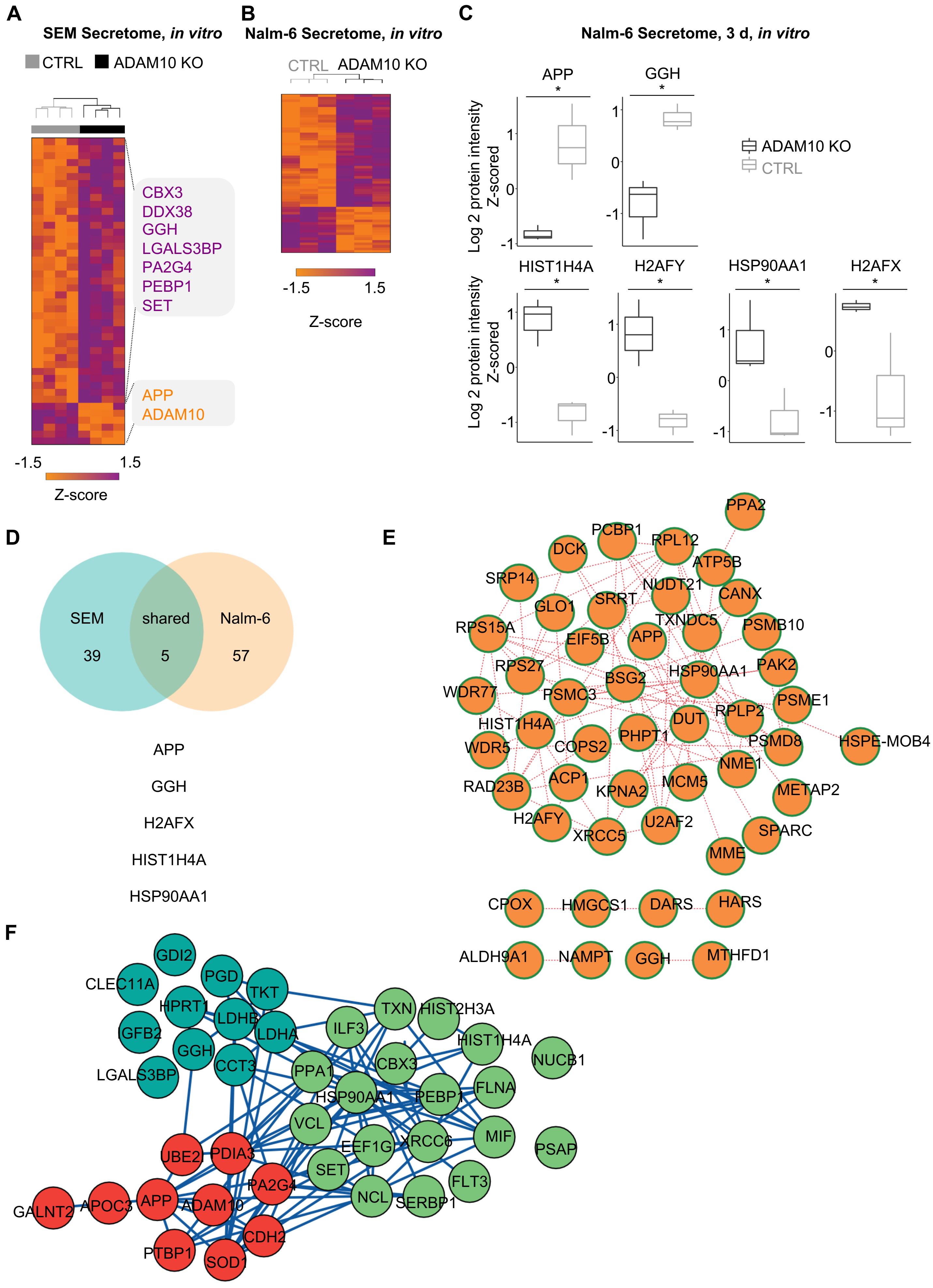

Supplemental Figure S13: Secretome analysis of ADAM10 KO cells. Data related to **Figs. 5D, E**

A Extended Version of **Fig. 5D**. Enriched proteins in the secretome of SEM cells upon ADAM10 KO are listed in purple, depleted proteins are listed in orange.

B Heat map of unsupervised hierarchical clustering of significantly regulated secreted proteome of control (CTRL) vs. ADAM10 KO in Nalm-6 cells (two-sample test, *p* value <0.05).

C Box plots showing proteins which were secreted with statistically significant difference in Nalm-6 CTRL and ADAM10 KO cells. *$p<0.05$ by paired t-test.

D Venn diagram displaying unique and shared secreted proteins in SEM (**Figs. 5D, E**) and Nalm-6 (**Figs. S14A, B**) cells upon ADAM10 KO.

E Protein-protein interaction network analysis of significantly secreted proteins in Nalm-6 cells upon ADAM10 KO. Interaction network was established using STRING database and visualized in Cytoscape.

F Protein-protein interaction network analysis of significantly secreted proteins in SEM cells with ADAM10 KO. Interaction network was established using STRING database and visualized in Cytoscape.

Supplemental Figure S14

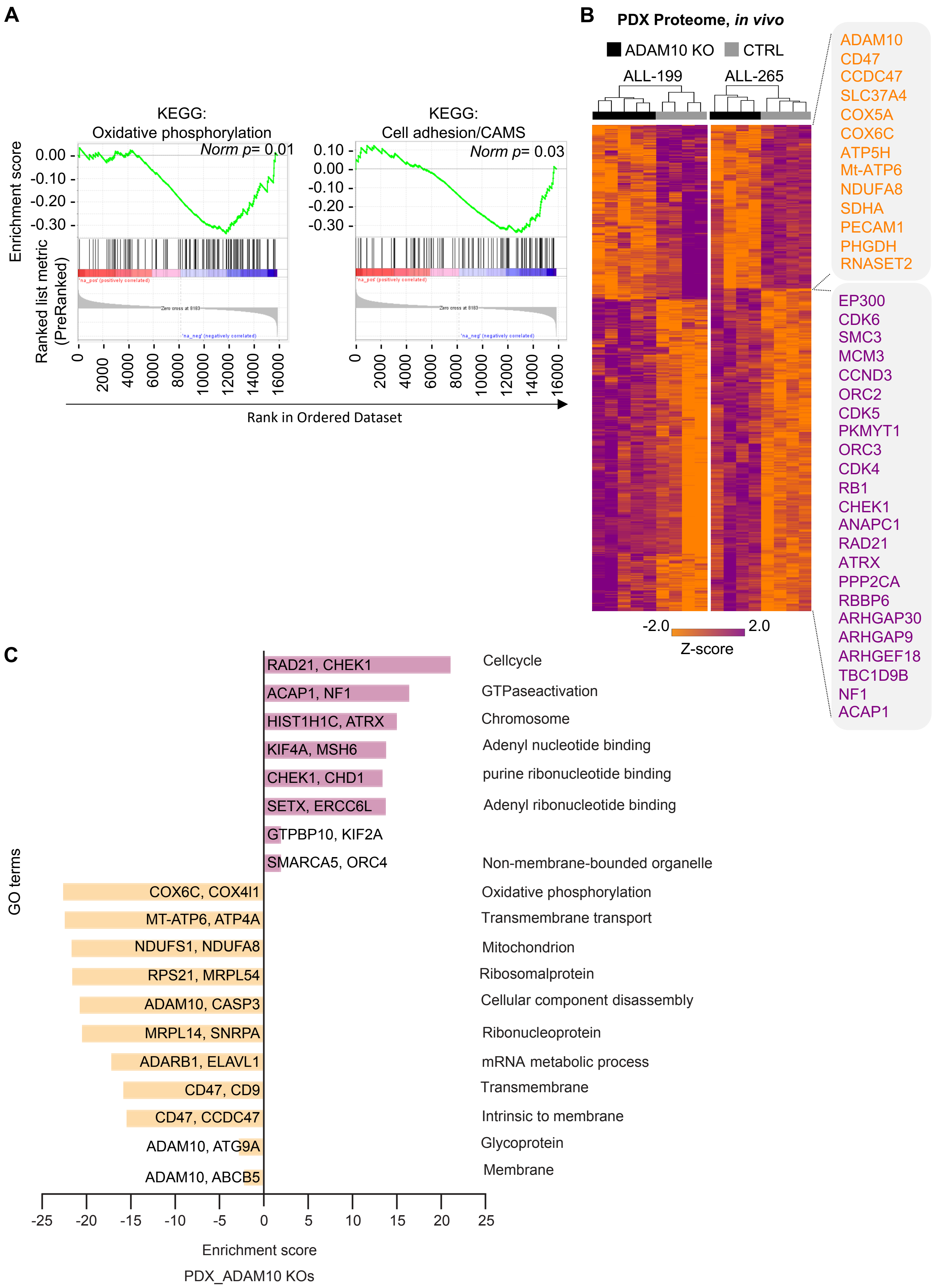

Supplemental Figure S14: ADAM10 KO transcriptome and proteome analyses in ALL PDX cells. Data related to **Figs. 5F-J**

A Gene set enrichment analysis (GSEA) of transcriptomes described in **Fig. 5F**. GSEA enrichment plot of gene sets downregulated in ADAM10 KO ALL PDX samples (p<0.005 and FDR q-value < 0.33).

B Extended Version of **Fig. 5I**. Enriched proteins in the proteome of ALL-199 and ALL-265 cells with ADAM10 KO are listed in purple, depleted proteins are listed in orange.

C Extended Version of **Fig. 5J**. For each of the enriched and de-enriched GO term categories representative proteins are indicated.

Supplemental Figure S15

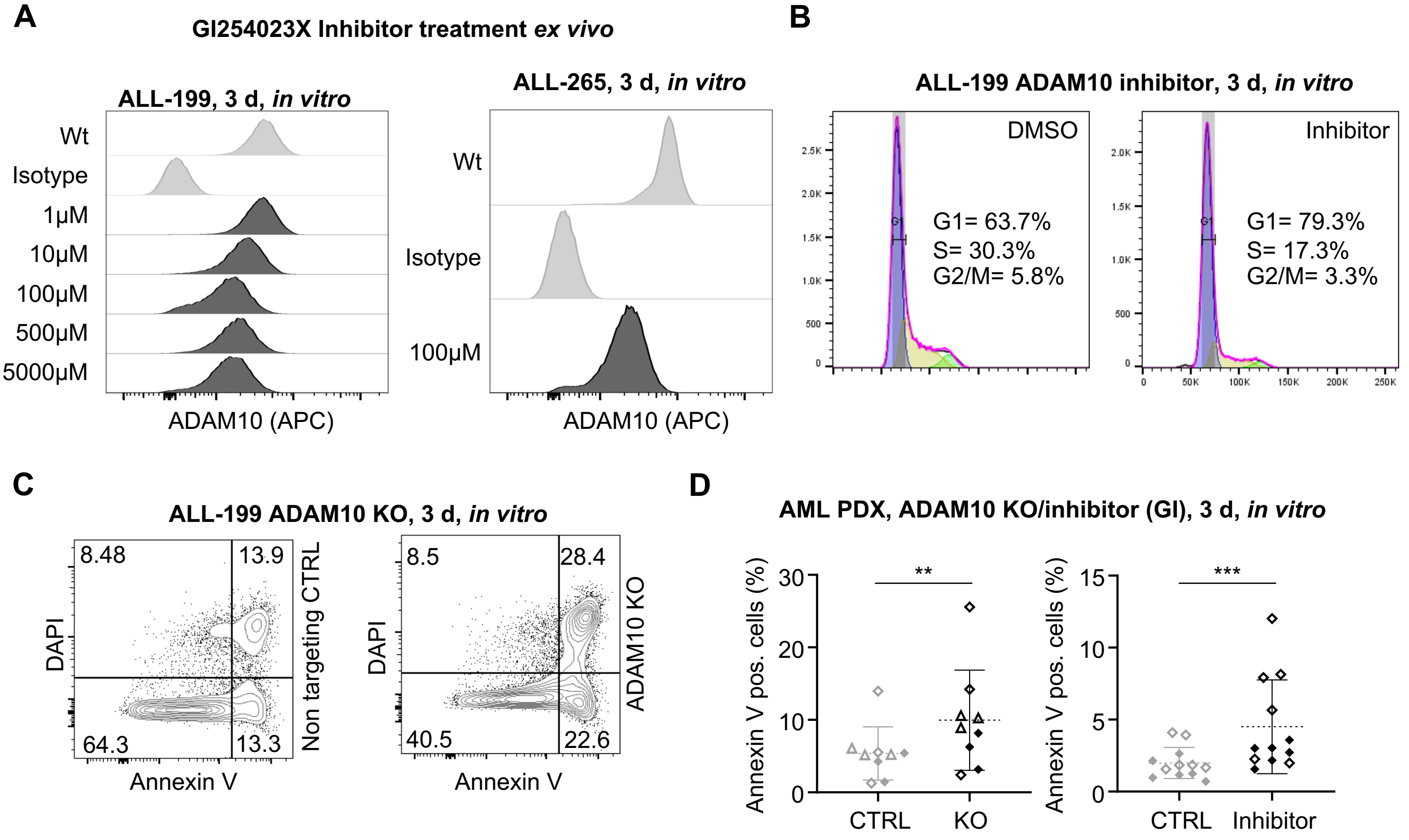

Supplemental Figure S15: Quality controls for experiments on cell cycle and apoptosis. Data related to **Figs. 5K, L**

A PDX ALL-199 and ALL-265 were treated with the indicated concentrations of the ADAM10 inhibitor GI254023X *in vitro* and ADAM10 surface protein expression was analyzed by flow cytometry. Representative histograms of a proof-of-concept experiment are shown.

B Cell cycle assay. ALL-199 PDX cells treated for three days with either an ADAM10 Inhibitor (GI254023X, 490µM) or solvent DMSO were fixed, stained with DAPI and cell cycle was analyzed by flow cytometry. One representative histogram of four independent experiments is shown. Quantification is depicted in **Fig. 5K**. G1 = Gap phase 1, S = Synthesis phase, G2/M = Gap phase 2/mitosis.

C Apoptosis of ALL-199 PDX cells three days after electroporation with sgRNAs targeting ADAM10 or non-targeting CTRL sgRNAs was analyzed following staining with Annexin V and DAPI by flow cytometry. Representative flow cytometry plots of six independent experiments are shown. Quantification is depicted in **Fig. 5L**.

D Apoptosis assay in AML PDX cells with ADAM10 KO or treated with ADAM10 inhibitor (GI254023X, 490µM). *** $p<0.001$, ** $p<0.01$ by paired t-test.

Supplemental Figure S16

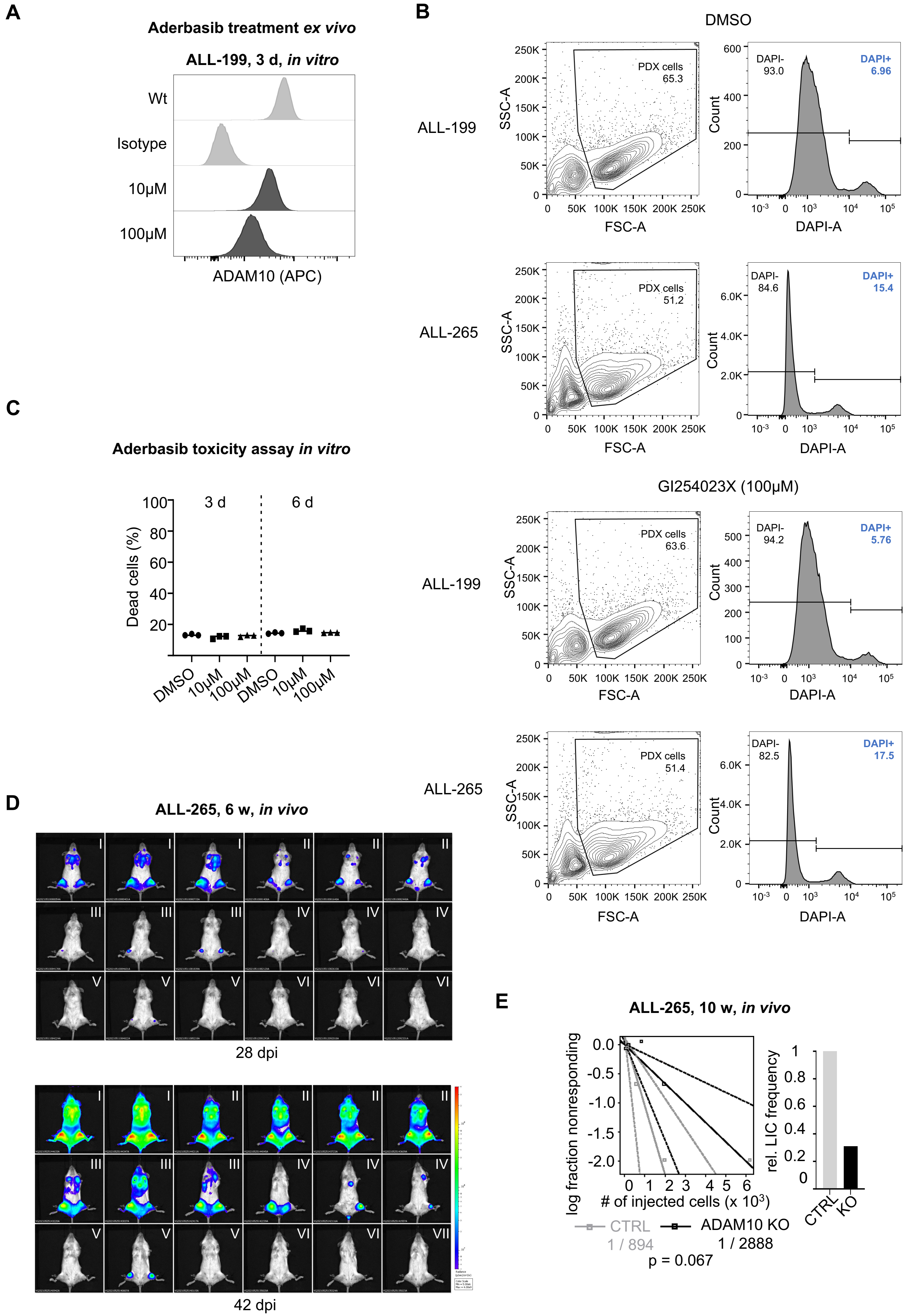

Supplemental Figure S16: Quality controls and raw data for Figs. 6B, C

A PDX ALL-199 cells were treated with the indicated concentrations of the ADAM10 inhibitor Aderbasib in vitro and ADAM10 surface protein expression was analyzed by flow cytometry. Representative histograms of a proof-of-concept experiment are shown.

B Quality control to **Fig. 6B** GI254023X at 100 µM does not impair viability of PDX cells *in vitro*. Viability of PDX ALL-199 and ALL-265 after treatment with 100 µM of ADAM10 inhibitor GI254023X or solvent DMSO was determined by live/dead cell staining. Flow cytometry plots of a quality control experiment are shown.

C Quality control to **Fig. 6B** Aderbasib at 10 µM or 100 µM does not impair viability of PDX cells *in vitro*. Viability of PDX ALL-199 after treatment with 10 µM or 100 µM of ADAM10 inhibitor Aderbasib or solvent DMSO was determined by live/dead cell staining. Quantification of flow cytometry analysis of the PDX cells used for the homing assay are shown at injection (3 d) and after 3 days *in vitro* (6 d).

D *In vivo* bioluminescence imaging of the competitive *in vivo* LDTA assay depicted in **Fig. 6C**. Number of injected cells per population is indicated by roman numerals (I = 20.000, II = 6.000, III = 2.000, IV = 600, V = 200, VI = 60, VII = 20).

E Limiting dilution transplantation assay as in **Fig. 6C** with ALL-265 (n=25). Bar graph depicts relative LIC frequency of ADAM10 KO cells normalized to control.

Supplemental Figure S17

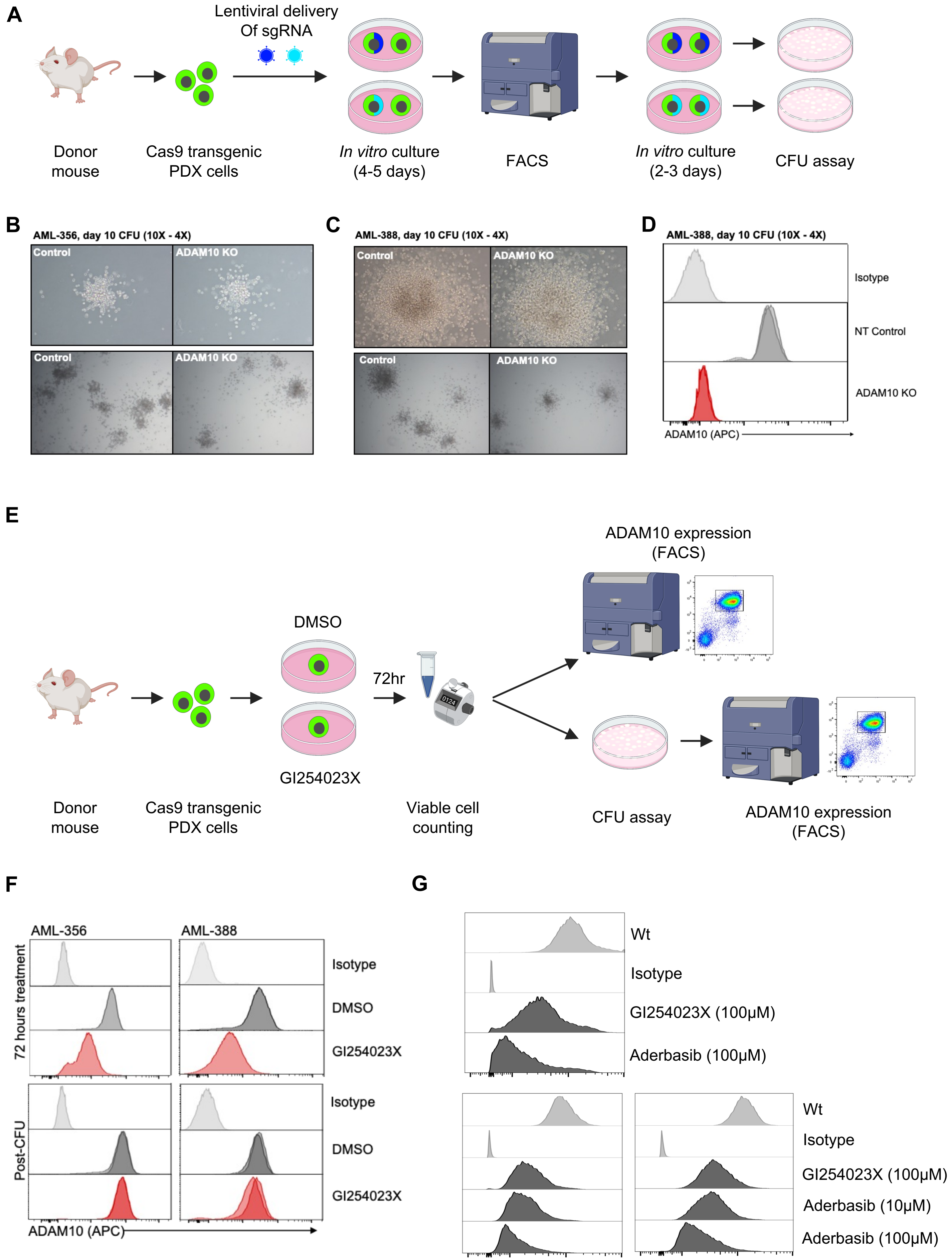

Supplemental Figure S17: ADAM10 inhibits colony formation in PDX AML cells *in vitro*. Data related to **Figs. 6D-F**

A Experimental workflow. Split-Cas9-transgenic AML-356 and AML-388 PDX cells were isolated, transduced with the ADAM10 sgRNA construct (**Fig. S3A**) and enriched. After recovery, $1x10^3$ cells were plated in methylcellulose in technical duplicates. Colonies of more than 20 cells were scored on day 10 after seeding.

BC Photomicrographs of colonies formed at day 10 by control or ADAM10 KO AML-356 (**B**) or AML-388 (**C**). Magnification 10X (upper) and 4X (lower). Representative images out of three (AML-356) and four (AML-388) independent experiments are shown, respectively.

D ADAM10 surface protein expression was analyzed at the end of the CFU assay in control (non-targeting sgRNA) and ADAM10 KO AML-388 cells by flow cytometry. One representative histogram out of four independent experiments is shown.

E Experimental workflow. Freshly isolated parental AML-388 and AML-356 PDX cells were treated *in vitro* either with DMSO or the ADAM10 inhibitor GI254023X (100 µM) for 72 h. $1x10^3$ cells were plated in methylcellulose in duplicates or triplicates and colonies of more than 20 cells were scored ten days later. ADAM10 surface expression was quantified by flow cytometry before seeding and at the end of the experiment.

F ADAM10 surface protein expression was analyzed after 72 h of treatment (upper) and at the end of the CFU assay (lower) in control (DMSO) and inhibitor treated AML-356 (left) and AML-388 (right) PDX cells. One representative histogram out of three independent experiments per PDX sample is shown.

G Quality controls to **Fig. 6F** ADAM10 surface protein expression of human CD34+ blood progenitor cells was analyzed after 72 h of treatment with ADAM10 inhibitor (GI254023X: 100 µM or Aderbasib 10 µM and 100 µM) or solvent (DMSO).

Supplemental Figure S18

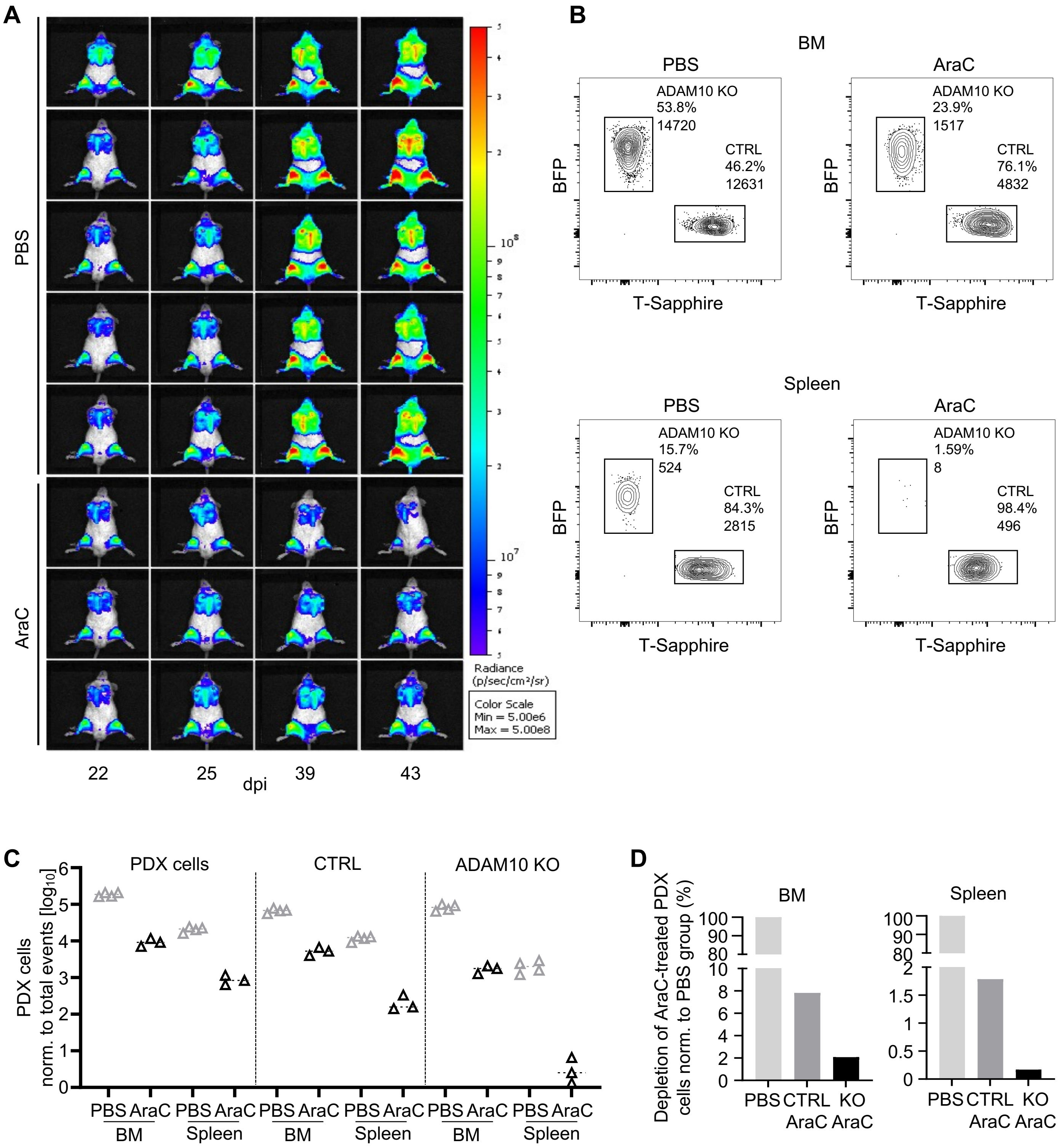

Supplemental Figure S18: ADAM10 KO increases the anti-leukemia efficacy of AraC and cyclo *in vivo*. Data related to **Figs. 6G-L**

A *In vivo* bioluminescence imaging of the competitive ADAM10 KO *in vivo* chemotherapy trial of AML-661 treated with cytarabine (AraC) or control. Representative images of experiments described in **Fig. 6G** are shown.

B Representative flow cytometry plots of the analysis of the AML-661 *in vivo* competitive chemotherapy trial shown in **Fig. 6I**. Distribution of mTagBFP-positive ADAM10 KO cells and T-Sapphire-positive CTRL cells in BM and spleen in both the PBS group and the AraC group is shown by percentages and absolute cell numbers.

C Absolute numbers of total PDX cells, and subpopulations of ADAM10 KO and CTRL cells, normalized to the total number of events and multiplied by 10^6 are depicted for the experiment described in **Figs. 6J, K**.

D Effect of AraC on cell depletion in CTRL and ADAM10 KO cells, normalized to the changes observed in the PBS-treated control group, in BM and spleen.

Supplemental Figure S19

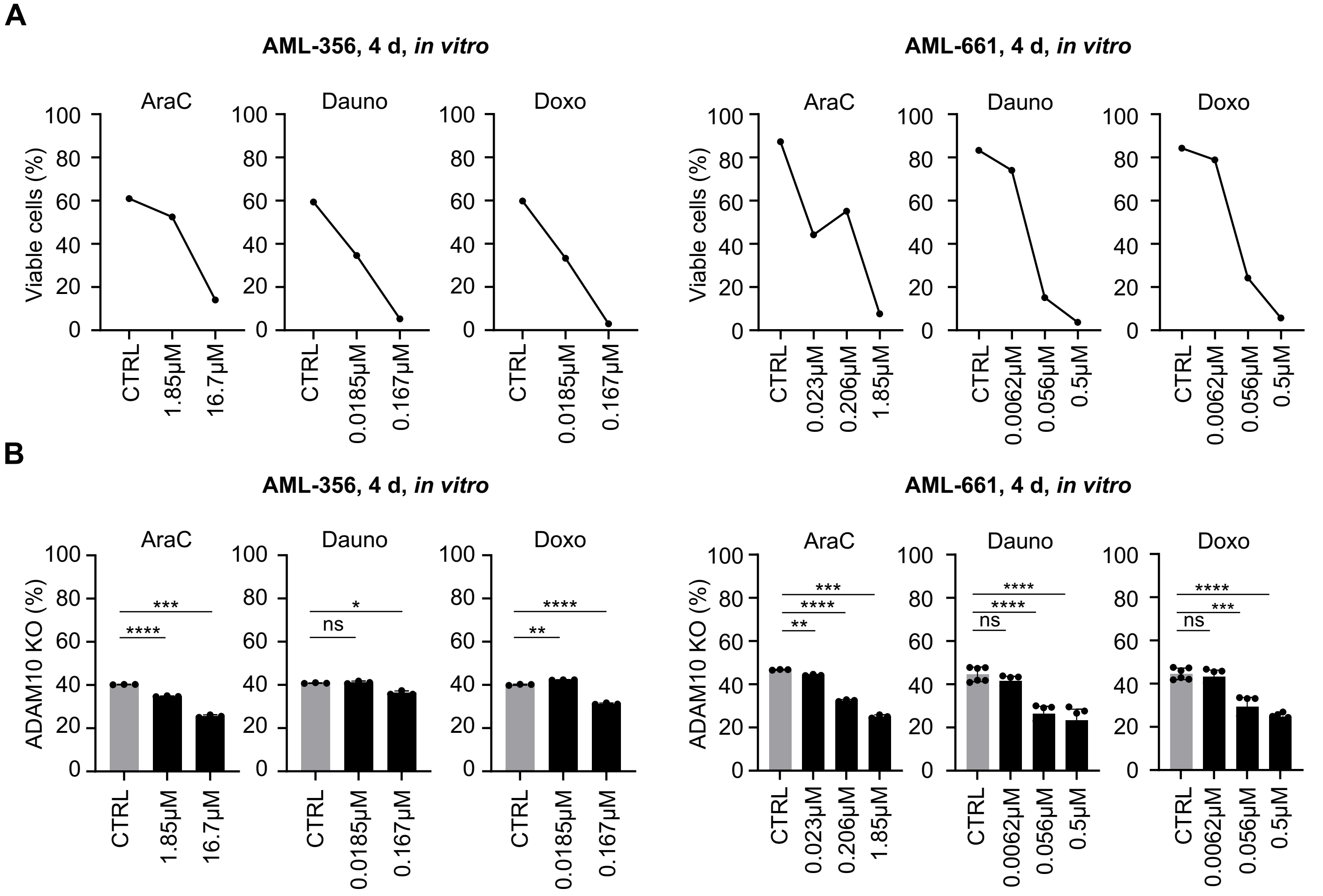

Supplemental Figure S19: ADAM10 KO increases the anti-leukemia efficacy of AraC, Daunorubicin and Doxorubicin *in vitro*.

PDX AML cells with and without ADAM10 knockout were subjected to competitive *in vitro* experiments and treated with cytotoxic drugs (n=3 per PDX sample, chemotherapeutic agent and concentration; incubation time 4 days).

A Cell viability.

B Percentage of ADAM10 KO PDX. **** p<0.0001, *** p<0.001, ** p<0.01, * p<0.05 by Brown-Forsythe and Welch ANOVA tests using multiple comparisons.
