# Supplementary material for: Combined proteomics and CRISPR‒Cas9 screens in PDX identify ADAM10 as essential for leukemia in vivo
Source: Mol Cancer. 2023 Jul 8;22:107. doi: 10.1186/s12943-023-01803-0 (PMC10329331; doi:10.1186/s12943-023-01803-0)
Supplement: Supplementary file 2 — Additional file 2. Materials and Methods. [file 12943_2023_1803_MOESM2_ESM.pdf]

Materials and Methods

1. Ethical Statements:
2. The patient-derived xenograft (PDX) model of ALL and AML:
   a. Animal Model:
   b. Generation of PDX model:
   c. Monitoring engraftment of PDX cells:
   d. *In vivo* bioluminescence imaging:
   e. Quantification of BLI pictures:
   f. Experimental end-points:
   g. *In vivo* competitive assay
   h. Early engraftment assay:
   i. *In vivo* drug treatment:
   j. Competitive LDTA:
   k. Labeling of PDX cells with far-red proliferation dye:
   l. Sorting of label-retaining cells (LRC) PDX cells in the murine BM:
   m. CRISPR screens in the PDX model:
   n. Calculation of sgRNA library size:
3. Molecular biology methods
   a. Cloning of Split-Cas9-GFP:
   b. H2Kk-mTagBFP cloning and MACS enrichment:
   c. sgRNA library design:
   d. Golden gate cloning:
   e. Next generation sequencing (NGS) of sgRNA library and data analysis:
   f. sgRNA library analysis:
   g. Lentivirus production:
   h. Lentiviral transduction:
   i. Cloning of ADAM10 variants:
   j. Genome editing analysis and Sanger sequencing:
   k. qPCR:
   l. Transcriptome analysis:
   m. Differential gene expression and protein analysis:
   n. Confocal microscopy:

4. Protein detection
   a. Simple Western (WES ProteinSimple, USA):
   b. Whole protein lysis and SDS-PAGE:
   c. Transmembrane protein purification:
5. Proteome analysis
   a. Proteome quantification of low HeLa and SJSA1 sample amounts:
   b. Label-retaining cell proteome:
   c. Data independent - PASEF acquisition runs:
   d. diaPASEF data processing:
   e. Proteome of acute leukemia cell lines, ALL PDX cells and ADAM10 KO cells:
   f. ADAM10 KO secretome:
   g. Liquid chromatography and data dependent acquisition (DDA) runs:
   h. DDA data processing:
   i. Proteome data analysis:
6. Flow cytometry
7. Cell culture and *in vitro* assays
   a. Cell culture:
   b. *In vitro* competitive assay:
   c. Cell cycle analysis:
   d. Colony-forming unit (CFU) assay:
   e. Apoptosis assay:
   f. *In vitro* chemotherapy assay:
8. Online resources and software
9. Data availability
10. References

Materials and Methods

1. Ethical Statements:

Written consent forms were obtained from all patients and from parents/carers in the cases where patients were minors. The study was performed following the ethical standards of the responsible committee on human experimentation (written approval by Ethikkommission des Klinikums der Ludwig-Maximilians-Universität München, Ethikkommission@med.unimuenchen.de, April 15/2008, number 068-08, 222-10) and with the Helsinki Declaration of 1975, as revised in 2013.
All animal trials were performed by the current ethical standards of the official committee on animal experimentation written approval by Regierung von Oberbayern, ROB-55.2Vet-2532.Vet_02-15-193, ROB-55.2Vet-2532.Vet_03-16-56, and ROB-55.2Vet-2532.Vet_02-16-7, ROB-55.2-2532.Vet_02-20-159, ROB-55.2-2532.Vet_0321-9).

2. The patient-derived xenograft (PDX) model of ALL and AML:

a. Animal Model:

Six to 16 weeks old male and female NOD.Cg-Prkdcscid IL2rgtm1Wjl/SzJ (NSG) mice (The Jackson Laboratory, Bar Harbour, ME, USA) were included. Mice were kept under specified pathogen-free (SPF) conditions with a 12/12 h light cycle, temperature of 20–24 °C and 45–65% humidity according to Annex A of the European Convention 2007/526 EC. The maximum stocking density of the cages corresponds to Annex III of the 2010/63 EU. The cages were constantly filled with structural enrichment and the animals had unlimited access to food and water. During the experiment, mice were kept in individually ventilated cages (IVCs). Hygiene monitoring was carried out at least quarterly in accordance with the current FELASA recommendation.

b. Generation of PDX model:

ALL PDX models were generated as previously described (Ebinger et al., 2016). Briefly, primary patient ALL samples were injected into the tail vein of mice to establish PDX models. PDX cells were isolated from murine BM and spleen, genetically

engineered to stably express different transgenes such as Luciferase, split-Cas9, and marker proteins, and re-transplanted into next recipient mice for additional passages.

c. Monitoring engraftment of PDX cells:

To monitor engraftment and growth of PDX cells in NSG mice, 50 µl of blood was repetitively collected by tail vein puncture every other week starting from week four after cell injection or adapted to the individual growth kinetics of each sample. Peripheral blood (PB) was analyzed by flow cytometry after staining for human CD45 and human CD33 (AML) or for murine CD45 and human CD38 (ALL). CD33 or CD38 positivity was used to confirm AML or ALL engraftment, respectively.

d. *In vivo* bioluminescence imaging:

In vivo bioluminescence imaging (BLI) was performed as previously described (Terziyska et al., 2012). Bioluminescence was measured using an IVIS Lumina 2 (Perkin Elmer) to repetitively visualize outgrowth of GEPDX cells in living mice. Mice were transferred to isoflurane inhalation anesthesia. For cells expressing a recombinant codon-optimized form of firefly luciferase, 150 mg/kg D-Luciferin (BIOMOL GmbH, Hamburg, Germany) was injected into the tail vein. Pictures were taken immediately for 30 sec or up to two minutes using a field of view of 12.5 cm with binning 8, f/stop 1 and open filter setting. Pictures with more than 1000 counts were considered within the linear range; saturated pictures were repeated the next day with reduced exposure time or reduced f-stop.

e. Quantification of BLI pictures:

Quantification of BLI signal was performed as previously described (Terziyska *et al.*, 2012). Quantification of light emission was performed using the Living Image software (Caliper Life Sciences, Mainz, Germany). A region of interest (ROI) covering the whole mouse was used and total flux was determined. Values were always given on a logarithmic scale.

f. Experimental endpoints:

Most donor mice used for PDX cell amplification were sacrificed at advanced leukemic disease as measured by blood sampling or *in vivo* imaging, or when mice showed first clinical signs of illness (rough fur, hunchback, reduced motility, paralysis). If 20 to 25 weeks after cell injection no clinical signs of leukemia appeared, mice were sacrificed and BM was analyzed by flow cytometry for human cell engraftment. In therapy trials, mice were sacrificed upon body weight loss beyond 15% as sign for therapy-related toxicity. Mice showing leukemia-unrelated illness or peculiarities were excluded from the studies as stated in each legend. Mice were sacrificed by exposure to CO_2 or cervical dislocation.

g. *In vivo* competitive assay:

Cas9-PDX cells were transduced with either a sgRNA targeting the gene of interest coupled to H-2Kk-BFP expression or a non-targeting control sgRNA coupled to H-2Kk-T-Sapphire expression. After 7-10 days *in vitro*, allowing KO establishment, KO and control cells were mixed 1:1 by flow cytometry and injected into mice. At the experimental endpoint, mice were sacrificed and the PDX cells were isolated from BM and spleen. The distribution of KO vs. control cells was measured using flow cytometry.

h. Early engraftment assay:

Cas9-PDX cells were treated *ex vivo* with GI254023X (100 µM; Absource, Germany), Aderbasib (100 µM; MedChemExpress, USA) or solvent (DMSO, Thermo Fisher Scientific, USA) for two days. 10^7 treated PDX cells were injected into mice (GI254023X: ALL-199 n=6 DMSO, n=8 GI254023X; ALL-265 n=5 both DMSO and Inhibitor; Aderbasib: ALL-199 n=5 for both groups) and the animals were sacrificed three days post injection. The number of human cells in the BM was analyzed by flow cytometry.

i. *In vivo* drug treatment:

For *in vivo* treatment with Cytarabine (Cell Pharma GmbH, Bad Vilbel, Germany), mice were injected with luciferase expressing AML-661 ($2.5x10^6$ cells/mouse). Tumor growth was monitored once per week by bioluminescence imaging and after five weeks mice were treated i.p. with Cytarabine 100 mg/kg (n=3) or vehicle (n=4) four times per week.

For *in vivo* drug treatment with Cyclophosphamide (Cyclo, Baxter Deutschland GmbH, Unterschleißheim, Germany) and Vincristine (VCR, Stada, Bad Vilbel, Germany), mice were injected with ALL-265 ($7.5x10^5$ cells/mouse). Animals were divided into two groups and treated with Cyclo (70mg/kg i.p. once per week (n= 3)), VCR ((0.3 mg/kg i.v. once per week (n=3)) or vehicle (n=4).

To determine the drug response of mice in all experimental groups, cells from BM and spleen of each group were re-isolated and analyzed by flow cytometry.

j. Competitive LDTA:

Freshly isolated PDX donor cells were thoroughly counted and serially diluted to the cell numbers of interest. For competitive LDTAs, donor cells were mixed 1:1 as quality controlled by flow cytometry. Different numbers of cells were injected into groups of NSG mice. Engraftment and tumor growth were monitored via *in vivo* bioluminescence imaging. As soon as engraftment was observed in imaging, the mice were sacrificed, PDX cells were isolated from the BM and the distribution of the cell populations analyzed via flow cytometry followed by analysis on FlowJo Software (FlowJo™ Software, version 10.7, Ashland, USA). For positive engraftment, a threshold of 1% of the population of interest in the BM was defined. LIC frequency was calculated using the webtool of the ELDA software (http://bioinf.wehi.edu.au/software/elda/index.html).

k. Labeling of PDX cells with far-red proliferation dye:

PDX cells were labeled with far-red proliferation dye (CellTraceTM Far Red – Cell proliferation kit, Thermo Fisher Scientific, USA) similar as described previously for CFSE (Ebinger *et al.*, 2016). The far-red proliferation dye was used as staining with CFSE is not compatible with GFP-expressing cells. In brief, ALL PDX cells were isolated from mice with advanced leukemia. PDX cells were labeled with the far-red proliferation dye according to the manufacturer's protocol, washed with PBS and injected into recipient mice (10^7 PDX cells per animal).

l. Sorting of label-retaining cells (LRC) PDX cells in the murine BM:

Experiments were performed as described previously (Ebinger *et al.*, 2016). In brief, ALL PDX cells were isolated from the BM, washed with PBS, and filtered using a cell strainer (EASYSTRAINER 70 µM, Greiner bio-one, Frickenhausen, Germany). Murine cells were depleted using magnetic beads in an adapted version of the manufacturer's

protocol (Mouse Cell Depletion Kit, Miltenyi Biotech, Bergisch Gladbach, Germany) with 200 µl MicroBeads and two columns per animal. To enrich LRC and non-LRC PDX cells by flow cytometry, PDX cells were identified via FSC/SSC, and GFP (Cas9) and mCherry (Luciferase) double-positivity using the FACSAriaIII (BD Biosciences, Heidelberg, Germany). LRC and non-LRC PDX cells were separated using the purity setting on the FACSAriaIII device. Slow-cycling ALL PDX cells (LRC) were separated from fast-cycling non-LRC cells based on the far-red proliferation dye intensity. For LRC quantification, the mean fluorescence intensity (MFI) of labeled PDX cells incubated for 48 h *ex vivo* was measured and defined as starting point ("zero divisions''). The starting point MFI was divided using factor two to mimic cell divisions. PDX cells with less than three cell divisions were defined as LRC, while more than seven MFI divisions were defined as complete loss of the far-red proliferation dye, indicating fast-cycling non-LRC.

m. CRISPR screens in the PDX model:

For *in vivo* CRISPR screen experiments, fresh Cas9-expressing PDX cells were transduced with lentiviral CRISPR library at MOI~ 0.2 and cultured for about 3-4 days *in vitro*. H-2Kk-mTagBFP-expressing cells were enriched by MACS and kept in culture for another 7-10 days. Cells were counted and the viable cells injected into the tail vein of experimental mice. Leukemia growth was monitored using blood counting as described above. Mice with high tumor loads were sacrificed and PDX cells were re-isolated from BM (femurs, tibiae, hips, sternum, spine) and spleen as described previously (Ebinger *et al.*, 2016).

n. Calculation of sgRNA library size:

The engraftment efficiency of the two PDX samples, which were used in this study, was determined as previously reported (Ebinger *et al.*, 2016). Engraftment and homing efficiency were used for the calculations. To determine a sgRNA library size which would allow retrieving the entire sgRNA library from a single mouse, the following formula was used:

$$\frac{\#\ of\ injected\ cells\ \times\ engraftment\ efficiency}{sgRNA\ fold\ coverage\ \times\ \#\ of sgRNAs\ per\ gene}$$

- Maximum cell number permitted to inject into a single mouse = 10^7
- Engrafting and homing efficiency = 1%

– sgRNA fold coverage = 200

– Number of sgRNAs targeting a gene = 5

= 100 genes

The number of donor mice required for generating sufficient Cas9-positive PDX cells for each biological replicate was determined based on the number of technical replicates, transduction efficiency favoring single integration per cell, cell viability after 10-14 days of *in vitro* culture and cells retrieved from each mouse. The following formula was used to determine the number of donor mice required for every independent biological experiment:

$$\frac{Technical\ replicate \times \#\ of\ injected\ cells\ per\ mouse}{Transduction\ efficiency \times In\ vitro\ cell\ viability} \times \frac{1}{Average\ re\text{-}isolated\ cells\ per\ mouse}$$

– Technical replicates = 4

– # of injected cells per mouse = 10^7

– Transduction efficiency = 0.2

– *In vitro* cell viability = 0.5

– Average re-isolated cells per mouse = 1.5 $x10^8$

~ 3 donor mice

3. Molecular biology methods

a. Cloning of split-Cas9-GFP:

The split-Cas9 design was adapted from (Truong et al., 2015) and combined with a split-GFP BiFC module (Kerppola, 2008; Volkmann et al., 2016; Yang and Reth, 2010). In brief, the N-terminal and C-terminal fragments of Cas9 were fused to N-Intein and C-Intein DNA fragments, respectively. These constructs were further complemented with GFP fragments fused to leucine-zippers for reassembly of GFP after protein expression. Cloning was performed using the Takara InFusion HD cloning Kit according to the manufacturer’s instructions. Individual DNA fragments were prepared by PCR amplification from donor plasmids (i.e., for Cas9 fragments) or gBlocks (inteins and GFP-leucine-zipper fragments) (Integrated DNA Technologies (IDT), Belgium).

InFusion assembly was subsequently performed using a pCDH lentiviral vector carrying an SFFV promoter.

b. H-2Kk-mTagBFP cloning and MACS enrichment:

H-2Kk-mTagBFP fusion cassette was ordered as gBlock from IDT and cloned into pCDH lentiviral construct over EcoRI, SalI restriction sites. H-2Kk-mTagBFP protein expression was confirmed by transient and stable plasmid expression in HEK293T and Nalm-6 cell lines. (H-2Kk-mTagBFP fusion cassette sequence:

ATGGCACCCTGCATGCTGCTCCTGCTGTTGGCGGCCGCCCTGGCCCCGACTCA
GACCCGCGCGGGCCCACATTCGCTGAGGTATTTCCACACCGCCGTGTCCCGGC
CCGGCCTCGGGAAGCCCCGGTTCATCTCTGTCGGCTACGTGGACGACACGCA
GTTCGTGCGCTTCGACAGCGACGCGGAGAATCCGAGGTATGAGCCGCGGGTG
CGGTGGATGGAGCAGGTGGAGCCCGAGTATTGGGAaCGGAACACGCAGATCG
CCAAGGGCAATGAGCAGATTTTCCGAGTGAACCTGAGGACCGCGCTGCGCTAC
TACAACCAGAGCGCGGGCGGCTCTCACACGTTCCAACGGATGTACGGCTGTGA
GGTGGGGTCGGACTGGCGCCTCCTCCGCGGGTACGAGCAGTACGCATACGAC
GGCTGCGATTACATCGCCCTGAACGAGGATCTGAAAACGTGGACGGCGGCCGA
CATGGCGGCGCTGATCACCAAACACAAGTGGGAGCAGGCTGGTGATGCAGAGA
GAGACCGGGCCTACCTGGAGGGCACGTGCGTGGAGTGGCTCCGCAGATACCT
GCAGCTCGGGAACGCGACGCTGCCGCGCACAGATTCCCCAAAGGCCCATGTG
ACCCGTCACAGCAGACCTGAAGATAAAGTCACCCTGAGGTGCTGGGCCCTGGG
CTTCTACCCTGCTGACATCACCCTGACCTGGCAGTTGAATGGGGAGGAGCTGA
CCCAGGACATGGAGCTTGTGGAGACCAGGCCTGCAGGGGATGGAACCTTCCA
GAAGTGGGCATCTGTGGTGGTGCCTCTTGGGAAGGAGCAGTATTACACATGCC
ATGTGTACCATCAGGGGCTGCCTGAGCCCCTCACCCTGAGATGGGAGCCTCCT
CCATCCACTGTCTCCAACACGGTAATCATTGCTGTTCTGGTTGTCCTTGGAGCT
GCAATAGTCACTGGAGCTGTGGTGGCTTTTGTGATGAAGATGAGAAGGAGAAAC
ACAGGTGGAAAAGGAGGGcgcggatccATGAGCGAGCTGATTAAGGAGAACATGC
ACATGAAGCTGTACATGGAGGGCACCGTGGACAACCATCACTTCAAGTGCACAT
CCGAGGGCGAAGGCAAGCCCTACGAGGGCACCCAGACCATGAGAATCAAGGT
GGTCGAGGGCGGCCCTCTCCCCTTCGCCTTCGACATCCTGGCTACTAGCTTCC
TCTACGGCAGCAAGACCTTCATCAACCACACCCAGGGCATCCCCGACTTCTTCA
AGCAGTCCTTCCCTGAGGGCTTCACATGGGAGAGAGTCACCACATACGAGGAT
GGGGGCGTGCTGACCGCTACCCAGGACACCAGCCTCCAGGACGGCTGCCTCA

TCTACAACGTCAAGATCAGAGGGGTGAACTTCACATCCAACGGCCCTGTGATGC AGAAGAAAACACTCGGCTGGGAGGCCTTCACCGAGACGCTGTACCCCGCTGAC GGCGGCCTGGAAGGCAGAAACGACATGGCCCTGAAGCTCGTGGGCGGGAGCC ATCTGATCGCAAACATCAAGACCACATATAGATCCAAGAAACCCGCTAAGAACC TCAAGATGCCTGGCGTCTACTATGTGGACTACAGACTGGAAAGAATCAAGGAGG CCAACAACGAGACCTACGTCGAGCAGCACGAGGTGGCAGTGGCCAGATACTGC GACCTCCCTAGCAAACTGGGGCACAAGCTTAATTAA)

For MACS enrichment, H-2Kk-mTagBFP-expressing cells were harvested and washed in enrichment buffer (PBS buffer containing 0.5% BSA). 10^7 cells were resuspended in 1 ml enrichment buffer containing 40 µl anti-H-2Kk microbeads (130-070-201, Miltenyi Biotec, Germany) and incubated at RT protected from light for 20 min on a tube rotator. Three ml enrichment buffer were added to the cells and samples loaded into a primed LS magnetic column attached to the magnets (130-042-401, Miltenyi Biotec, Germany). Columns were washed twice with 2 ml enrichment buffer and the flow-through collected as a negative fraction for quality control. The positive fraction was eluted in a 5 ml enrichment buffer. Purity and enrichment efficiency was assessed by flow cytometry, checking mTagBFP and H-2Kk expression, stained with anti-H-2Kk-APC (130-102-346, Miltenyi Biotec, Germany), in positive and negative fractions.

c. sgRNA library design:

Ninety-two cell surface molecules were selected from our multi-omics data, complemented with candidates from literature. Five sgRNAs per gene were designed using the Broad Institute sgRNA designer (https://portals.broadinstitute.org/gpp/public/) and extra nucleotide overhangs were incorporated into the forward and reverse oligos. A set of 20 non-targeting sgRNAs was added to the list. Oligos were ordered from Sigma-Aldrich company in 96-well plates with the following structure.

Forward oligo: 5’-TCCCGN_{20}(Target)-3’
Reverse oligo: 5’ AAACN_{20}(Target)-3’

d. Golden gate cloning:

2 µl of forward (100 µM) and 2 µl of reverse (100 µM) oligos were mixed with 2 µl T4 DNA ligase buffer (El0012, Thermo Fischer Scientific, USA) and 14 µl H_2O and annealed in a thermocycler set at 95°C for 5 min ramping down to 25°C at 0.1°C/sec.

2 µl of each annealed sgRNA were pooled and a dilution of 1/500 used for the golden gate reaction as following:

- 2 µl of the diluted pool
- 2 µl of T4 ligase buffer (El0012, Thermo Scientific, USA)
- 1 µl FastDigest BpiI (FD1014, Thermo Scientific, USA)
- 1 µl T4 Ligase (El0012, Thermo Scientific, USA)
- 100 ng pre-digested pCDH-H-2Kk-mTagBFP lentiviral plasmid (500 ng plasmid digested with 0.5 µl FD BpiI enzyme at 37°C for 10 min)
- H_2O up to 20 µl

The reaction was incubated in a thermocycler with the following setting: 20 cycles of 3 min at 37 °C followed by 10 min at 16°C and a final step of 5 min at 55°C and 5 min 80°C for enzyme inactivation. 2.5 µl of the reaction was used for electroporation of Endura competent bacteria according to the manufacturer's protocol (60240-1, Lucigen, USA). 500 µl of the transformed bacteria were plated on a 24 cm^2 culture plate. Further dilutions of 1/1000 and 1/10000 (from original transformation) were plated in a 10 cm petri dish to calculate the final colony numbers representing transformation efficiency. All plates were incubated overnight at 32°C. Representation of minimum of 50 colonies per sgRNA was considered as the sufficient colony number for further steps. Colonies were harvested from 24 cm^2 plates and plasmid DNA was isolated using the NucleoBond® Xtra Midi kit (740410-100, Macherey-Nagel, Germany).

e. Next generation sequencing (NGS) of sgRNA library and data analysis:

A nested PCR approach was used to amplify sgRNA libraries for NGS (Figure S4 and Tables S4 and S5). In the first PCR reaction, 1.7 µg gDNA (for minimum 500-fold sgRNA coverage) or 50 ng plasmid DNA per reaction were used mixed with 0.7 µl ExTaq DNA Polymerase (RR001C, Takara, Japan), 5 µl of 10× Ex Taq buffer, 4 µl of dNTP provided with the enzyme, 1.5 µl cPPT-Forward primer (5 µM) and 1.5 µl EF1α-reverse (5 µM), and up to 50 µl H_2O. Samples were amplified using the following settings: an initial 5 min at 95 °C; followed by 30 sec at 94 °C, 30 sec at 57 °C, 30 sec at 72 °C, for 16 cycles; and a final 5 min extension at 72 °C. 5 µl PCR product of the first reaction were mixed with 1.5 µl ExTaq Polymerase, 10 µl of ExTaq 10x buffer, 8 µl dNTPs, 0.5 µl P5-H1 primers mixture containing staggers (100 µM), 10 µl P7- EF1α primer (5 µM) containing unique index barcodes, and up to 100 µl H_2O and amplified

using the following PCR protocol: 3 min at 95°C; followed by 25 cycles of 30 sec at 94°C, 30 sec at 53°C, 20 sec at 72°C, and a final 10 min extension at 72°C.

10 µl of PCR reactions were visualized on a 2% agarose gel and the rest of samples cleaned up using Qiagen PCR purification kit (28006, Qiagen, Netherlands).
Samples were sequenced on a HiSeq2000 (Illumina). Sequencing reads were demultiplexed using Illumina Demultiplex software (Galaxy Version 1.0.0) and the 20 nucleotides sequences of sgRNAs were mapped to the sgRNAs reference list (Table S4). sgRNA normalized read count tables were generated using the MAGeCK-count algorithm (Li et al., 2014) which was further used to perform dropout screening with the MAGeCK-test pipeline (Li *et al.*, 2014).

f. sgRNA library analysis:

MAGeCK-count and MAGeCK-test algorithms on Galaxy server (Galaxy Versions 0.5.7.1 and 0.5.7.1, respectively) were used for generating sgRNA read counts, *p* value of depleted candidates, and the depletion scores (RRA) (Li *et al.*, 2014). Pearson correlation analysis was used to evaluate correlation between mice replicates.

g. Lentivirus production:

Lentiviral particles were produced using third-generation packaging plasmids pMDLg/pRRE, pRSV-Rev and pMD2-G as described previously (Ebinger *et al.*, 2016). Virus titration was determined by transduction of Nalm-6 cell lines, followed by flow cytometry analysis of the transgene marker. CRISPR library viruses were produced in 4x75 cm^2 cells culture flasks and the pooled supernatant was concentrated using cellulose membrane concentrating columns according to the manufacturer's protocol (UFC910008, Millipore, Germany). All the viruses were stored at -80°C until further use.

h. Lentiviral transduction:

At advanced leukemia, donor mice were sacrificed. PDX cells were isolated from murine BM and resuspended in their respective medium as stated below. $1x10^7$ cells per ml medium were transferred into wells of a 6-well cell culture dish and transduced overnight using the lentiviral constructs in the presence of 8 µg/ml polybrene (Sigma-Aldrich). In order to favor single integrations, transductions were conducted at MOIs below 0.3. After a 24 h incubation period, cells were washed three times with PBS and

resuspended in medium. Not earlier than three days after transduction, in case of sgRNAs 7-10 days, marker-positive cells were enriched by flow cytometry and injected into recipient mice.

i. Cloning of ADAM10 variants:

ADAM10 variants were designed *in silico* and ordered as gBlocks from IDT (Integrated DNA Technologies, USA) and cloned into pCDH lentiviral constructs using the AgeI and XhoI restriction sites. The ADAM10 variants were cloned into a bi-cistronic expression cassette with the variant followed by a T2A peptide and a T-Sapphire fluorochrome, which could be used for both flow cytometry measurements and FACS enrichment.

j. Genome editing analysis and Sanger sequencing:

gDNA was extracted from $1x10^6$ PDX or Nalm-6 cells, using Qiagen DNA Mini kit (51306, Qiagen, Netherlands). 100 ng gDNA was used for amplification of sgRNA targeting locus using specific primers (Supplemental Table S4). PCR amplicons were visualized and extracted (740986.20, Macherey-Nagel, Germany) from the agarose gel and sent for Sanger sequencing. Cas9-induced gene editing efficiency was evaluated using the Inference of CRISPR edits (ICE) algorithm (Brinkman et al., 2014).

k. qPCR:

Cells were enriched by flow cytometry, washed, and RNA isolated using the RNeasy Micro kit (#74004, RNeasy Micro Kit, Qiagen, Hilden, Germany) according to the manufacturer's protocol. RNA was quantified using a spectrophotometer and transcribed into cDNA using the QuantiTect Reverse Transcription Kit (#205313, QuantiTect Reverse Transcription Kit, Qiagen, Hilden, Germany) taking 400 ng of RNA as input for the reaction. Relative mRNA expression was measured using specific ADAM10 primers and including HPRT as well as GAPDH as housekeeping genes with the LightCycler® 480 SYBR Green I Master mix (#04707516001, LightCycler® 480 SYBR Green I Master, Roche, Rotkreuz, Switzerland). Samples were run on a LightCycler-® 480 Instrument II (LightCycler-® 480 Instrument II, Roche, Rotkreuz, Switzerland) using the slightly adapted SYBR Green I Master program. Data pre-analysis was performed on the device and relative expression quantified via the $\Delta\Delta CT$ method.

l. Transcriptome analysis:

Whole-Transcriptome analysis of PDX-ALL samples following TruSeq Stranded mRNA library preparation (Illumina) and paired-end 100 bp sequencing on an HiSeq 4000 instrument (Illumina) was performed (median read count 61 million/sample; range 53 - 69 million/samples). Between 700-1000 ng of total RNA from mouse cell-depleted bulk ALL-PDX samples with a minimum RNA integrity number (RIN) of 7 were used as input material. Salmon (v0.9.1) was used for gene and transcript expression analysis (corresponding to the GRCh37 release 87 reference genome) (Patro et al., 2017). R version 3.6.1 was used for statistical analysis (R Core Team (2014). R: A language and environment for statistical computing. R Foundation for Statistical Computing, Vienna, Austria. URL http://www.R-project.org/.). The gene expression data were made publicly available through the Gene Expression Omnibus Website (GSE139553).

m. Differential gene expression and protein analysis:

The raw gene expression read counts were pre-processed with the R package *edgeR* (version 3.30.3), and the differential gene and protein expression was done with the R package *limma* (version 3.44.3). For gene expression data, genes with a *p* value of ≤0.05 and |logFC|>1 were defined as differentially expressed with statistical significance. For protein data, proteins with an adjusted *p* value of ≤0.05 were defined as significant.

n. Confocal microscopy:

HEK293T cells were fixed with 4% PFA (#CAS 30525-89-4, Paraformaldehyde solution 4% in PBS, Santa Cruz Biotechnology Inc., Dallas, USA) for 15 min and washed with PBS (#11503387, Fisher Scientific GmbH, Schwerte, Germany) three times. Primary antibodies (#14194, ADAM10 Antibody; #13202, V5-Tag (D3H8Q) Rabbit mAb, Cell Signaling Technology, Boston; #857.800.000, Anti-Human ADAM-10 (11G2), Diaclone SAS, Besancon Cedex, France) were incubated for 1 h at RT either in PBS + 0.1% BSA (#A3059-50G, Bovine Serum Albumin, heat shock fraction, protease-free, essentially globulin free, pH 7, ≥98%, Sigma-Aldrich Chemie GmbH, Taufkirchen, Germany) for extracellular staining or in PBS + 0.1% BSA + 0.1% saponin (#34655.01, Saponin, SERVA Electrophoresis GmbH, Heidelberg, Germany) for intracellular staining of the proteins of interest. After three washing steps with PBS,

cells were incubated with the secondary antibodies (#A21235, IgG (H+L) Cross-Adsorbed Goat anti-Mouse, Alexa Fluor™ 647, Invitrogen™; # A21244, IgG (H+L) Cross-Adsorbed Goat anti-Rabbit, Alexa Fluor™ 647, Invitrogen™, Fisher Scientific GmbH, Schwerte, Germany) for 1 h at RT in the same buffer. For DAPI (#D9542-1MG, 4′,6-Diamidino-2-phenyl-indol-dihydrochlorid, Sigma-Aldrich Chemie GmbH, Taufkirchen, Germany) staining, incubation was for 3 min at RT in the same buffer. Samples were washed three times using PBS, mounted (#P10144, ProLong™ Gold Antifade Mountant, Fisher Scientific GmbH, Schwerte, Germany), and sealed using nail polish. Acquisition of confocal z-stacks was performed using a Leica TCS SP5 confocal microscope (Leica Mikrosysteme Vertrieb GmbH, Wetzlar, Germany) with the 405 nm UV diode and 633 nm helium/neon laser line using the 63x, 1.4 NA oil objective. Image analysis was performed using FIJI (Schindelin et al., 2012).

4. Protein detection

a. Simple Western (WES ProteinSimple, USA):

Nalm-6 cells were incubated in lysis buffer (#9803, Cell Signaling Technology, Boston, Boston, USA) supplemented with 1:200 Phenylmethylsulfonyl fluoride (PMSF, 8553, Cell Signaling Technologies, USA) on ice for 30 min. Protein concentration was determined by BCA assay (Bicinchoninic Acid Assay) and Simple Western size-based protein quantification and analysis was performed following the manufacturer's instructions and as described previously (Liu et al., 2020). Primary antibodies targeting Flag-Tag (MAB8529, R&D Systems) and β-Actin (NB600-501SS, Novus biologicals) were used and the results were analyzed using Compass software v.4.0 (*https://www.proteinsimple.com/compass/*).

b. Whole protein lysis and SDS-PAGE:

PDX cells or cell line samples were lysed by adding lysis buffer (#9803, Cell Signaling Technology, Boston, USA) together with PMSF (1:200) and incubating the samples 30 min on ice. After centrifugation at full speed (>20.000xg) for 20-30 min at 4°C, the supernatant was transferred to fresh pre-chilled tubes. Relative protein concentrations were measured via Bradford Assay (#5000006, Protein Assay Dye Reagent Concentrate, Bio-Rad Laboratories GmbH, Feldkirchen, Germany). Pre-cast SDS-PAGE gels (#4561096, 4–20% Mini-PROTEAN® TGX™ Precast Protein Gels, 15-

well, 15 µl, Feldkirchen, Germany) were used to separate the proteins. Proteins were blotted to PVDF membrane via a semi-dry blotting system (#1704272, Trans-Blot Turbo RTA Mini 0.2 µm PVDF Transfer Kit, Feldkirchen, Germany). The membrane was blocked via incubation with 5% skim-milk (#42590.01, Skim Milk Powder for blotting, SERVA Electrophoresis GmbH, Heidelberg, Germany) TBS/T (0.025% Tween) for at least 1 h at 4°C. Incubation with the primary antibody (#14194, ADAM10 Antibody; #67657, Syntaxin-4 (E6W7B) Rabbit mAb, Cell Signaling Technology, Boston) was done overnight at 4°C. Secondary antibodies (#7076, Anti-mouse IgG, HRP-linked Antibody or #7074, Anti-rabbit IgG, HRP-linked Antibody, Cell Signaling Technology, Boston, USA) were incubated for 1 h at RT. A GAPDH antibody directly coupled to HRP (GAPDH Antibody (H-12): sc-166574, Santa Cruz Biotechnology Inc., Dallas, USA) was incubated for 30 min at RT. All antibodies were incubated in 5% skim-milk TBS/T. Proteins of interest were detected using the ECL system (#34095, SuperSignal™ West Femto Maximum Sensitivity Substrate, Fisher Scientific GmbH, Schwerte, Germany).

c. Transmembrane protein purification:

Cells were harvested following trypsinization, washed twice in cold PBS and separated in two equal fractions for subsequent transmembrane protein (TMP) purification and total cell lysate preparation. For TMP purification, cells were resuspended in buffer A (50 mM Tris, pH8, 0.5 mM DTT, 0.1%(v/v) NP-40, Protease inhibitor (1mM PMSF, 5ug/ml Leupeptin), Phosphate inhibitor (10 mM NaF and 1 mM Na_3VO_4) and homogenated by thorough pipetting. The homogenate was transferred to a 1 ml syringe and sheared by passing the entire content through a 25G needle for four times. Samples were centrifuged at 1000xg for 10 min at 4°C. SN was discarded and the precipitates were resuspended in buffer B (buffer A without NP-40) and incubated on ice for 15 min with occasional mixing, followed by centrifugation as described above. Precipitates were resuspended in buffer C (buffer A containing 1% (v/v) NP-40), incubated on ice for 60 min with occasional mixing and centrifuged at 16000xg for 30 min at 4°C. The supernatant was collected and stored at -80°C until use.

5. Proteome analysis

a. Proteome quantification of low HeLa and SJSA1 sample amounts:

We first tested different protein amount dilutions of HeLa (cervical cancer cell line, a standard cell line in mass spectrometry analysis for test and optimization) and SJSA1 (human osteosarcoma tissue-derived line) cells to explore the sensitivity of our workflow for low sample amounts (Supplement Figure 1A). The HeLa and SJSA1 cells were cultured in Dulbecco's modified Eagle's medium at 10% fetal bovine serum, 20 mM glutamine and 1% penicillin–streptomycin, freshly collected and washed on ice with cold PBS. The cells were lysed in 0.2% sodium deoxycholate (SDC) -based buffers (0.2% SDC, 10 mM Tris pH8.5, 4 mM CAA and 1 mM TCEP) compatible for direct cell lysis and protease digestion conditions. The peptide concentrations were determined by BCA assay. Given that the protein amount of a single HeLa cell is equivalent to ~100-150 pg (Volpe and Eremenko-Volpe, 1970), we prepared the samples in a dilution serious of 10 ng, 20 ng, and 50 ng each to reflect ~ 100, 200 and 500 cells. Samples were acquired using nano flow liquid chromatography, separating peptides at an active LC gradient of 100 min, coupled to TIMS TOF pro-2 utilizing the diaPASEF mode and analyzed by DIA-NN software (see below). We quantified more than 7000 proteins in all replicates from 10 ng of HeLa and SJSA1 cells at 1% protein and peptide FDR (Supplement Figure 1B) and the quantification depth increased with dilution amounts. Collectively, we quantified 9547 proteins (Supplement Figure 1B), on an average more than 6000 unique proteins per sample (Supplement Figure 1C) with average peptide miss cleavage rate at less than 6% (Supplement Figure 1D). Further, the quantification reproducibility between the replicates was excellent as inferred from the Pearson correlation matrix (Pearson r=0.98, Supplement Figure 1E). Principle component analysis of samples clearly separated cell lines and cells by dilution serious (Supplement Figure 1F). The protein components separating the two cell lines in the PCA clearly distinguished the biological features of the cell type. This is driven by known protein markers such as TP53I11, THY1, DDX3Y & ITGB3 in SJSA1 and SERPINA10, SLC27A6, MAL2 in HeLa cells, thus allowing confident dissection of the biology of interest (Supplement figure 1G). With this, we show that our sensitive workflow at low sample amount can capture ultra-deep proteome and can help to dissect the biology of interest.

b. Label-retaining cell proteome:

The enriched LRC and non-LRC cells (by FACS sorting) were lysed in 0.2% SDC buffer (0.2% SDC, 10 mM Tris pH8.5, 4 mM CAA and 1 mM TCEP), boiled at 95°C and sonicated for 5 min on a Biorupter plus (Diagenode). Samples were digested with a

mixture of proteases LysC (1:100 ratio) and Trypsin (1:100 ratio) overnight at 37°C. To the digested peptide volume, five times volume of Isopropanol/1% TFA was added and vortexed to stop the digestion. The peptides were de-salted on equilibrated styrenedivinylbenzene-reversed phase sulfonated (SDB-RPS) StageTips, washed once in isopropanol/1% TFA and once with 0.2% TFA. Purified peptides were eluted twice with 20 µl of elution buffer (80%, 1.25% NH_4OH). The dried elutes were resuspended in MS loading buffer (3% ACN, 0.3% TFA) and stored at -20°C until MS measurement.

c. Data independent - PASEF acquisition runs:

Liquid chromatography was performed with an EASY nanoLC 1200 (Thermo Fisher Scientific) coupled online to a hybrid TIMS quadrupole TOF 2 mass spectrometer (Bruker timsTOF Pro2) via a CaptiveSpray nano-electrospray ion source and samples were measured in diaPASEF mode. Samples were loaded onto a 40-cm reversed-phase column (75 µm inner diameter, packed in house with ReproSil-Pur C18-AQ 1.9 µm resin; Dr Maisch). Peptides were separated in 120 min at a flow rate of 300 nl min^{-1}. Mobile phases buffer A and buffer B were 0.1% formic acid (FA) and 99.9% ddH_2O and 0.1% FA, 80% ACN, and 19.9% ddH_2O, respectively. Buffer B was linearly increased from 5% to 30% in 95 min, followed by an increase to 60% in 10 min and a further increase to 95% in 10 min, before re-equilibration for 5 min at 5% buffer B. For the calibration of ion mobility dimension, TIMS elution voltages were calibrated linearly to obtain the reduced ion mobility coefficients ($1/K_0$) using three Agilent ESI-Low Tuning Mix ions (*m/z* 622, 922 and 1,222). As we had very few thousand cells and concentrations could not be determined, the mass spectrometer was operated in low sample injection mode. For sample injection, the diaPASEF windows scheme was ranging in dimension m/z from 400 to 1400 and in dimension 1/K0 0.69(0.6)–1.47 (1.6), with 32 windows with TIMS functioning at Scan range 100-1700 m/z, Ramp Time 100 ms, Duty cycle 100%, Cycle time 100.00 ms and Spectra Rate 9.52 Hz.

d. diaPASEF data processing:

We used DIA-NN (version 1.8.1) which has the TIMS module incorporated to process the data following previously published recommendation (Demichev et al., 2022). DIA-NN was operated with maximum mass accuracy tolerances set to 10 ppm for both MS1 and MS2 spectra. Library generation was set to FASTA digest for library search, Deep learning-based spectra, RT, and IM profiling. Match between run (MBR) was enabled.

Quantification mode was set to Robust LC (high precision). The data were searched for proteins and peptides against Uniprot Human (version 2018) fasta file with a false discovery rate of less than 1% at the levels of protein and peptide. All other settings were left default. Enabled default parameters were: Protease (Trypsin/P), Missed cleavages (1), Maximum number of variable modification (null), peptide length (7-44) and precursor charge range (1-4). The output was filtered at precursor *q* value <1% and global protein *q* value <1%. When reporting protein numbers and quantities, the Protein.Group column in DIA-NN's was used to identify the protein group and the PG.MaxLFQ column was used to obtain the normalized quantity.

e. Proteome of acute leukemia cell lines, ALL PDX cells and ADAM10 KO cells:

Cells were lysed in 1% SDC buffer (1% SDC, 100 mM Tris pH8.5, 40 mM CAA and 10 mM TCEP), incubated on ice for 20 min, boiled at 95°C, sonicated for 10 min on a Biorupter plus (Diagenode) as described previously (Jayavelu et al., 2022). Samples amount equivalent to 10 ug (protein concentration determined by BCA assay) were digested with a mixture of proteases LysC (1:100 ratio) and Trypsin (1:100 ratio) overnight at 37°C. To the digested peptide volume, five times volume of Isopropanol/1% TFA was added and vortexed to stop the digestion. The peptides were de-salted on equilibrated styrenedivinylbenzene-reversed phase sulfonated (SDB-RPS) StageTips, washed once in isopropanol/1% TFA and once with 0.2% TFA. Purified peptides were eluted twice with 45 µl of elution buffer (80%, 1.25% NH_4OH). The dried elutes were resuspended in MS loading buffer (3% ACN, 0.3% TFA), peptide concentration determined by nanodrop device and stored at -20°C until MS measurement.

f. ADAM10 KO secretome:

SEM and Nalm-6 cells expressing CRISPR guides, a non-targeting sgRNA or an sgRNA targeting ADAM10 were washed several times in PBS to remove FBS and residual phenol red present in the culture medium. Cells were cultured in RPMI medium without phenol red, FBS and supernatant was collected after 18 h for secretome analysis. The supernatants were briefly centrifuged, filtered to remove debris, and processed for protein extraction. The samples were mixed 1:1 (50ul each) with 1% SDC buffer (1% SDC, 100 mM Tris pH8.5, 40 mM CAA and 10 mM TCEP),

boiled at 95°C, sonicated for 10 min on a Biorupter plus (Diagenode). Proteins were digested with a mixture of proteases LysC (1:100 ratio) and Trypsin (1:100 ratio) overnight at 37°C. To the digested peptide volume, five times volume of Isopropanol/1% TFA was added and vortexed to stop the digestion. The digested peptides were further processed as described above.

g. Liquid chromatography and data-dependent acquisition (DDA) runs:

LC-MS/MS analysis was performed with an EASY nanoLC 1200 (Thermo Fisher Scientific) interfaced online to a Q Exactive HF-X Hybrid Quadrupole-Orbitrap Mass Spectrometer (Thermo Fischer Scientific) via a nano electrospray ion source. Samples were loaded onto a 50-cm reversed-phase column (75 μm inner diameter, packed in house with ReproSil-Pur C18-AQ 1.9 μm resin (Dr. Maisch)). The column temperature was maintained at 60 °C using a homemade column oven. A binary buffer system, consisting of buffer A (0.1% FA) and buffer B (80% ACN plus 0.1% FA) was used for peptide separation at a flow rate of 300 nl min^{-1}. For proteome measurements, approximately 300 ng of peptides were loaded and separated in a 100 min gradient starting at 5% buffer B followed by a stepwise increase to 30% in 80 min, 60% in 4 min and 95% in 4 min. The buffer B concentration stayed at 95% for 4 min, decreased to 5% in 4 min and stayed there for 4 min. Mass spectra were acquired in a data-dependent mode; briefly, with one full scan at a target of $3e^6$ ions (300-1650 m/z, R=60,000 at 200 m/z), followed by Top15 MS/MS scans with HCD (high energy collisional dissociation) (target 1e5 ions, maximum filling time 28ms, Isolation window 1.4 m/z, and normalized collision energy 27), detected in the Orbitrap at a resolution of 15,000. Dynamic exclusion 30 s and charge exclusion (unassigned, 1, 6, -8 & >8) were enabled.

h. DDA data processing:

The cell line proteome and secretome MS raw files were processed using Maxquant (Cox and Mann, 2008) version 1.5.5.2 supported by Andromeda search engine. The data was searched for proteins and peptides using a target-decoy approach with a reverse database against Uniprot Human (version 2018) fasta file with a false discovery rate of less than 1% at the levels of protein and peptide. Enabled default settings such as oxidized methionine (M), acetylation (protein N-term), and carbamidomethyl (C) as fixed modification and Trypsin/P as enzyme specificity. A maximum of two missed cleavages were allowed, and a minimum peptide length of

seven amino acids set. The proteins were assigned to the same protein groups if two proteins could not be discriminated by unique peptides. The label-free quantification was performed using the MaxLFQ algorithm (Cox et al., 2014) and match between run (MBR) features was enabled for identification of peptide across runs based on mass accuracy and normalized retention times. For label free protein quantification minimum ratio count was set to 2.

i. Proteome data analysis:

The Maxquant and DIA-NN output table were analyzed in Perseus (Tyanova et al., 2016), prior to the analysis contaminants marked as reverse hits, contaminants and only identified by site-modification were filtered out. Two-sample t-test by permutation-based FDR and p-values were tested. For the enrichment analysis, enrichment terms such as keywords, GOCC, GOMF, GOBP, and KEGG name were used to show a systematic enrichment or de-enrichment compared with the distribution of all expression values. Fisher's exact tests were performed to detect the systematic enrichment or de-enrichment of annotations and pathways by analyzing proteins whose levels are significantly regulated upon different conditions (we used keywords GOCC, GOMF, GOBP, and KEGG name). The Benjamini–Hochberg FDR represents the degree of significance and the enrichment factor the level of enrichment compared with the background. Networks were generated using Cytoscape by uploading significantly enriched proteins.

In the LRC vs nLRC significant test analysis, ADAM10 showed p-value 0.0009990978 and q-value 0.0651 (with 1.7-fold higher expression in LRC cells). To further test ADAM10 as robust and highly reproducible protein marker for LRC, we performed machine learning based classification to find protein features. In the supervised learning, we trained with KNN classification algorithm. The classifier (Spearmen distance and cross validation type leave one out) ranked ADAM10 at 36 in a 49 protein based feature with error rate less than 10%.

6. Flow cytometry

Flow cytometry measurements were performed using the BD LSR Fortessa X-20 (BD, USA) according to the manufacturer's protocol.

For standard surface staining, cells were washed once using PBS, before the recommended amount of directly conjugated antibody was added. After 15 min incubation at RT, excess antibody was washed away using PBS, before cells were resuspended in PBS and measured.

To stain intracellular proteins, the FIX & PERM Cell Fixation & Cell Permeabilization Kit (GAS004, Thermo Fischer Scientific, USA) was used according to the manufacturer's protocol, but without using sodium azide.

7. Cell culture and *in vitro* assays

a. Cell culture:

Nalm-6 (ACC128, DSZM, Braunschweig, Germany; obtained May 3, 2012) and SEM B-ALL cell lines (ACC546, DSMZ, Braunschweig, Germany; obtained November 17, 2010) were cultured in RPMI medium (21875034, Thermo Fischer Scientific, USA) supplemented with 10% Fetal Bovine Serum (FBS), 1% L-Glutamine (Sigma-Aldrich, USA), 1% HEPES (4-(2-hydroxyethyl)-1-piperazineethanesulfonic acid, 83264, Sigma-Aldrich, USA), 1 mM sodium pyruvate (S8636, Sigma-Aldrich, USA). HEK293T cells (293T/17; American Type Cancer Collection (ATCC), USA; obtained May 2, 2013) were cultured in a DMEM medium (11965084, Thermo Fischer Scientific, USA) containing 10% FBS.

Serum starvation of Nalm-6 and SEM was performed in RPMI without containing L-glutamine, but otherwise as described above.

ALL PDX cells were cultured in StemSpan Serum-Free Expansion Medium (09650, STEMCELL Technologies, Canada) at a density of 10^7-1.5 x10^7 per ml and the medium was refreshed every 72 h. All cells were maintained at 37°C in presence of 5% CO_2.

AML PDX cells were cultured in StemPro-34 medium (according to Wermke, Dresden, Blood 2015) at a density of 1-2x10^6 per ml and the medium was refreshed every 72 h. All cells were maintained at 37°C in presence of 5% CO_2.

b. *In vitro* competitive assay:

Transduction, enrichment and mixing was performed as for the *in vivo* assay (see section 2g.). Mixed PDX cells were cultured *in vitro* for the same time period as the PDX cells of the same sample *in vivo*. Due to the limitations in culturing ALL PDX

samples *in vitro*, for ALL-199 the experiment was ended after 14 days. At the experimental endpoint, the distribution of KO vs. control population was measured by flow cytometry.

c. Cell cycle analysis:

Cells were harvested by pipetting, washed in PBS and stained using the ADAM10-APC antibody for 20 min at RT. Samples were washed with 1 ml PBS and centrifuged at 1200 rpm for 10 min. Cells were fixed with 0.5% paraformaldehyde (PFA) for 15 min on ice and washed twice with PBS. 0.1% Triton X-100 solution containing 1ug/ml DAPI was used to permeabilize the cells and stain the DNA content. Samples were recorded by flow cytometry (BD LSRFortessa™ X-20) and analyzed using the Dean-Jett-Fox model of the cell cycle tool of FlowJo software (version 10.6.2)

d. Colony-forming unit (CFU) assay:

CFU assays were performed using MethoCult™ H4034 Optimum (STEMCELL Technologies) according to manufacturer's specifications and frozen in aliquots. 1 ml cell suspension was prepared in StemPro™-34 SFM Medium, containing ten times the number of cells required for the desired plating density. 100 µl of cell suspension per 1 ml of methylcellulose was added and mixed thoroughly by vortexing. Using a 16G blunt-end needle and the respective 3cc syringe, 1.1 ml of the prepared aliquot was plated in a well from a meniscus-free 6-well plate (SmartDish™, STEMCELL Technologies) in replicates. The plate was incubated at 37°C, in 5% CO_2 with >95% humidity for 10 days, until colonies were scored. For colony scoring, a 6-well plate grid (STEMCELL Technologies) and an inverted microscope (Primovert, Zeiss) with the 4X and 10X magnifications were used. A colony was defined as a round cluster containing more than 20 cells, exhibiting an expanding morphology like a Granulocyte-Macrophage Colony-Forming Unit (GM-CFU). A well was considered as over-plated when there were more than 200 colonies per well, however scoring was only excluded if confluent colonies were abundantly present; a well was considered under-plated, when there were less than 25 colonies.

For the CFU assay with ADAM10 inhibitor, $1x10^6$ AML PDX cells freshly isolated from spleens were cultivated in the presence of the selective ADAM10 inhibitor GI254023X (Absource, 100 µM) or Dimethyl sulfoxide (DMSO, Sigma-Aldrich) for 72 h. $3x10^5$ cells were used for immunostaining of ADAM10 or isotype (APC Mouse IgG1, kappa

Isotype, Biozol Diagnostica) and evaluated by flow cytometry; rest of cells were subjected to the CFU assay, in the absence of the ADAM10 inhibitor.

For CFU assays with human CD34+ cells, human CD34+ blood progenitor cells (Lonza) from healthy donors were cultured in Iscove's Modified Dulbecco's Medium (PAN-Biotech) supplemented with 20% FCS, 100 U/ml Penicillin, 100 µg/ml Streptomycin, 2 mM L-Glutamine, IL-3 (10 ng/ml), IL-6 (20 ng/ml), SCF (20 ng/ml), TPO (20 ng/ml), FLT3-L (20 ng/ml) and GM-CSF (20 ng/ml). Cells were treated with either GI254023X (Absource, 100µM), Aderbasib (MedChemExpress, 10µM or 100 µM) or DMSO (Sigma-Aldrich) for 72 h. After confirming ADAM10 downregulation by flow cytometry, $2x10^3$ cells were plated in methylcellulose (MethoCultä H4534, STEMCELL Technologies) and counted as described above.

e. Apoptosis assay:

Freshly isolated PDX cells were treated with DMSO or GI254023X (#S8660, Selleckchem, Houston, USA) and cultured *in vitro* for three days. For the apoptosis staining, cells were harvested and transferred to 1x Annexin V binding buffer (LEI-A433-500ML, Annexin V Binding Buffer, Biozol, Eching, Germany). Cells were stained for ADAM10 (#352706, APC anti-human CD156c (ADAM10), Biolegend, San Diego, USA) and Annexin V (#563972, BV711 Annexin V, BD Horizon, New Jersey, USA) for 10 min, before DAPI (#D9542, DAPI, Sigma-Aldrich Chemie GmbH, Taufkirchen, Germany) was added for another 5 min and the sample was measured using flow cytometry. For apoptosis assays in ADAM10 KO, freshly isolated PDX cells were electroporated with RNP complexes containing either a sgRNA targeting ADAM10 or a CTRL sgRNA (Alt-R S.p. Cas9 Nuclease V3, Alt-R CRISPR-Cas9 tracrRNA, Alt-R CRISPR-Cas9 crRNA, IDT, Leuven, Belgium) using the 4D-Nucleofector using the program # CA137 and the P3 Primary Cell 4D-Nucleofector® X Kit L (Lonza Group, Basel, Switzerland).

f. *In vitro* chemotherapy assay:

For *in vitro* chemotherapy assay, freshly isolated PDX cells harboring either a GFP or mCherry tagged E-Firefly Luciferase were electroporated with RNP complexes containing either a sgRNA targeting ADAM10 or a CTRL sgRNA, respectively (Alt-R S.p. Cas9 Nuclease V3, Alt-R CRISPR-Cas9 tracrRNA, Alt-R CRISPR-Cas9 crRNA, IDT, Leuven, Belgium) using the 4D-Nucleofector using the program # CA137 and the

P3 Primary Cell 4D-Nucleofector® X Kit L (Lonza Group, Basel, Switzerland). Three days after electroporation cells were mixed and around 60.000 cells per well were seeded into 48-well plates (Thermo Fisher Scientific, USA). Per PDX sample, chemotherapeutic agent (Cytarabine, Cell Pharma GmbH, Bad Vilbel, Germany; Daunorubicin, Pfizer, Ney York City, USA; Doxorubicin, Medac GmbH, Wedel, Germany) and concentration of the respective agent, three wells were seeded as technical replicates. Four days after addition of the chemotherapeutic agents, cells were harvested and analyzed using flow cytometry.

8. Online resources and software

Bloodspot database (https://servers.binf.ku.dk/bloodspot/, (Bagger et al., 2019)) was used for evaluating ADAM10 expression in different ALL and AML subtypes in the dataset Leukemia MILE study (202603_at, GSE13159). Raw data was downloaded and the order adapted using Prism (GraphPad Software, USA; version 9.2). Overall survival was evaluated in the TCGA AML dataset (202603_at).

GSEA was performed on transcriptome data using the web-resource (http://www.gsea-msigdb.org/gsea/index.jsp, (Mootha et al., 2003; Subramanian et al., 2005)) analyzing the data for altered GO terms and KEGG pathways.

The effect of ADAM10 in other tumor entities was evaluated using the Human protein atlas web-resource (https://www.proteinatlas.org, version 21.1, (Uhlén et al., 2015)) in the pathology section.

GO networks were analyzed using Cytoscape (v3.9.0) (Bindea et al., 2009; Shannon et al., 2003).

Graphs were generated and t-test analyses performed using GraphPad Prism (Version 9, GraphPad Software, Inc., USA).

Flow cytometry data were analyzed using the FlowJo Software (FlowJo™ Software, version 10.7, Ashland, USA).

Schemes were created with BioRender.com (https://biorender.com).

9. Data availability

The mass spectrometry proteomics data have been deposited to the ProteomeXchange Consortium via the PRIDE (Perez-Riverol et al., 2022) partner repository with the dataset identifier PXD036223.

10. References

Bagger, F.O., Kinalis, S., and Rapin, N. (2019). BloodSpot: a database of healthy and malignant haematopoiesis updated with purified and single cell mRNA sequencing profiles. Nucleic acids research *47*, D881-D885.

Bindea, G., Mlecnik, B., Hackl, H., Charoentong, P., Tosolini, M., Kirilovsky, A., Fridman, W.-H., Pagès, F., Trajanoski, Z., and Galon, J. (2009). ClueGO: a Cytoscape plug-in to decipher functionally grouped gene ontology and pathway annotation networks. Bioinformatics *25*, 1091-1093.

Brinkman, E.K., Chen, T., Amendola, M., and van Steensel, B. (2014). Easy quantitative assessment of genome editing by sequence trace decomposition. Nucleic Acids Res *42*, e168. 10.1093/nar/gku936.

Cox, J., Hein, M.Y., Luber, C.A., Paron, I., Nagaraj, N., and Mann, M. (2014). Accurate proteome-wide label-free quantification by delayed normalization and maximal peptide ratio extraction, termed MaxLFQ. Molecular & cellular proteomics *13*, 2513-2526.

Cox, J., and Mann, M. (2008). MaxQuant enables high peptide identification rates, individualized ppb-range mass accuracies and proteome-wide protein quantification. Nature biotechnology *26*, 1367-1372.

Demichev, V., Szyrwiel, L., Yu, F., Teo, G.C., Rosenberger, G., Niewienda, A., Ludwig, D., Decker, J., Kaspar-Schoenefeld, S., and Lilley, K.S. (2022). dia-PASEF data analysis using FragPipe and DIA-NN for deep proteomics of low sample amounts. Nature communications *13*, 1-8.

Ebinger, S., Ozdemir, E.Z., Ziegenhain, C., Tiedt, S., Castro Alves, C., Grunert, M., Dworzak, M., Lutz, C., Turati, V.A., Enver, T., et al. (2016). Characterization of Rare, Dormant, and Therapy-Resistant Cells in Acute Lymphoblastic Leukemia. Cancer Cell *30*, 849-862. 10.1016/j.ccell.2016.11.002.

Jayavelu, A.K., Wolf, S., Buettner, F., Alexe, G., Häupl, B., Comoglio, F., Schneider, C., Doebele, C., Fuhrmann, D.C., Wagner, S., et al. (2022). The proteogenomic subtypes of acute myeloid leukemia. Cancer Cell *40*, 301-317.e312. 10.1016/j.ccell.2022.02.006.

Kerppola, T.K. (2008). Bimolecular fluorescence complementation (BiFC) analysis as a probe of protein interactions in living cells. Annu Rev Biophys *37*, 465-487. 10.1146/annurev.biophys.37.032807.125842.

Li, W., Xu, H., Xiao, T., Cong, L., Love, M.I., Zhang, F., Irizarry, R.A., Liu, J.S., Brown, M., and Liu, X.S. (2014). MAGeCK enables robust identification of essential genes

from genome-scale CRISPR/Cas9 knockout screens. Genome Biol *15*, 554. 10.1186/s13059-014-0554-4.

Liu, W.-H., Mrozek-Gorska, P., Wirth, A.-K., Herold, T., Schwarzkopf, L., Pich, D., Völse, K., Melo-Narváez, M.C., Carlet, M., Hammerschmidt, W., and Jeremias, I. (2020). Inducible transgene expression in PDX models in vivo identifies KLF4 as a therapeutic target for B-ALL. Biomarker Research *8*, 46. 10.1186/s40364-020-00226-z.

Mootha, V.K., Lindgren, C.M., Eriksson, K.-F., Subramanian, A., Sihag, S., Lehar, J., Puigserver, P., Carlsson, E., Ridderstråle, M., Laurila, E., et al. (2003). PGC-1α-responsive genes involved in oxidative phosphorylation are coordinately downregulated in human diabetes. Nature Genetics *34*, 267-273. 10.1038/ng1180.

Patro, R., Duggal, G., Love, M.I., Irizarry, R.A., and Kingsford, C. (2017). Salmon provides fast and bias-aware quantification of transcript expression. Nat Methods *14*, 417-419. 10.1038/nmeth.4197.

Perez-Riverol, Y., Bai, J., Bandla, C., García-Seisdedos, D., Hewapathirana, S., Kamatchinathan, S., Kundu, D.J., Prakash, A., Frericks-Zipper, A., and Eisenacher, M. (2022). The PRIDE database resources in 2022: a hub for mass spectrometry-based proteomics evidences. Nucleic acids research *50*, D543-D552.

Schindelin, J., Arganda-Carreras, I., Frise, E., Kaynig, V., Longair, M., Pietzsch, T., Preibisch, S., Rueden, C., Saalfeld, S., and Schmid, B. (2012). Fiji: an open-source platform for biological-image analysis. Nature methods *9*, 676-682.

Shannon, P., Markiel, A., Ozier, O., Baliga, N.S., Wang, J.T., Ramage, D., Amin, N., Schwikowski, B., and Ideker, T. (2003). Cytoscape: a software environment for integrated models of biomolecular interaction networks. Genome research *13*, 2498-2504.

Subramanian, A., Tamayo, P., Mootha, V.K., Mukherjee, S., Ebert, B.L., Gillette, M.A., Paulovich, A., Pomeroy, S.L., Golub, T.R., and Lander, E.S. (2005). Gene set enrichment analysis: a knowledge-based approach for interpreting genome-wide expression profiles. Proceedings of the National Academy of Sciences *102*, 15545-15550.

Terziyska, N., Castro Alves, C., Groiss, V., Schneider, K., Farkasova, K., Ogris, M., Wagner, E., Ehrhardt, H., Brentjens, R.J., zur Stadt, U., et al. (2012). In vivo imaging enables high resolution preclinical trials on patients' leukemia cells growing in mice. PLoS One *7*, e52798. 10.1371/journal.pone.0052798.

Truong, D.J., Kuhner, K., Kuhn, R., Werfel, S., Engelhardt, S., Wurst, W., and Ortiz, O. (2015). Development of an intein-mediated split-Cas9 system for gene therapy. Nucleic Acids Res *43*, 6450-6458. 10.1093/nar/gkv601.

Tyanova, S., Temu, T., Sinitcyn, P., Carlson, A., Hein, M.Y., Geiger, T., Mann, M., and Cox, J. (2016). The Perseus computational platform for comprehensive analysis of (prote) omics data. Nature methods *13*, 731-740.

Uhlén, M., Fagerberg, L., Hallström, B.M., Lindskog, C., Oksvold, P., Mardinoglu, A., Sivertsson, Å., Kampf, C., Sjöstedt, E., and Asplund, A. (2015). Proteomics. Tissue-based map of the human proteome. Science (New York, NY) *347*, 1260419-1260419.

Volkmann, C., Brings, N., Becker, M., Hobeika, E., Yang, J., and Reth, M. (2016). Molecular requirements of the B-cell antigen receptor for sensing monovalent antigens. EMBO J *35*, 2371-2381. 10.15252/embj.201694177.

Volpe, P., and Eremenko-Volpe, T. (1970). Quantitative studies on cell proteins in suspension cultures. European journal of biochemistry *12*, 195-200.

Yang, J., and Reth, M. (2010). Oligomeric organization of the B-cell antigen receptor on resting cells. Nature *467*, 465-469. 10.1038/nature09357.
